# Differential regulation of hepatic macrophage fate by Chi3l1 in metabolic dysfunction-associated steatotic liver disease

**DOI:** 10.7554/eLife.107023

**Published:** 2026-06-26

**Authors:** Jia He, Bo Chen, Weiju Lu, Xiong Wang, Ruoxue Yang, Chengxiang Deng, Xiane Zhu, Keqin Wang, Lang Wang, Cheng Xie, Rui Li, Xiaokang Lu, Ruizhi Yang, Cheng Peng, Canpeng Li, Zhao Shan

**Affiliations:** 1 Yunnan Key Laboratory of Cell Metabolism and Diseases, Center for Life Sciences, School of Life Sciences, Yunnan University Kunming China; 2 Bio-X Center for Interdisciplinary Innovation, Yunnan University Kunming China; The Hebrew University of Jerusalem Israel; National Institute of Immunology India

**Keywords:** kupffer cells, monocytes-derived macrophages, MASLD, Chi3l1, Mouse

## Abstract

Metabolic dysfunction-associated steatotic liver disease (MASLD) progression involves the replacement of protective embryo-derived Kupffer cells (KCs) by inflammatory monocyte-derived macrophages (MoMFs), yet the regulatory mechanisms remain unclear. Here, we identify chitinase 3-like 1 (Chi3l1/YKL-40) as a critical metabolic regulator of hepatic macrophage fate. We observed high expression of Chi3l1 in both KCs and MoMFs during MASLD development. Genetic deletion of Chi3l1 specifically in KCs significantly exacerbated MASLD severity and metabolic dysfunction, whereas MoMF-specific Chi3l1 deletion showed minimal metabolic effects. Mechanistic studies revealed that this cell type-specific regulation arises from differential metabolic requirements: KCs display elevated glucose metabolism compared to MoMFs. Chi3l1 directly interacts with glucose to inhibit its cellular uptake, thereby selectively protecting glucose-dependent KCs from metabolic stress-induced cell death while having negligible effects on less glucose-dependent MoMFs. These findings uncover a novel Chi3l1-mediated metabolic checkpoint that preferentially maintains KCs populations through glucose metabolism modulation, providing important new insights into the pathogenesis of MASLD and potential therapeutic strategies targeting macrophage-specific metabolic pathways.

## Introduction

Metabolic dysfunction-associated steatotic liver disease (MASLD) has become the most prevalent chronic liver disorder in western populations, affecting approximately 30% of adults and driven by its strong association with obesity and metabolic syndrome (Hardy et al., 2016). The disease spectrum ranges from metabolic dysfunction-associated fatty liver (MAFL) to metabolic dysfunction-associated steatohepatitis (MASH), with the latter characterized by steatosis, inflammation, hepatocyte ballooning, and progressive fibrosis (Eslam et al., 2020). Central to MASLD pathogenesis are hepatic macrophages, particularly the embryo-derived Kupffer cells (KCs) that reside in liver sinusoids (Gomez Perdiguero et al., 2015; Kazankov et al., 2019). These self-renewing resident macrophages (Hashimoto et al., 2013) play crucial roles in lipid homeostasis, as evidenced by studies showing that depletion of CD207^+^ KCs leads to impaired triglyceride storage (Tran et al., 2020). As MASH progresses, dying KCs are progressively replaced by monocyte-derived macrophages (MoMFs) that exhibit heightened inflammatory properties and contribute to liver damage (Daemen et al., 2021; Tran et al., 2020). For example, one study demonstrated that in diet-induced MASH, KCs enhancer landscapes and gene expression profiles are profoundly reprogrammed (including up-regulation of Trem2 and Cd9) and KCs identity is lost, while MoMFs adopt convergent epigenomes, transcriptomes, and functions during macrophage recruitment and adaptation in MASH (Seidman et al., 2020). Another work showed that in MASLD the number of resident KCs declines and MoMFs accumulate; these recruited macrophages include subsets that either mirror homeostatic KCs or resemble lipid-associated macrophages (LAMs) from obese adipose tissue, with the LAM-type expressing osteopontin and localizing to fibrotic zones (Remmerie et al., 2020). Together, these findings highlight that this transition from protective embryo-derived KCs (EmKCs) to monocyte-derived KCs (MoKCs) represents a critical juncture in disease progression, yet the mechanisms regulating this shift remain poorly understood.

A key determinant of macrophage function is cellular metabolism. Macrophages dynamically switch between glycolytic and oxidative phosphorylation pathways to adapt to environmental changes (Green et al., 2014). During MASLD, hepatic macrophages increase their glycolytic activity, which may exacerbate inflammation and tissue damage (Dong et al., 2024; Inomata et al., 2022; Lodge et al., 2024). While glucose metabolism is known to influence macrophage polarization, its specific role in determining hepatic macrophage fate - particularly the balance between KCs and MoMFs - remains unknown. Chitinase 3-like 1 (Chi3l1/YKL-40) has emerged as an important regulator of macrophage biology, promoting cell survival through ERK1/2 and PI3K/Akt pathways while modulating anti-inflammatory cytokines like IL-10 (Dela Cruz et al., 2012; He et al., 2013; Lee et al., 2011; Lee et al., 2009; Zhou et al., 2015). However, its potential role in macrophage metabolic reprogramming, particularly in the context of hepatic glucose metabolism, has not been explored.

In this study, we identify a novel mechanism by which Chi3l1 governs hepatic macrophage fate through metabolic regulation. We demonstrate that Chi3l1 directly interacts with glucose to suppress its uptake in macrophages. Strikingly, this interaction selectively protects glucose-high KCs from cell death in MASLD conditions, while having minimal effect on glucose-low MoMFs. These findings reveal a previously unrecognized Chi3l1-mediated metabolic checkpoint that maintains KC populations, providing new insights into the pathogenesis of MASLD and potential therapeutic strategies.

## Results

### Hepatic macrophages express Chi3l1 and upregulate its expression post HFHC diet

In our previous study, we found that Chi3l1 expressed by hepatic macrophages influences macrophage function during acute liver injury (Shan et al., 2021). Therefore, we sought to determine whether Chi3l1 also plays a role in MASLD and whether its expression in hepatic macrophages is altered in this context. To this end, we first established a mouse model of MASLD by feeding C57BL/6 J wild-type (WT) mice a normal chow diet (NCD) or a high-fat, high-cholesterol (HFHC) diet for 16 weeks. Histological analysis of liver sections using Hematoxylin and Eosin (H&E) and Sirius Red staining revealed marked lipid accumulation without apparent fibrosis (Figure 1—figure supplement 1A). Consistently, western blot analysis showed no upregulation of α-SMA (Figure 1—figure supplement 1B), suggesting that our HFHC model represents an early stage of MASLD.

**Figure 1.**
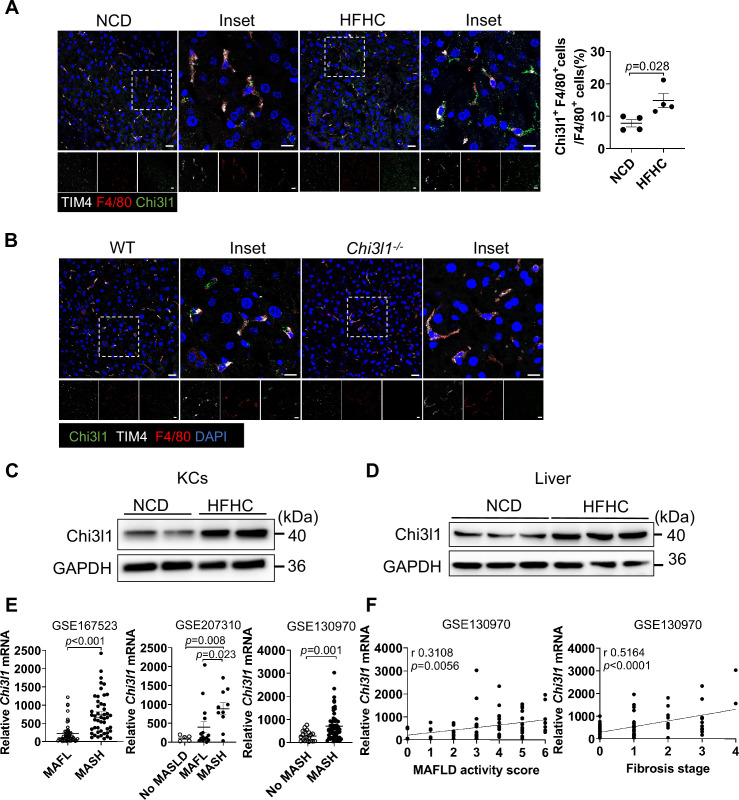
Hepatic macrophages express Chi3l1 and upregulate its expression post high-fat, high-cholesterol (HFHC) diet. (**A**) Immunofluorescent staining of TIM4 (white), F4/80 (red), Chi3l1 (green), and nuclear DAPI (blue) in liver sections of mice fed with either normal chow diet (NCD) or HFHC diet for 16 weeks, illustrating Chi3l1 expression in hepatic macrophages. Scale bar = 20 µm and 10 µm (Inset). Chi3l1^+^ F4/80^+^ cells/F4/80^+^ cells were statistically analyzed. n=4 mice/group. (**B**) Representative immunofluorescence images of liver sections from WT and *Chi3l1^-/-^* mice stained for Chi3l1 (green), F4/80 (macrophages), and TIM4 (Kupffer cells [KCs]). DAPI (blue) marks nuclei. Scale bar = 20 µm and 10 µm (Insets). (**C, D**) Western blot analysis of Chi3l1 in either isolated KCs (**C**) or whole liver tissue (liver, **D**) from mice fed either NCD or HFHC diet. n=2–3 mice/group. (**E**) mRNA expression levels of Chi3l1 in liver tissues of patients with metabolic dysfunction-associated fatty liver (MAFL) or with metabolic dysfunction-associated steatohepatitis (MASH; GEO datasets: GSE167523, GSE207310, GSE130970). No-MAFLD or healthy individuals serve as controls. (**F**) The correlation between mRNA expression levels of *Chi3l1* and MASLD activity score or fibrosis stage was analyzed (GEO datasets: GSE130970). Representative images were shown in A and B. Mann-Whitney test was performed in E. Pearson’s correlation was performed in F. p value and r value are as indicated. Figure 1—source data 1.Numerical data of Figure 1A and E–F. Figure 1—source data 2.PDF file containing original western blots for Figure 1C and D, indicating the relevant bands and treatments. Figure 1—source data 3.Original files for western blot analysis displayed in Figure 1C and D.

Next, we performed immunofluorescence staining for Chi3l1 in liver sections using antibodies against TIM4 (a KCs marker), F4/80 (a pan-macrophage marker), and Chi3l1. This confirmed that Chi3l1 is expressed in both KCs (TIM4^+^F4/80^+^ cells) and MoMFs (TIM4^-^F4/80^+^ cells), with elevated expression under HFHC conditions (Figure 1A). To validate the specificity of Chi3l1 staining, we generated *Chi3l1^-/-^* mice and confirmed knockout efficiency by qRT-PCR (Figure 1—figure supplement 2A, B). Immunofluorescence staining for Chi3l1 in liver sections from WT and *Chi3l1^-/-^* mice showed that the anti-Chi3l1 antibody specifically detected Chi3l1 in WT but not *Chi3l1^-/-^* mice, confirming the specificity of the staining (Figure 1B). We next assessed whether Chi3l1 is upregulated by HFHC feeding by measuring its protein levels in isolated KCs and whole liver tissue via western blotting. A marked increase in Chi3l1 expression was observed in both KCs and liver tissue following HFHC diet feeding (Figure 1C and D). Consistently, patients with MAFL or MASH exhibited elevated hepatic Chi3l1 mRNA levels, which correlated with MASLD severity and fibrosis stage (Figure 1E and F). These findings suggest that Chi3l1 is expressed in hepatic macrophages and may contribute to MASLD progression.

**Figure 1—figure supplement 1.**
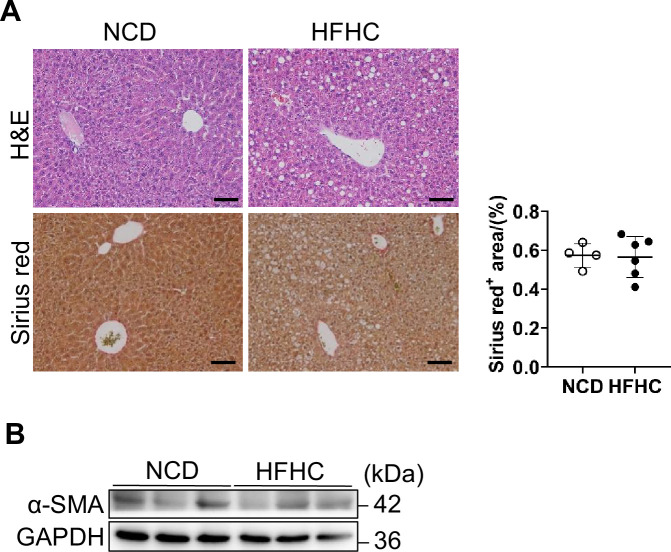
Metabolic dysfunction-associated steatotic liver disease progression in the high-fat, high-cholesterol (HFHC) diet-induced mouse model. (**A**) Representative liver sections from wild-type C57BL/6 J mice fed either a normal chow diet (NCD) or an HFHC diet for 16  weeks. H&E and Sirius Red staining were used to assess lipid deposition, inflammation, and fibrosis. Scale bar: 20  µm. Quantification of Sirius Red–positive area is shown. (**B**) Western blot analysis of α-SMA expression in whole liver lysates from NCD- and HFHC-fed mice (n = 3 mice/group) to evaluate activation of hepatic stellate cells. Figure 1—figure supplement 1—source data 1.Numerical data of Figure 1—figure supplement 1A. Figure 1—figure supplement 1—source data 2.PDF file containing original western blots for Figure 1—figure supplement 1B, indicating the relevant bands and treatments. Figure 1—figure supplement 1—source data 3.Original files for western blot analysis displayed in Figure 1—figure supplement 1B.

**Figure 1—figure supplement 2.**
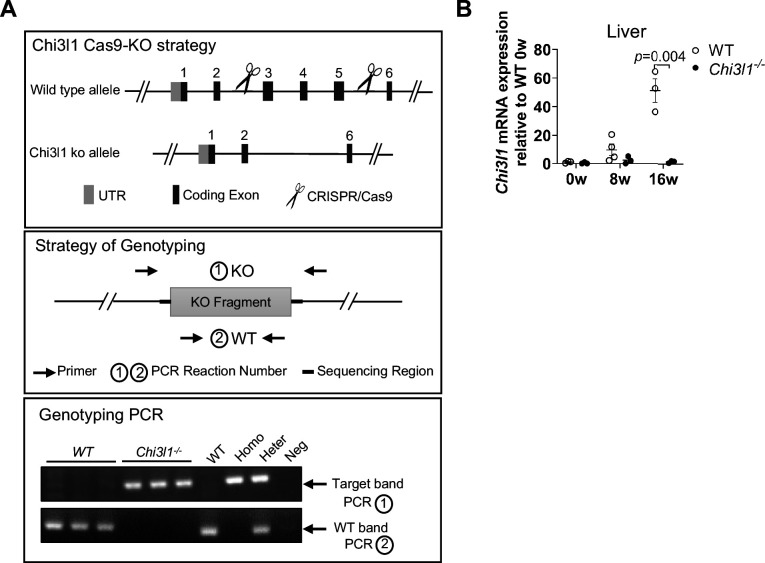
Generation and validation of *Chi3l1^-/-^* mice. (**A**) The construction, genotyping strategy, and genotyping results of *Chi3l1^-/-^* mice. P: positive control; WT: Wild-type; Neg: Blank control (ddH_2_O). (**B**) qRT-PCR analysis of mRNA expression levels of Chil1 in liver tissues of WT and *Chi3l1^-/-^* mice fed with HFHC for 0, 8, and 16 weeks. n=3–4 mice/group. Two-tailed, unpaired student t-test was performed in B. p value is as indicated. Figure 1—figure supplement 2—source data 1.Numerical data of Figure 1—figure supplement 2B.

### Deficiency of Chi3l1 in Kupffer cells promotes insulin resistance and hepatic lipid accumulation

**Figure 2.**
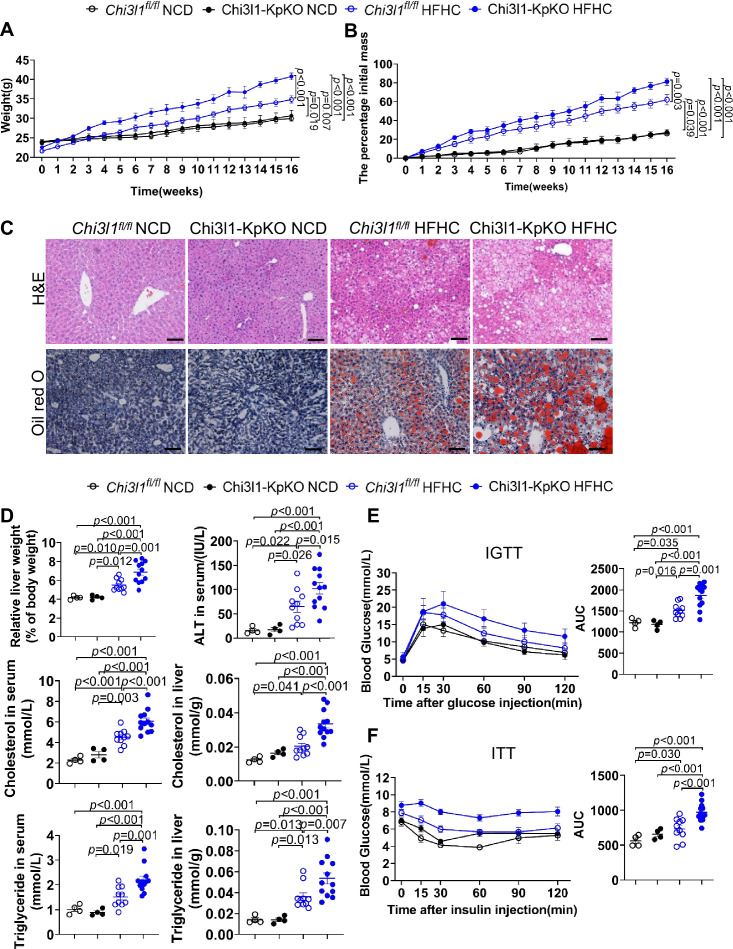
Deficiency of Chi3l1 in Kupffer cells promotes insulin resistance and hepatic lipid accumulation. *Chi3l1^fl/fl^* and Chi3l1-KpKO mice were fed either a normal chow diet (NCD) or a high-fat, high-cholesterol (HFHC) diet for 16 weeks. (**A, B**) Body weight was recorded during HFHC diet feeding (**A**) and expressed as a percentage of initial body mass (**B**). (**C**) H&E (Upper panel) and Oil Red O staining (Lower panel) was performed to examine liver histology and hepatic lipid accumulation in both genotypes after 16 weeks of NCD or HFHC diet. Scale bar = 20 µm. (**D**) Liver index (liver weight/body weight ×100%), ALT levels, and serum and liver cholesterol or triglyceride levels were measured in both genotypes after 16 weeks on NCD or HFHC diets. n=4–12 mice/group. (**E, F**) Intraperitoneal glucose tolerance test (IGTT) and insulin tolerance test (ITT) were performed after 16 weeks of NCD or HFHC feeding in both genotypes (n=4–12 mice per group). Representative images were shown in (**C**). One-way ANOVA was performed in (**A, B, D–F**). p value is as indicated. Figure 2—source data 1.Numerical data of Figure 1A–B and D–F.

Given that Chi3l1 is highly expressed in hepatic macrophages, we investigated its functional role by generating mice with conditional knockout (cKO) of *Chi3l1* in either KCs or MoMFs. First, we generated Chi3l1-KpKO mice by crossing *Chi3l1^fl/fl^* mice with *Clec4f-cre* mice (Scott et al., 2018), achieving KC-specific deletion of Chi3l1 (Figure 2—figure supplement 1A–C). These mice, along with *Chi3l1^fl/fl^* controls, were fed either a NCD or an HFHC diet. Under NCD feeding, Chi3l1-KpKO and *Chi3l1^fl/fl^* mice displayed comparable phenotypes in terms of body weight gain, hepatic lipid deposition, metabolic parameters, glucose tolerance, and insulin resistance (Figure 2A–F). In contrast, when fed an HFHC diet, Chi3l1-KpKO mice exhibited markedly accelerated weight gain compared to controls (Figure 2A and B). These mice also showed increased hepatic lipid accumulation, as evidenced by H&E and Oil Red O staining at 16 weeks (Figure 2C), along with greater metabolic disturbances, including a higher liver index (liver-to-body weight ratio), elevated serum ALT levels, and increased cholesterol and triglyceride levels in both liver and serum (Figure 2D). Furthermore, Chi3l1-KpKO mice exhibited impaired glucose metabolism, as indicated by worsened glucose tolerance and insulin resistance in IGTT and ITT assays (Figure 2E and F). To exclude potential off-target effects caused by *Clec4f-Cre* insertion, we compared *Clec4f-Cre* and Chi3l1-KpKO mice. The phenotypic differences between *Clec4f-Cre* and Chi3l1-KpKO mice mirrored those observed between *Chi3l1^fl/fl^* and Chi3l1-KpKO mice, with the latter showing faster weight gain, more severe hepatic steatosis, greater metabolic dysregulation, and worsened glucose intolerance and insulin resistance (Figure 2—figure supplement 2A–G).

**Figure 2—figure supplement 1.**
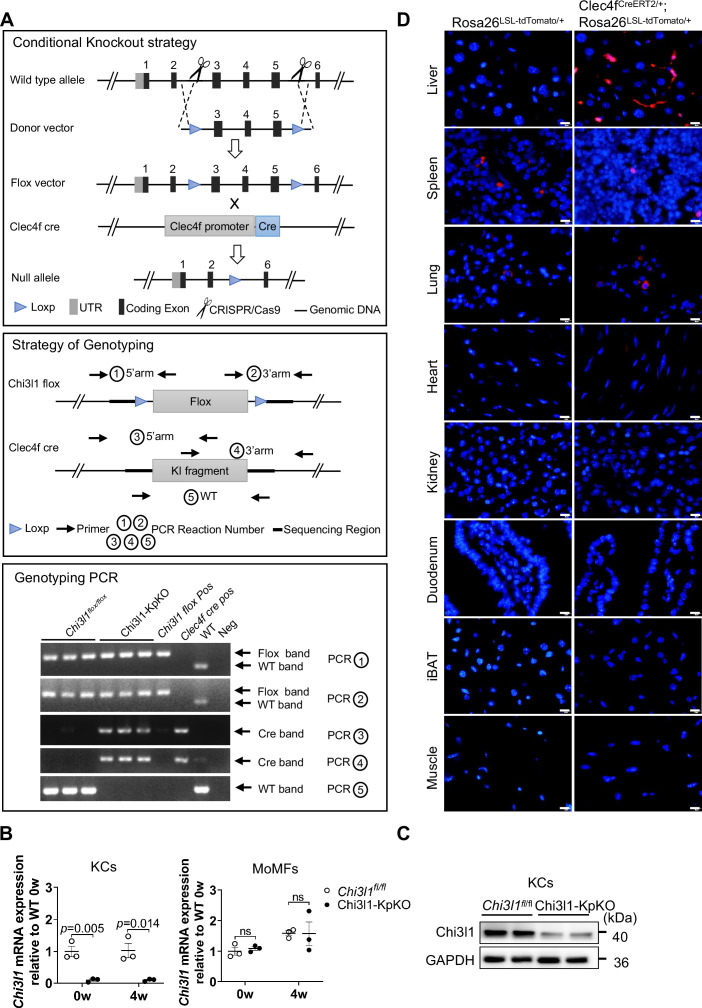
The construction and genotype of Chi3l1-KpKO mice (**A**) The construction, genotyping strategy, and genotyping results of Chi3l1-KpKO mice. P: positive control; WT: Wild-type; Neg: Blank control (ddH_2_O). (**B**) qRT-PCR analysis of mRNA expression levels of Chil1 in KCs (CD45^+^ F4/80^hi^ CD11b^low^ TIM4^hi^) or MoMFs (CD45^+^ F4/80^low^ CD11b^hi^ Ly6G^-^ TIM4^-^) FACS sorted from *Chi3l1^fl/fl^* and Chi3l1-KpKO mice at 0 and 4 weeks post HFHC diet. n=3 mice/group. (**C**) Western blot to detect Chi3l1 expression in isolated KCs of *Chi3l1^fl/fl^* and Chi3l1-KpKO mice. n=2 mice/group. (**D**) The expression specificity of Clec4f was examined in various tissues in Clec4f^CreERT2/+^; Rosa26^LSL-tdTomato/+^ mice, which is generated by crossing Clec4f^CreERT2/+^ with Rosa26^LSL-tdTomato/+^ mice. Figure 2—figure supplement 1—source data 1.Numerical data of Figure 2—figure supplement 1B. Figure 2—figure supplement 1—source data 2.PDF file containing original western blots for Figure 2—figure supplement 1C, indicating the relevant bands and treatments. Figure 2—figure supplement 1—source data 3.Original files for western blot analysis displayed in Figure 2—figure supplement 1C.

**Figure 2—figure supplement 2.**
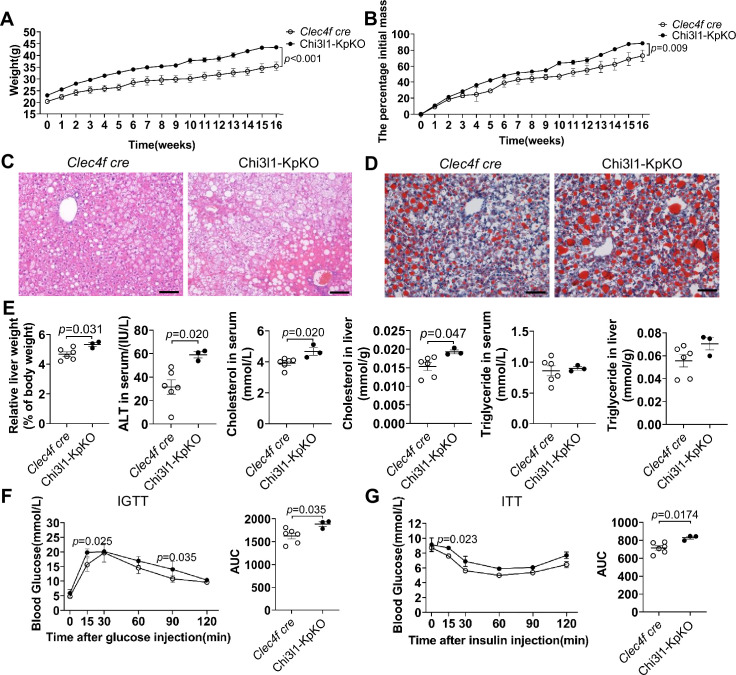
Deficiency of Chi3l1 in Kupffer cells promotes insulin resistance and hepatic lipid accumulation. *Clec4f cre* and Chi3l1-KpKO mice were fed with an HFHC diet for 16 weeks. (**A, B**) Body weight was recorded during HFHC diet feeding (**A**) and expressed as a percentage of initial body mass (**B**). (**C, D**) H&E (**C**) and Oil Red O staining (**D**) was performed to examine liver histology and hepatic lipid accumulation in both genotypes after 16 weeks of HFHC diet. Scale bar = 20 µm. (**E**) Liver index (liver weight/body weight × 100%), ALT levels, and serum and liver cholesterol or triglyceride levels were measured in both genotypes after 16 weeks of HFHC diet. n=3–6 mice/group. (**F&G**) Intraperitoneal glucose tolerance test (IGTT) and insulin tolerance test (ITT) were performed after 16 weeks of HFHC feeding in both genotypes. n=3–6 mice/group. Representative images were shown in C and D. Two-tailed, unpaired Student t-test was performed in A, B, and E–G. p value is as indicated. Figure 2—figure supplement 2—source data 1.Numerical data of Figure 2—figure supplement 2A–B and E-G.

**Figure 3.**
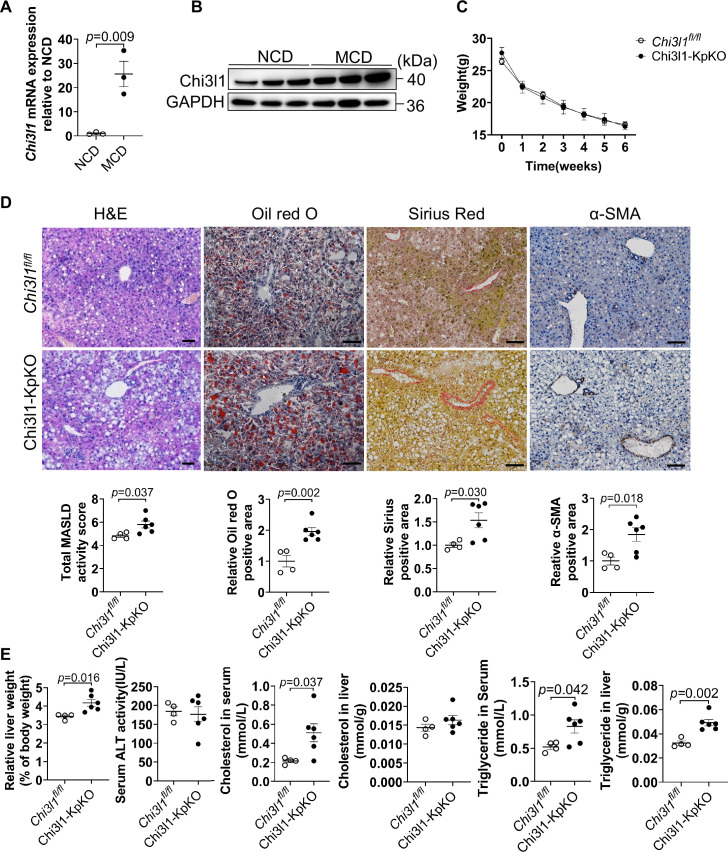
Deficiency of Chi3l1 in Kupffer cells (KCs) promotes liver steatosis and fibrosis in metabolic dysfunction-associated steatohepatitis. Male wild-type C57B/6 J mice were fed with normal chow diet (NCD) or methionine-choline deficient (MCD) diet for 6 weeks (**A–B**). *Chi3l1^fl/fl^* and Chi3l1-KpKO mice were fed with an MCD diet for 6 weeks (**C–E**). (**A, B**) qRT-PCR (**A**) and western blot (**B**) analysis of Chi3l1 expression in whole liver tissues under NCD and MCD diets. n=3 mice/group. (**C**) Body weight of mice with conditional deletion of *Chi3l1* in KCs (Chi3l1-KpKO) and their control mice (*Chi3l1^fl/fl^*) was recorded during MCD diet. (**D**) Histological analyses were performed in liver tissue of Chi3l1-KpKO and *Chi3l1^fl/fl^* fed the MCD diet for 6 weeks. Scale bar = 20 μm. (**E**) Liver index (liver weight/body weight ×100%), ALT levels, and serum and liver cholesterol or triglyceride levels were measured in both genotypes fed the MCD diet for 6 weeks. n=4–6 mice/group. Representative images are shown in D. Two-tailed, unpaired student t-test was performed in A, C, D, and E. p value is as indicated. Figure 3—source data 1.Numerical data of Figure 3A, C–D and E. Figure 3—source data 2.PDF file containing original western blots for Figure 3B, indicating the relevant bands and treatments. Figure 3—source data 3.Original files for western blot analysis displayed in Figure 3B.

To investigate the role of KCs-derived Chi3l1 in MASH, we first examined its expression in a methionine-choline deficient (MCD) diet model. WT mice fed an MCD diet for 6 weeks showed significantly increased Chi3l1 mRNA and protein levels in whole liver tissues compared to NCD controls, confirming diet-induced upregulation (Figure 3A and B). To assess the functional contribution of KCs-derived Chi3l1, we subjected Chi3l1-KpKO mice along with *Chi3l1^fl/fl^* controls to 6 weeks of MCD diet feeding. Body weight was comparable between genotypes throughout the feeding period (Figure 3C). Histological analysis revealed that loss of Chi3l1 in KCs led to a significant exacerbation of MCD diet-induced hepatic steatosis, inflammation, and fibrosis, as reflected by increased MASLD activity scores, Oil Red O staining, Sirius Red deposition, and α-SMA expression (Figure 3D). Consistent with this histological finding, Chi3l1-KpKO mice exhibited an increased liver index but similar serum ALT levels, reflecting liver weight gain without evidence of enhanced liver injury (Figure 3E). Additionally, these mice showed significant increases in serum and hepatic triglyceride levels, as well as elevated serum cholesterol, whereas hepatic cholesterol is not significantly upregulated compared to controls (Figure 3E). These data demonstrate that loss of Chi3l1 in KCs promotes hepatic steatosis, suggesting a protective role for KC-derived Chi3l1 in MASH pathogenesis.

**Figure 3—figure supplement 1.**
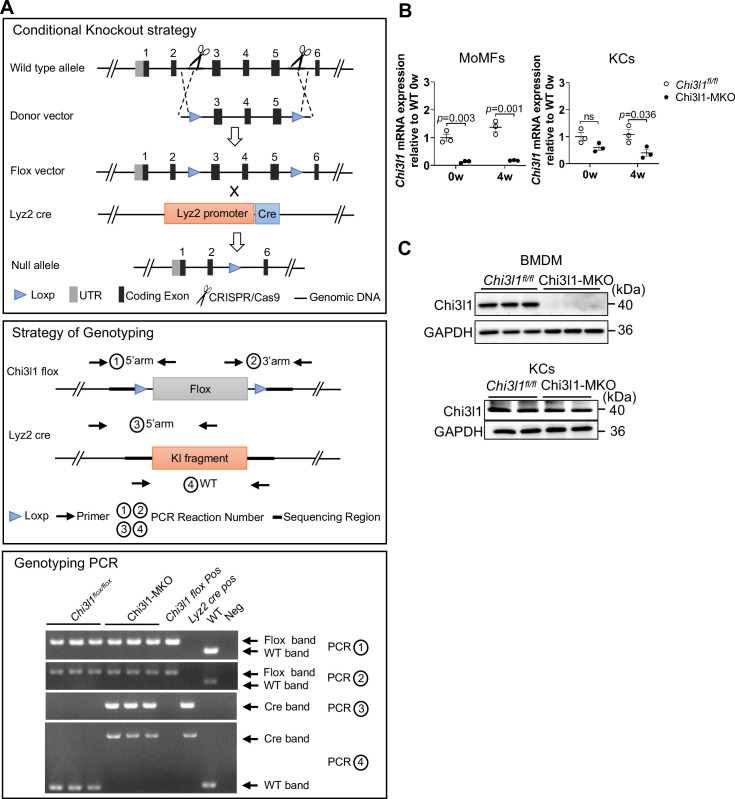
The construction and genotype of Chi3l1-MKO mice. (**A**) The construction, genotyping strategy, and genotyping results of Chi3l1-MKO mice. pos: positive control; WT: Wild-type; Neg: Blank control (ddH_2_O). (**B**) qRT-PCR analysis of mRNA expression levels of Chi3l1 in KCs (CD45^+^ F4/80^hi^ CD11b^low^ TIM4^hi^) or MoMFs (CD45^+^ F4/80^low^ CD11b^hi^ Ly6G^-^ TIM4^-^) FACS sorted from *Chi3l1^fl/fl^* and Chi3l1-MKO mice at 0 and 4 weeks post HFHC diet. n=3 mice/group. (**C**) Western blotting analysis of protein levels of Chi3l1 in bone-marrow-derived macrophage (BMDM) and primary KCs of *Chi3l1^fl/fl^* and Chi3l1-MKO mice. n=2–3 mice/group. Figure 3—figure supplement 1—source data 1.Numerical data of Figure 3—figure supplement 1B. Figure 3—figure supplement 1—source data 2.PDF file containing original western blots for Figure 3—figure supplement 1C, indicating the relevant bands and treatments. Figure 3—figure supplement 1—source data 3.Original files for western blot analysis displayed in Figure 3—figure supplement 1C.

**Figure 3—figure supplement 2.**
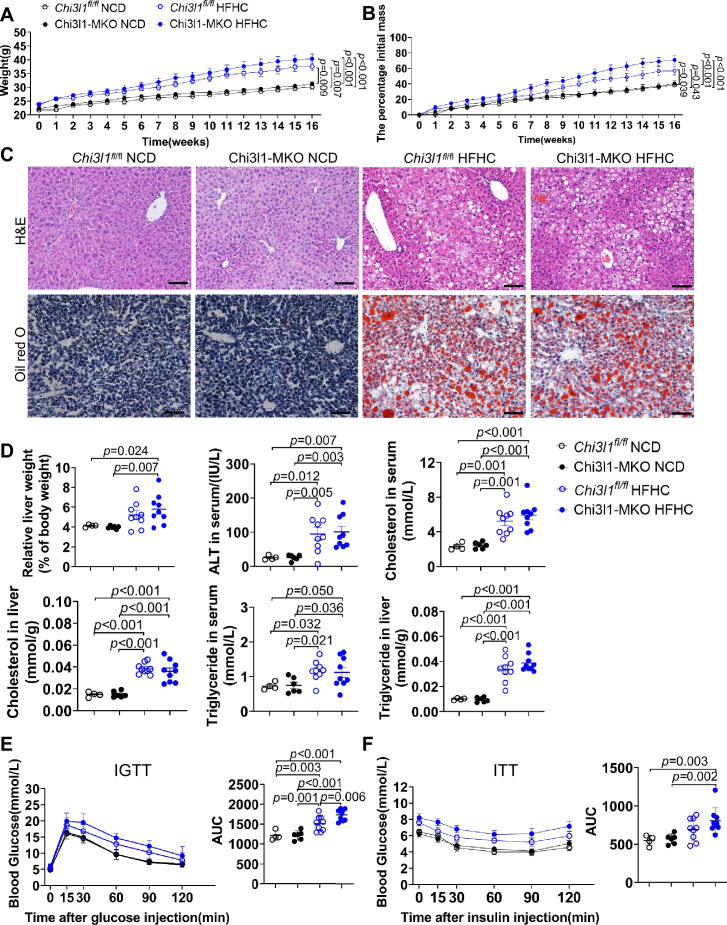
Deficiency of Chi3l1 in monocyte-derived macrophages barely affects insulin resistance and hepatic lipid accumulation. *Chi3l1^fl/fl^* and Chi3l1-MKO mice were fed either a normal chow diet (NCD) or a high-fat, high-cholesterol (HFHC) diet for 16 weeks. (**A, B**) Body weight was recorded during HFHC diet feeding (**A**) and expressed as a percentage of initial body mass (**B**). (**C**) H&E (upper panel) and Oil Red O staining (lower panel) was performed to examine liver histology and hepatic lipid accumulation in both genotypes after 16 weeks of NCD or HFHC diet. Scale bar = 20 µm. (**D**) Liver index (liver weight/body weight ×100%), ALT levels, and serum and liver cholesterol or triglyceride levels were measured in both genotypes after 16 weeks on NCD or HFHC diets. n=4–9 mice/group. (**E, F**) Intraperitoneal glucose tolerance test (IGTT) and insulin tolerance test (ITT) were performed after 16 weeks of NCD or HFHC feeding in both genotypes (n=4–9 mice per group). Representative images were shown in (**C**). One-way ANOVA was performed in (**A, B, D–F**). p values are as indicated.

To assess the role of Chi3l1 in MoMFs, we generated Chi3l1-MKO mice (*Chi3l1^fl/fl^*×*Lyz2-Cre* Scott et al., 2018) and validated Chi3l1 deletion efficiency in MoMFs and bone-marrow-derived macrophage (BMDM; Figure 3—figure supplement 1A–C). Chi3l1 expression was completely abolished in MoMFs from Chi3l1-MKO mice. Considering the partial activity of *Lyz2-Cre* in KCs, we further assessed Chi3l1 expression in KCs isolated from Chi3l1-MKO mice. Only a modest (~40%) reduction in Chi3l1 mRNA and protein levels was observed in KCs, indicating that *Lyz2-Cre*-mediated deletion minimally affects Chi3l1 expression in KCs (Figure 3—figure supplement 1B–C). Chi3l1-MKO and *Chi3l1^fl/fl^* control mice were then fed either a NCD or an HFHC diet. Under both dietary conditions, the two genotypes exhibited comparable phenotypes with respect to body weight gain, hepatic lipid accumulation, metabolic parameters, glucose tolerance, and insulin sensitivity (Figure 3—figure supplement 2A–F). These results indicate that Chi3l1 loss in MoMFs does not substantially impact metabolic regulation.

### ScRNA-seq reveals upregulated glucose metabolism-related transcripts in KCs, correlating with cell death signatures

**Figure 4.**
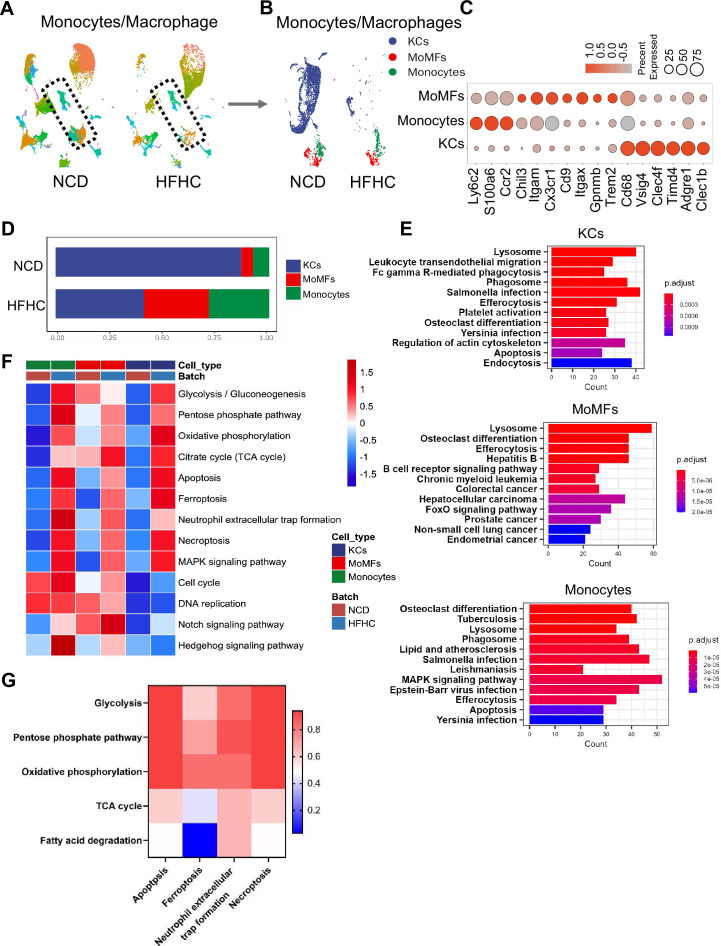
ScRNA-seq reveals upregulated glucose metabolism-related transcripts in kupffer cells (KCs), correlating with cell death signatures. Wild-type (WT) C57BL/6 J mice were fed either a normal chow diet (NCD) or a high-fat, high-cholesterol (HFHC) for 16 weeks. Non-parenchymal cells (NPCs) were isolated and subjected to BD Rhapsody scRNA sequencing. (**A**) Uniform manifold approximation and projection (UMAP) plots illustrate the clustering of NPCs in the livers of mice fed NCD and HFHC. Cell clusters are color-coded, with monocytes/macrophages clusters outlined. (**B**) UMAP plots depict the clustering of monocytes/macrophages in the livers of mice fed NCD and HFHC. Cell clusters are color-coded. (**C**) Dot plot displays the scaled gene expression levels of lineage-specific marker genes in different cell clusters. (**D**) Quantification of each cell cluster is presented. (**E**) Kyoto Encyclopedia of Genes and Genomes (KEGG) analysis reveals the top 12 enriched pathways for upregulated genes when comparing HFHC versus NCD in KCs, monocytes, and monocyte-derived macrophages (MoMFs), respectively. (**F**) Gene set variation analysis (GSVA) shows pathway activity for cell death, glucose metabolism, and cell proliferation in KCs, monocytes, and MoMFs of WT mice fed NCD or HFHC for 16 weeks, respectively. (**G**) The correlation between cell death and glucose metabolism pathways, based on GSVA score, is depicted.

To dissect the distinct metabolic and functional profiles between KCs and MoMFs during MASLD progression, we performed BD Rhapsody single-cell RNA sequencing (scRNA-seq) on liver non-parenchymal cells (NPCs) from mice fed a NCD or an HFHC diet for 16 weeks. After quality control and filtration, we retained 23,312 cells from NCD livers and 6567 cells from HFHC livers for downstream analysis. Using a graph-based clustering approach, we identified 32 distinct cell populations, visualized via uniform manifold approximation and projection (UMAP) (Figure 4A). Monocyte/macrophage subsets were further defined based on lineage-specific markers: Monocytes expressed *Ly6c2, Chil3, S100a6, Ccr2, Itgam*, and *Cx3cr1* but lacked macrophage markers. KCs were marked by *Cd68, Vsig4, Clec4f, TIM4, Adgre1*, and *Clec1b*. MoMFs were negative for KCs markers but positive for macrophage markers such as *Ccr2, Cx3cr1, Cd9, Itgax, Gpnmb, Cd68,* and *Adgre1* (Figure 4B and C; *UMAP in* Figure 4—figure supplement 1; Li et al., 2023; Seidman et al., 2020).

**Figure 4—figure supplement 1.**
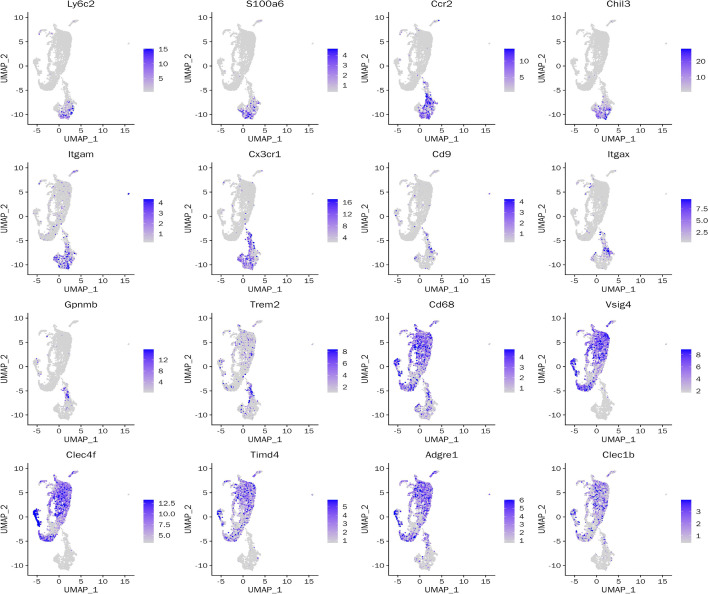
Gene expression levels of lineage-specific marker genes in monocytes/macrophages clusters. Scaled gene expression levels of each lineage-specific marker gene are shown in UMAP plots of monocytes/macrophages clusters. Colors indicate gene expression levels.

Consistent with prior studies (Daemen et al., 2021; Tran et al., 2020), we observed decreased KCs numbers but increased MoMFs and monocytes in HFHC-fed mice compared to NCD controls (Figure 4D). Kyoto Encyclopedia of Genes and Genomes (KEGG) pathway analysis showed that while both cell types exhibited activation of phagocytosis-related pathways (lysosome, phagosome, endocytosis, and efferocytosis), they displayed divergent cell fate patterns (Figure 4E). KCs showed strong cell death signatures, whereas MoMFs maintained proliferative activity without evidence of cell death (Figure 4E). Monocytes showed strong cell death and proliferative activity (Figure 4E). Given the significant role of metabolic regulation in cell fate, (Green et al., 2014) we compared pathways involved in glucose metabolism, cell death, and cell proliferation (Figure 4F). Notably, glucose metabolism pathways were significantly more active in KCs and monocytes compared to MoMFs (Figure 4F). Moreover, the cell proliferation pathway was highly activated in monocytes and consistently activated in MoMFs but not in KCs (Figure 4F). Gene Set Variation Analysis (GSVA)-based correlation analysis revealed a striking association between glucose metabolism and cell death pathways (Figure 4G). These findings demonstrate distinct glucose metabolic activation patterns between KCs and MoMFs, which may underlie their divergent cell fates in MASLD progression.

### Chi3l1 deficiency promotes KCs death during MASLD

**Figure 5.**
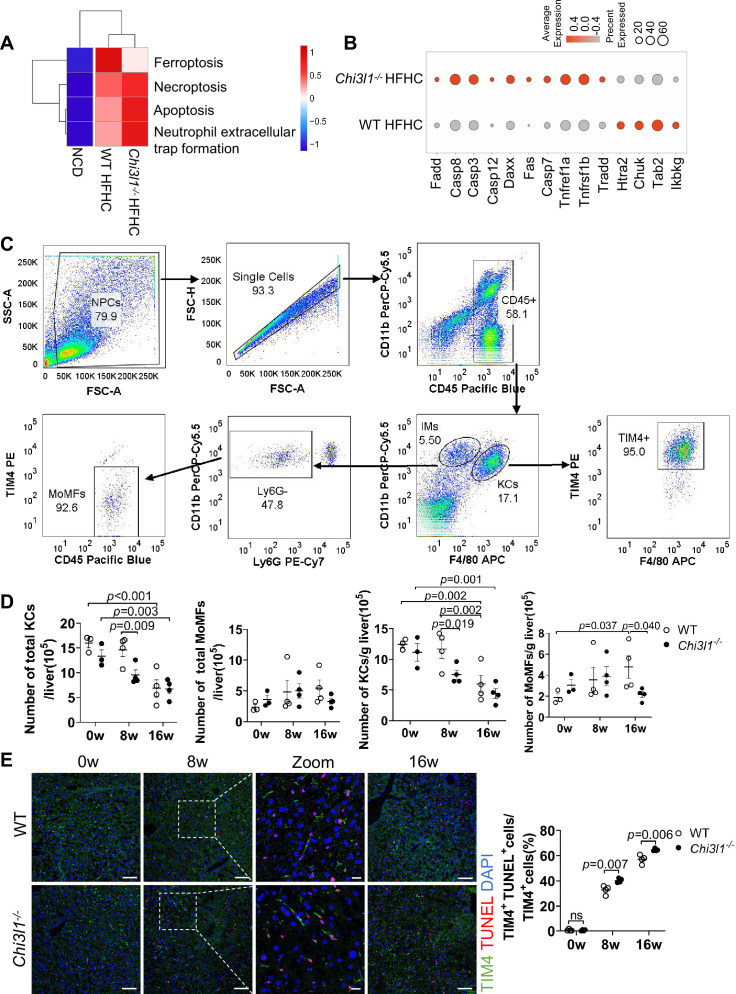
Chi3l1 deficiency promotes Kupffer cells (KCs) death during metabolic dysfunction-associated steatotic liver disease. (**A**) GSVA analysis showed the enrichment of cell death-related pathways in KCs from wild-type (WT) mice fed with either normal chow diet (NCD) or high-fat, high-cholesterol (HFHC) or *Chi3l1^-/-^* mice fed with HFHC. (**B**) Dot plot showing the scaled gene expression levels of apoptosis-related genes and repressor genes in KCs from either WT or *Chi3l1^-/-^* fed with HFHC. (**C**) Strategy used to gate KCs (CD45^+^ F4/80^hi^ CD11b^low^ TIM4^hi^) and monocyte-derived macrophages (MoMFs; CD45^+^ F4/80^low^ CD11b^hi^ Ly6G^-^ TIM4^-^) in the liver by flow cytometry. (**D**) Number of KCs and MoMFs /liver or gram (g) liver were statistically analyzed. n=3–4 mice per group. (**E**) Immunofluorescent staining to detect TIM4 (green), TUNEL (red), and nuclear DAPI (blue) in liver sections. Scale bar = 50 µm and 20 µm (insets). TUNEL^+^ TIM4^+^ cells/TIM4^+^ cells were statistically analyzed. n=4 mice/group. Representative images are shown in C and E. One-way ANOVA was performed in D. Two-tailed, unpaired student t-test was performed in E. p value is as indicated. Figure 5—source data 1.Numerical data of Figure 5D–E.

To investigate the role of Chi3l1 in KCs survival during MASLD, we then performed scRNA-seq on NPCs isolated from *Chi3l1^-/-^* mice fed an HFHC diet for 16 weeks. After quality control, 6813 high-quality cells were retained for analysis. Using established KC markers (Figure 4C), we conducted GSVA to examine metabolic pathways. This revealed enhanced cell death pathways in KCs from HFHC-fed mice, with significantly greater apoptosis signatures in *Chi3l1^-/-^* KCs compared to WT controls (Figure 5A). The increased apoptosis was further supported by upregulation of pro-apoptotic genes in *Chi3l1^-/-^* KCs (Figure 5B).

**Figure 5—figure supplement 1.**
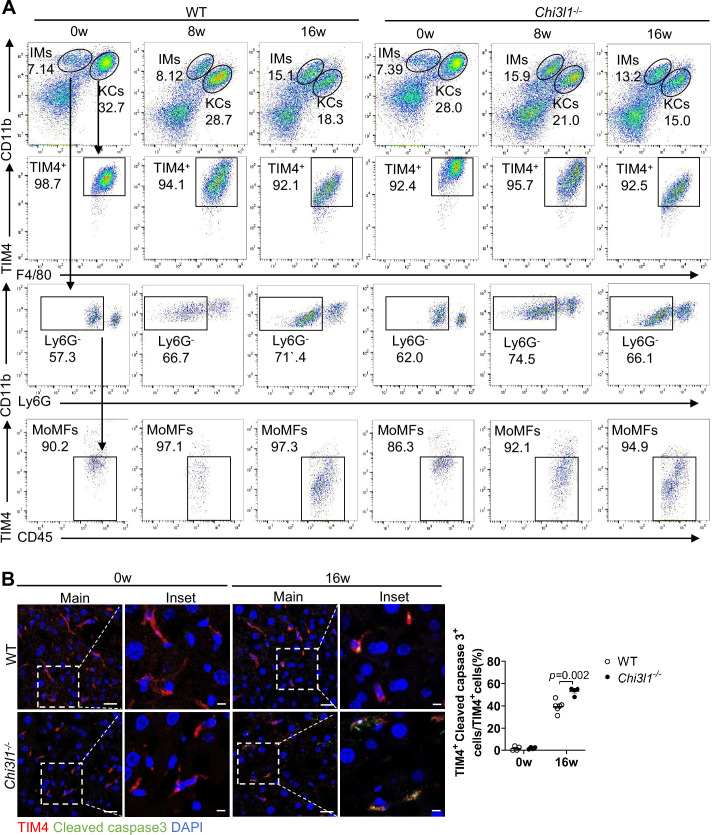
Chi3l1 deficiency promotes Kupffer cells (KCs) death during metabolic dysfunction-associated steatotic liver disease. WT and *Chi3l1^-/-^* mice were fed with a high-fat, high-cholesterol (HFHC) diet for 0, 8, and 16 weeks. (**A**) Flow cytometry analysis of KCs (CD45^+^ F4/80^hi^ CD11b^low^ TIM4^hi^) and MoMFs (CD45^+^ F4/80^low^ CD11b^hi^ Ly6G^-^ TIM4^-^) among non-parenchymal cells (NPCs) between WT and *Chi3l1^-/-^* mice. (**B**) Immunofluorescent staining to detect TIM4 (red), cleaved caspase-3 (green), and nuclear DAPI (blue) in liver sections. Scale bar = 20 μm and 5 μm (insets). Cleaved caspase-3^+^ TIM4^+^ cells/TIM4^+^ cells were statistically analyzed. n=4–6 mice/group. Representative images are shown in A and B. Student t-test was performed in B. p value is as indicated. Figure 5—figure supplement 1—source data 1.Numerical data of Figure 5—figure supplement 1B.

**Figure 5—figure supplement 2.**
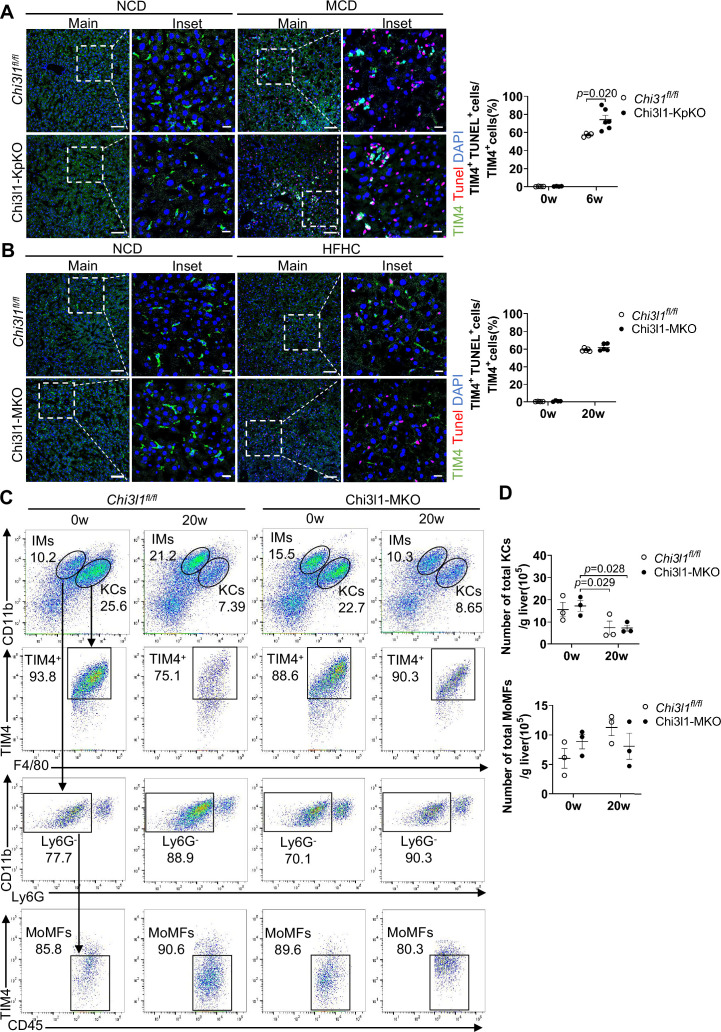
Deficiency of Chi3l1 in Kupffer cells (KCs) but not monocyte-derived macrophages (MoMFs) promotes KCs death during metabolic dysfunction-associated steatotic liver disease. *Chi3l1^fl/fl^* and Chi3l1-KpKO mice were fed with a methionine-choline deficient (MCD) diet for 6 weeks. *Chi3l1^fl/fl^* and Chi3l1-MKO mice were fed with a high-fat, high-cholesterol (HFHC) diet for 20 weeks. (**A**) Immunofluorescent staining to detect TIM4 (green), TUNEL (red), and nuclear DAPI (blue) in liver sections of *Chi3l1^fl/fl^* and Chi3l1-KpKO mice. Scale bar = 50 µm and 20 µm (insets). TUNEL^+^ TIM4^+^ cells/TIM4^+^ cells were statistically analyzed. n=4–6 mice/group. (**B**) Immunofluorescent staining to detect TIM4 (green), TUNEL (red), and nuclear DAPI (blue) in liver sections of *Chi3l1^fl/fl^* and Chi3l1-MKO mice. Scale bar = 50 µm and 20 µm (insets). TUNEL^+^ TIM4^+^ cells/TIM4^+^ cells were statistically analyzed. n=4–5 mice/group. (**C**) Flow cytometry analysis of KCs (CD45^+^ F4/80^hi^ CD11b^low^ TIM4^hi^) and MoMFs (CD45^+^ F4/80^low^ CD11b^hi^ Ly6G^-^ TIM4^-^) among non-parenchymal cells (NPCs) between *Chi3l1^fl/fl^* and Chi3l1-MKO mice. (**D**) Number of KCs or MoMFs/g liver were statistically analyzed. n=3 mice/group. Representative images are shown in A–C. Student t-test was performed in A and B. One-way ANOVA was performed in D. p value is as indicated. Figure 5—figure supplement 2—source data 1.Numerical data of Figure 5—figure supplement 2A-B and D.

We next validated these findings by flow cytometry using the gating strategy shown in Figure 5C. While WT and *Chi3l1^-/-^* mice showed similar KC numbers at baseline, dramatic differences emerged during HFHC feeding. WT KC numbers remained stable at 8 weeks but decreased by 50% at 16 weeks. In contrast, *Chi3l1^-/-^* mice exhibited accelerated KCs loss, with a 30% reduction by 8 weeks progressing to 60% by 16 weeks (Figure 5D, Figure 5—figure supplement 1A). Notably, MoMFs populations remained comparable between groups at early timepoints but showed greater reduction in *Chi3l1^-/-^* mice at 16 weeks (Figure 5D, Figure 5—figure supplement 1A).

Histological analysis further supported these findings. TIM4/TUNEL co-staining revealed no TUNEL^+^ KCs in WT livers at baseline, whereas 40% and 50% of KCs were TUNEL^+^ at 8 and 16 weeks, respectively. In *Chi3l1^-/-^* mice, KC apoptosis was significantly increased at both time points (Figure 5E). Consistent results were obtained with TIM4/cleaved caspase-3 co-staining (Figure 5—figure supplement 1B). We further confirmed these observations in Chi3l1-KpKO mice in both HFHC (He et al., 2025) and MCD diet models. In the MCD model, Chi3l1-KpKO mice exhibited enhanced KCs death compared with *Chi3l1^fl/fl^* controls (Figure 5—figure supplement 2A). To exclude potential effects of myeloid-cell-derived Chi3l1 on KCs survival, we compared KCs death and abundance between *Chi3l1^fl/fl^* and Chi3l1-MKO mice using histological and flow cytometric analyses. Loss of Chi3l1 in MoMFs did not lead to significant KC apoptosis or depletion (Figure 5—figure supplement 2B–D). Together, these results demonstrate that Chi3l1 deficiency promotes KC apoptosis, resulting in premature KC depletion during MASLD progression.

### Molecular interaction between Chi3l1 and glucose

**Figure 6.**
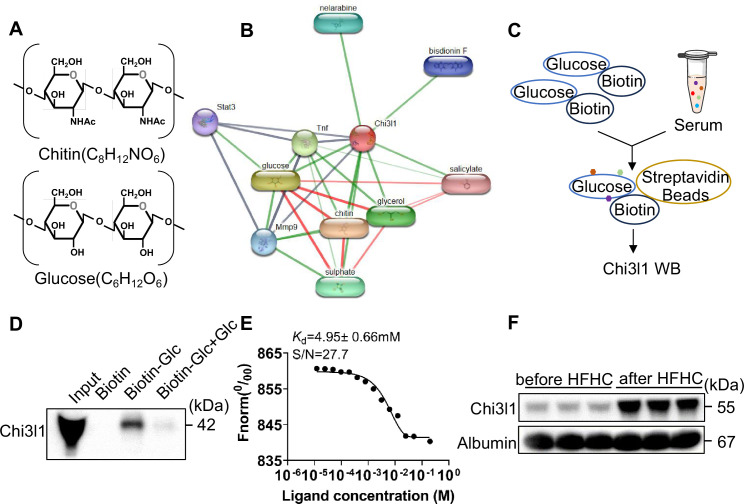
Molecular interaction between Chi3l1 and glucose. (**A**) A comparison of chemical structures between glucose and chitin. (**B**) Prediction of Chi3l1-glucose interaction using STITCH database (http://stitch.embl.de). (**C**) Strategy for pulling down glucose-binding proteins in murine serum. (**D**) Biotin-conjugated glucose was incubated with murine serum from mice fed with high-fat, high-cholesterol (HFHC) diet for 16 weeks. Proteins bound to glucose were precipitated by streptavidin beads. Biotin or biotin-conjugated glucose plus glucose were used as negative controls. Western blot was performed to examine Chi3l1 in the precipitate. (**E**) Microscale thermophoresis assay to detect the interaction between recombinant mouse Chi3l1 (rChi3l1) and glucose. Kd = 4.95 ± 0.66 mM. (**F**) Western blot to detect Chi3l1 expression in murine serum before and after HFHC feeding. n=3 mice/group. Figure 6—source data 1.Numerical data of Figure 6E. Figure 6—source data 2.PDF file containing original western blots for Figure 6D and F, indicating the relevant bands and treatments. Figure 6—source data 3.Original files for western blot analysis displayed in Figure 6D and F.

Our investigation into Chi3l1-mediated KCs survival revealed an unexpected structural relationship: Chi3l1 binds to glucose, which is structurally analogous to chitin, a polysaccharide well known to bind Chi3l1 (Figure 6A). Bioinformatics analysis using the STITCH database further supported this observation, predicting a high probability of direct Chi3l1-glucose interaction (Figure 6B). To experimentally validate this interaction, we performed pull-down assays using biotin-labeled glucose incubated with plasma from HFHC-fed mice. Streptavidin bead isolation followed by anti-Chi3l1 Western blotting demonstrated specific binding between Chi3l1 and biotin-glucose, but not biotin alone (Figure 6C and D). This interaction was competitively inhibited by unlabeled glucose, confirming specificity (Figure 6D). Quantitative analysis using microscale thermophoresis with recombinant mouse Chi3l1 (rChi3l1) yielded a dissociation constant (Kd) of 4.95 mM for the Chi3l1-glucose interaction (Figure 6E). Notably, circulating Chi3l1 levels were significantly elevated in serum from HFHC-fed mice compared to baseline (Figure 6F), suggesting a potential physiological role for this interaction in metabolic regulation. These findings establish Chi3l1 as a novel glucose-binding protein that may participate in glucose homeostasis during MASLD progression.

### Chi3l1 limits glucose uptake and protects hepatic macrophages from cell death

**Figure 7.**
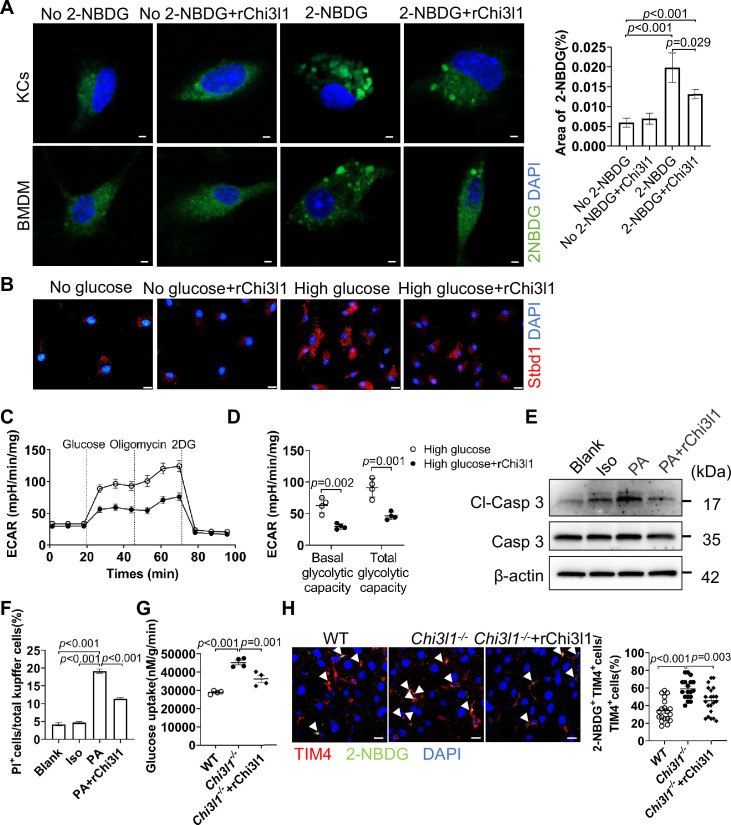
Chi3l1 limits glucose uptake and protects hepatic macrophages from cell death. (**A**) Following 12 hr of glucose starvation, isolated Kupffer cells (KCs) or bone-marrow-derived macrophages (BMDM) were divided into two groups: one treated with no 2-NBDG and the other with 2-NBDG. Within each group, KCs or BMDM were further treated without or with recombinant murine Chi3l1 (rChi3l1) for 6 hr. Glycogen aggregate formation labeled by 2-NBDG (Green) in KCs or BMDM was examined after counterstaining with nuclear DAPI (Blue). Scale bar = 2 μm. Area of 2-NBDG in KCs was quantified. (**B**) Following 12 hr of glucose starvation, BMDM were treated with either no glucose or high glucose (25 mM). Concurrently, BMDM were treated without or with rChi3l1 for 24 hr under each condition. Glycogen aggregate formation in BMDM was detected using immunofluorescence staining for Stbd1 (red) and nuclear DAPI (blue). Scale bar = 10 μm. (**C and D**) BMDM cells were treated without or with rChi3l1 for 24 hr and subjected to Seahorse metabolic analysis to measure the extracellular acidification rate (ECAR). (**E and F**) KCs were treated without (blank) or with either isopropyl alcohol (Iso) or 800 µM palmitic acid (PA) or 100 ng rChi3l1 with 800 µM PA for 24 hr. Western blot was performed to detect cleaved caspase-3 (Cl-Casp3) in E. Calcein/PI staining was quantified to detect cell viability in F. Scale bar = 50 μm. (**G**) Measurement of 2-NBDG (a fluorescent glucose analog) uptake by KCs in vivo. WT and *Chi3l1^-/-^* mice, either untreated or supplemented with rChi3l1, were injected intraperitoneally with 12 mg/kg 2-NBDG. After 45 min, KCs were isolated and glucose uptake assessed by spectrophotometry. (**H**) Representative immunofluorescence images of liver sections stained for TIM4 (red) and 2-NBDG uptake (green) to visualize glucose uptake by KCs in situ. Scale bar = 10 µm (Insets). Quantification is shown as the percentage of TIM4^+^ cells that are also 2-NBDG^+^. Representative images were shown in A, B, and H. One-way ANOVA was performed in A, F, G, and H. Two-tailed, unpaired Student t-test was performed in D. p value is as indicated. Figure 7—source data 1.Numerical data of Figure 7A, C–D and F–H. Figure 7—source data 2.PDF file containing original western blots for Figure 7E, indicating the relevant bands and treatments. Figure 7—source data 3.Original files for western blot analysis displayed in Figure 7E.

To elucidate the functional consequences of Chi3l1-glucose binding, we examined glucose metabolism in hepatic macrophages. Using the fluorescent glucose analog 2-NBDG (Liu et al., 2021), we performed uptake assays in KCs following 12 hr glucose starvation. While glycogen droplet size remained unchanged in untreated KCs regardless of rChi3l1 supplementation (Figure 7A), 2-NBDG exposure significantly increased glycogen accumulation. This effect was markedly suppressed by rChi3l1 co-treatment (Figure 7A), a phenotype replicated in BMDM (Figure 7A). These results demonstrate that Chi3l1 restricts glucose uptake and subsequent glycogen storage.

Further validation using Stbd1 (a glycogen-binding protein Liu et al., 2021) immunofluorescence revealed minimal glycogen foci in glucose-deprived BMDM, with no rChi3l1-dependent differences. High-glucose conditions, however, triggered robust glycogen aggregation, which was significantly attenuated by rChi3l1 (Figure 7B). Concordantly, extracellular acidification rate (ECAR) measurements showed reduced basal and total glycolytic capacity in rChi3l1-treated BMDMs (Figure 7C and D), confirming Chi3l1’s role in limiting glucose metabolism.

To test whether Chi3l1-glucose binding influences cell survival, we employed a palmitic acid (PA)-induced lipotoxicity cell-based model to better mimic the in vivo environment. rChi3l1 supplementation reduced PA-induced cleavage of caspase-3 (Figure 7E) and decreased KCs death (calcein/PI staining, Figure 7F). To validate this mechanism in vivo, we intraperitoneally injected 2-NBDG into WT and *Chi3l1^-/-^* mice, with or without supplementation of rChi3l1, to assess glucose uptake by KCs. *Chi3l1^-/-^* KCs displayed markedly increased 2-NBDG uptake compared with WT controls, whereas rChi3l1 supplementation significantly reduced glucose uptake. These results demonstrate that serum Chi3l1 limits glucose uptake by KCs in vivo (Figure 7G and H). Collectively, these findings demonstrate that Chi3l1 protects KCs from metabolic-stress-induced death by regulating glucose uptake.

## Discussion

**Figure 8.**
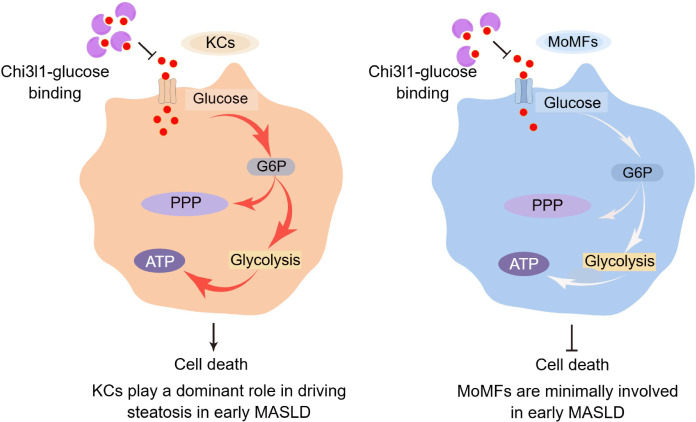
Differential regulation of Kupffer cells (KCs) and monocyte-derived macrophages (MoMFs) fate by Chi3l1-glucose interaction. KCs maintain a high-glucose activation state, while MoMFs exhibit a relatively low-glucose metabolic program. Chi3l1-glucose binding inhibits glucose uptake in KCs, thereby delaying KCs death and alleviating MASLD progression and metabolic dysfunction. In contrast, although Chi3l1-glucose binding similarly inhibits glucose uptake in MoMFs, their low basal glucose metabolism renders them resistant to this metabolic perturbation, resulting in minimal impact on MASLD pathogenesis.

Our findings establish Chi3l1 as a critical metabolic regulator that controls hepatic macrophage fate through a novel glucose-dependent mechanism in MASLD. Using cell-specific knockout models, we uncovered a fundamental dichotomy in Chi3l1 function: selective ablation in KCs dramatically accelerated MASLD progression and metabolic dysfunction, whereas deletion in MoMFs produced minimal metabolic effects. Single-cell transcriptomics revealed the molecular basis for this cell-type specificity – KCs exhibit a glucose-hungry metabolic phenotype that renders them uniquely dependent on Chi3l1-mediated regulation, while MoMFs maintain a relatively glucose-independent metabolic program. At the mechanistic level, we demonstrate that Chi3l1 functions as a physiological glucose sensor, directly binding extracellular glucose to limit its cellular uptake. This interaction establishes a crucial metabolic safeguard that specifically protects glucose-dependent KCs from lethal metabolic stress while sparing glucose-independent MoMFs. Through this precise modulation of glucose availability, Chi3l1 maintains metabolic homeostasis and preserves KCs populations during chronic dietary challenge (Figure 8).

Analysis of publicly available scRNA-seq datasets, including those from the Liver Atlas and prior studies (Daemen et al., 2021; Remmerie et al., 2020; Seidman et al., 2020), indicates that Chi3l1 transcripts are mainly detected in neutrophils. In contrast, our immunofluorescence data show that Chi3l1 protein is predominantly localized in KCs under normal conditions and in both KCs and MoMFs during MASLD progression. This discrepancy likely reflects differences in transcript versus protein abundance and detection sensitivity. While scRNA-seq captures relative mRNA levels per cell, tissue-based staining reflects both expression and cell prevalence, highlighting macrophages as a major contributor to total hepatic Chi3l1 protein. Moreover, environmental factors such as diet, microbiota, or disease stage may influence Chi3l1 expression patterns across immune cell types.

Our study reveals fundamental differences in metabolic requirements between hepatic macrophage subsets that provide new insights into MASLD pathogenesis. We demonstrate that KCs and MoMFs play stage-specific roles in disease progression, with KCs serving as critical regulators of early metabolic homeostasis while MoMFs appear more involved in later inflammatory phases. This temporal specialization explains the striking dichotomy observed in our genetic models-KCs-specific Chi3l1 deletion dramatically exacerbated metabolic dysfunction, whereas MoMFs deletion showed minimal effects. The heightened glucose metabolism of KCs during MASLD renders them uniquely vulnerable to dietary stress. Chi3l1 serves as a crucial metabolic buffer in this context, directly protecting KCs through glucose modulation as evidenced by reduced glycogen accumulation and attenuated glycolytic flux. Our findings using the HFHC model complement previous findings in fibrogenic CDAA-HFAT models (Kim et al., 2023) or MCD/CCL_4_ models (Higashiyama et al., 2019) or human livers (Nishimura et al., 2021), collectively suggesting Chi3l1 may have dual roles in MASLD – maintaining metabolic balance through KCs in early disease while potentially influencing fibrogenesis via MoMFs in advanced stages. The accelerated KCs death in knockout models provides direct experimental evidence linking macrophage survival to metabolic outcomes, resolving key questions about MASLD progression mechanisms.

The structural characteristics of Chi3l1 have been extensively studied. Chi3l1 forms a homodimer, with each subunit containing a catalytic domain and a carbohydrate-binding domain. While the catalytic domain retains structural similarity to chitinases, it lacks enzymatic activity (Fusetti et al., 2003; Houston et al., 2003), and the carbohydrate-binding domain mediates interactions with carbohydrate ligands (Fusetti et al., 2003). While chitin-binding domains are traditionally known to interact with complex polysaccharides, our findings reveal that Chi3l1 (YKL-40), a mammalian chitinase-like protein, specifically binds to glucose—a simple monosaccharide. This represents a fundamental departure from canonical binding to insoluble polymers such as chitin and suggests a previously unrecognized role for Chi3l1 in monosaccharide recognition, potentially linking it to glucose metabolism and energy sensing. Furthermore, we observed that Chi3l1 protein levels increased in the serum of mice fed an HFHC diet for 16 weeks (Figure 6F) but plateaued with prolonged feeding (24 weeks; data not shown), suggesting an adaptive regulatory limit. Together, these findings indicate that Chi3l1 possesses glucose-binding capacity that may be functionally relevant but limited in vivo.

Our findings carry important translational potential for MASLD treatment. The discovery of Chi3l1’s glucose-sensing function in KCs suggests two complementary therapeutic strategies: first, developing Chi3l1-based interventions to preserve KC populations during early metabolic dysfunction; second, creating cell-type-specific approaches that selectively modulate glucose metabolism in KCs while sparing MoMFs. Importantly, although access to early-stage human liver tissue is limited due to the asymptomatic nature of the disease, multiple human studies have consistently reported elevated Chi3l1 levels in steatotic and fibrotic liver disease (Higashiyama et al., 2019; Huang et al., 2022; Liu et al., 2025), underscoring the clinical relevance of our mechanistic findings. Building on this evidence, the structural mapping of Chi3l1’s glucose-binding domain now enables rational design of small-molecule mimetics or biologics to therapeutically enhance this protective pathway. Besides, several key questions emerge for future research to advance these therapeutic possibilities: (1) How glucose levels are coordinated with other death inducers such as lipid toxicity; (2) Whether competing carbohydrate ligands modulate Chi3l1’s glucose-sensing capacity in different metabolic states; (3) Functional validation in primary human macrophages or human liver tissues would further strengthen the translational significance of this work. Addressing these questions will be crucial for translating our mechanistic insights into targeted therapies that account for the complex metabolic specialization of hepatic macrophage subsets.

Our findings reveal a novel metabolic checkpoint in which Chi3l1 selectively sustains KCs populations by modulating glucose metabolism, offering key insights into MASLD pathogenesis. The study highlights the therapeutic potential of targeting Chi3l1-glucose interactions to preserve protective KCs and curb MASLD progression. Future research should explore whether Chi3l1 supplementation or pharmacological modulation can rescue KCs viability, as well as investigate whether this mechanism extends to other macrophage-driven metabolic disorders, such as MASH or diabetes. By identifying cell-type-specific metabolic vulnerabilities, this work paves the way for precision therapies that selectively manipulate macrophage subsets to treat liver disease.

## Materials and methods

### Animal experiments and procedures

#### Animals

*Chi3l1^-/-^* (strain no. T014402), *Chi3l1^flox//flox^* (strain no. T013652), *Lyz2-cre* (strain no. T003822), and *Clec4f-cre* (strain no. T036801) with a C57BL/6 J background were purchased from GemPharmatech. Rosa26^LSL-tdTomato/+^ mice (strain no. C001181) were purchased from Cyagen. Accordingly, C57BL/6 J mice (strain no. N000013) were used as WT mice. To generate Chi3l1-KpKO mice, *Chi3l1^flox//flox^* mice were crossed with *Clec4f-cre* mice. To generate Clec4f^CreERT2/+^; Rosa26^LSL-tdTomato/+^ mice, Rosa26^LSL-tdTomato/+^ mice were crossed with *Clec4f-cre* mice. To generate Chi3l1-MKO mice, *Chi3l1^flox//flox^* mice were crossed with *Lyz2-cre* mice. Male mice have been the choice in the vast majority of the studies of MASLD reported in the literature (Fu et al., 2024; Jeelani et al., 2024). Therefore, we used male mice in the majority of the experiments presented. All mouse colonies were maintained at the Animal Core Facility of Yunnan University. The animal studies were approved by the Yunnan University Institutional Animal Care and Use Committee (IACUC, Approval No. YNU20220314).

#### Construction of MASLD/MASH mouse model

Mice were provided an HFHC diet (Research Diet, d12108c, 40 kcal% fat and 1.25% cholesterol) for 16 weeks or an MCD diet (Research Diet, A02082002BR) for 6 weeks. Throughout the feeding period, the body weight and food consumption of the mice were observed and recorded weekly. Once the dietary intervention was completed, the mice were euthanized. Liver and murine serum samples were collected for further analysis. Alanine aminotransferase (ALT) and aspartate aminotransferase (AST) levels in the serum, as well as cholesterol (TC) and triglyceride (TG) levels in both serum and liver tissues, were quantified using commercially available kits (Nanjing Jiancheng Bioengineering Institute).

#### Intraperitoneal glucose tolerance test (IGTT) and intraperitoneal insulin tolerance test (ITT)

For the IGTT, the mice were fasted for approximately 13–14 hr while having access to water. They were then intraperitoneally injected with saline containing 20% glucose (1 g/kg of body weight, Cat# 10057153461926). For the ITT, the mice were fasted for approximately 5 hr with access to water. They were then intraperitoneally injected with insulin (0.1 U/mL, 0.75 U/kg of body weight, Cat# 10090506507183). During both tests, the blood glucose levels were measured at 0, 30, 60, 90, and 120 min using a blood glucose meter (Roche ACCU-CHEK Performa). Care was taken to collect blood from the tip of the tail by using gentle movements to avoid stress-induced hyperglycemia. After the tests, the mice were placed in a clean cage with food and water available and monitored for 1 hr. To ensure accurate and reliable results, we conducted any subsequent ITTs at least 1 week after the previous test to avoid any interference.

#### Genotyping

Sample preparation and procedure were conducted as previously described (Chen et al., 2024). Primer sequences are provided in Supplementary file 1.

### Isolation of NPCs and KCs

Hepatic NPCs were isolated as previously described (Shan et al., 2021). In brief, the abdominal cavity of mice was promptly opened, and the hepatic portal vein was located after anesthesia. A soft needle was inserted for perfusion with Perfusion buffer (100 mL 1×HBSS, 200 µL 0.5 mol/L EGTA) for 3–5 min, while the inferior vena cava was cut to clear the liver. Subsequently, Digestion buffer (60 mL 1×HBSS, 120 µL 2 M MgSO_4_, 60 µL 1.25 M CaCl_2_•H_2_O, 0.015 g Collagenase I) was injected at a consistent rate for approximately 15 min. Following perfusion, the liver was delicately transferred into a pre-cooled petri dish with PBS, cut into 2–3 mm fragments, and incubated at 37°C for 15 min in digestion buffer. The liver tissue fluid was then filtered through a 70-µm cell filter and centrifuged at 50 × *g* for 5 min (ac/brake = 0) at 4°C to collect the supernatant. This supernatant underwent centrifugation at 450 × *g* for 5 min (ac/brake = 5) at 4°C to obtain the NPCs population. The NPCs were suspended in a 15 mL centrifuge tube with 4 mL 20% Optiprep solution, followed by centrifugation at 3000 rpm for 17 min (ac/brake = 0) at 4°C. After centrifugation, the cells at the boundary between the Optiprep solution and 1×HBSS buffer were collected into a new tube, supplemented with 1×HBSS buffer, and centrifuged for 5 min at 450 × *g* (ac/brake = 5). RBCs were lysed with 1 mL ACK buffer (150 mM NH4Cl, 10 mM KHCO3, 0.1 mM Na2EDTA, pH = 7.2–7.4), neutralized with 1×HBSS buffer, and centrifuged. The cells were resuspended in cell medium and placed in a 3.5 cm petri dish for 10 min to obtain KCs by washing out un-adherent cells with PBS. The purity and viability of isolated KCs reached 90% and 80%, respectively, which was confirmed by staining with anti-Timd4 antibodies or trypan blue.

### Preparation of bone-marrow-derived macrophages

To obtain BMDMs, femur and tibia bone marrow from healthy male *C57BL6/J* was extracted, resuspended in DMEM/F12 medium (VivaCell, Cat# C3113-0500) containing 10% FBS (VivaCell, Cat# C04001-500) and 20% L929 conditioned medium, seeded in culture dishes, and cultured at 37 °C with 5% CO_2_ in a humidified atmosphere for 7 days. Fresh medium was added on day 4. The cells were maintained in a standard 37 °C in 5% CO_2_ incubator. L929 was a gift from Dr. Guangxun Meng (Hainan Academy of Medical Sciences). Cell identity has been authenticated by the STR profiling. Mycoplasma contamination was tested negative by PCR.

### Flow cytometry

The NPCs were resuspended in fluorescence-activated cell sorting (FACS) buffer, consisting of PBS supplemented with 2% bovine serum albumin. 1 µL of anti-CD16/CD32 antibody (Invitrogen, Cat# 14-0161-86) was added to the cell suspension, and the mixture was incubated at 4°C for 10 min to block any nonspecific binding. After blocking, the Mouse NPCs were labeled with monoclonal antibodies conjugated with fluorescent dyes. This labeling process was carried out at 4°C for 30 min. The labeled cells were washed thrice with cold FACS buffer. The antibodies used for labeling were as follows: anti-mouse F4/80 (APC) (Invitrogen, Cat# 17-4801-82), anti-CD45 (eFluor450; Invitrogen, Cat# 48-0451-82), anti-mouse Tim-4 (PE) (Invitrogen, Cat# 12-5866-82), and anti-mouse CD11b (PerCP/Cyanine5.5; BioLegend, Cat# 101228). All the primary antibodies were used at a dilution of 1:100. The samples were analyzed by flow cytometry using an LSR Fortessa Cell Analyzer (BD Biosciences). The data obtained were further analyzed using FlowJo version 10.0.

### Histology and immunofluorescence

#### H&E staining

Tissues were fixed with buffered 10% paraformaldehyde (Sangon Biotech, Cat# A500684-0500) overnight at 4 °C and embedded in paraffin. Ultra-thin tissue slices (5 μm) were prepared and deparaffinized. H&E staining was performed on the tissue sections, and the slides were examined under a microscope (Olympus, BP80).

#### Immunofluorescence on frozen section

Immunofluorescence staining was conducted on frozen sections of fresh liver tissues. Initially, the tissues were fixed using 2% paraformaldehyde for 1 hr and subsequently dehydrated overnight in a 30% sucrose solution. The following day, tissue embedding was performed using OCT (SAKURA, Cat# 4583), after which ultra-thin sections of 5 μm were sliced. Permeabilization was achieved using 0.02% Triton X-100 for 10 min at room temperature, followed by blocking with 5% normal goat serum (VivaCell, Cat# C2530-0100). Primary antibodies against mouse F4/80 (BioLegend, Cat# 123140, 1:300, Alexa Fluor 594), TIM4 (BioLegend, Cat# 130008, 1:300, Alexa Fluor 647), and Chi3l1 (Abcam, ab180569, 1:400) were then applied. Secondary antibodies (Affinipure Goat Anti-Rabbit 488-conjugated, Jackson ImmunoResearch, 111-545-003, 1:1000 and Goat Anti-Mouse 594-conjugated, Jackson ImmunoResearch, 115-585-003, 1:1000) were used accordingly, with F4/80 and TIM4 antibodies being self-fluorescent and thus not requiring secondary labeling. After washing, the slides were mounted using antifade medium, and nuclei were stained with DAPI. Additionally, TUNEL staining was performed on separate frozen sections of liver tissues. Following fixation with 4% paraformaldehyde and treatment with Proteinase K (20 μg/mL in PBS, BBI, Cat# B600169-0002), the sections were permeabilized with Triton X-100 and blocked with normal goat serum. Subsequently, the sections were incubated with an anti-mouse TIM4 (BioLegend, Cat# 130008, 1:300, Alexa Fluor 647), followed by TUNEL staining (Servicebio, Cat# G1502-100T) according to the manufacturer’s instructions. Nuclei were counterstained with DAPI, and images were captured using a confocal laser scanning microscope (ZEISS, LSM900).

#### Oil Red O staining

Oil Red O staining was conducted on unfixed frozen sections embedded directly in OCT. Frozen sections of the liver were cut at a thickness of 10 μm. After rinsing with water, the sections were immersed in 60% isopropanol for 2 min. Subsequently, the sections were stained with an Oil Red O staining solution (Solarbio, Cat# IO1720) at 37 °C for 10–15 min. Following staining, the sections were immediately placed in 60% isopropanol and washed three to five times to eliminate excess dye solution. Nuclei were counterstained with a hematoxylin staining solution. After rinsing with distilled water, the sections were sealed with glycerol gelatin (Solarbio, Cat# S2150).

#### Sirius red staining

Tissues were fixed with buffered 10% paraformaldehyde (Sangon Biotech, Cat# A500684-0500) overnight at 4 °C and embedded in paraffin. Ultra-thin tissue slices (5 μm) were prepared and deparaffinized. Sirius red staining (Solarbio, Cat# G1472) was performed according to the manufacturer’s instructions, and the slides were examined under a microscope (Olympus, BP80).

#### Immunofluorescent staining on BMDM or KCs

Cells were seeded on coverslips in 12-well plates and treated with or without recombinant murine Chi3l1 (rChi3l1, 100 ng/mL, SB, Cat# 50929-M08H; diluted in PBS) under no glucose or high glucose (25 mM, VivaCell, Cat# C3113-0500) for 24 hr. The cell slides were washed with cold PBS and fixed with 4% paraformaldehyde in PBS for 10 min at room temperature. The cells were permeabilized with 0.02% Triton X-100 for 10 min at room temperature. After blocking with 5% normal goat serum, cells were incubated with primary antibodies anti-STBD1 (Proteintech, Cat# 11842–1-AP, 1:300) overnight at 4°C to label glycogen in the cells. On the second day, after washing with 0.05% PBST, cells were incubated with 594-conjugated Goat Anti-Mouse IgG (H+L) (Jackson ImmunoResearch, 115-585-003, 1:1000) for 1 hr at room temperature. After rinsing with 0.05% PBST, cells were counterstained with DAPI (Beyotime, C1006) and mounted onto slides. Images were captured with an Olympus BP80 microscope. The immunofluorescent staining on KCs follows a similar protocol to that used for BMDM. Primary antibodies against TIM4 (BioLegend, Cat# 130008, 1:300, Alexa Fluor 647) are employed to label KCs.

#### Utilize 2-NBDG to track glucose uptake

To assess glucose uptake, 2-NBDG, a fluorescent glucose derivative, was utilized as a substitute for glucose in detecting glycogen formation within live cells (Liu et al., 2021). Following 12 hr of glucose deprivation, cells were treated with 20 μM 2-NBDG (Invitrogen, Cat# N13195; diluted in PBS) for 6 hr. Post-treatment, cells were fixed with paraformaldehyde and counterstained with DAPI before being mounted onto slides using coverslips. Imaging of the samples was performed using a confocal laser scanning microscope (ZEISS, LSM900), facilitating visualization and analysis of glucose uptake dynamics.

#### Calcein/PI staining

Live cell staining using Calcein/PI was conducted following the manufacturer’s instructions from a commercially available kit (Beyotime, Cat# C2015M). Isolated KCs were seeded in 12-well plates and subjected to various treatments. In one set of experiments, cells were treated with either isopropyl alcohol (1 μL, Sangon Biotech, Cat# A507048-0500) as a control or palmitic acid (800 mM in 1 μL, Sigma, Cat# P0500), or 100 ng/mL rChi3l1 with palmitic acid, all for 24 hr.

### Western blot analysis

The sample preparation and procedure were conducted as previously described (Chen et al., 2024). The following antibodies were used: anti-Caspase-3 (Cell Signaling Technology, Cat# 9662 S, 1:1000), anti-Cleaved Caspase-3 (Cell Signaling Technology, Cat# 9664 S, 1:1000), anti-β-actin (Proteintech, Cat# 66009–1-lg, 1:1000), anti-Chi3l1 (Proteintech, Cat# 21829–1-AP, 1:1000), anti-Albumin (Proteintech, Cat# 21829–1-AP, 1:1000), anti-GAPDH (CWBIO, Cat# cw0100M, 1:1500), anti-α-SMA (Invitrogen, Cat# 50-9760-82, 1:1000). Peroxidase-conjugated Affinipure Goat Anti-Mouse IgG (H+L) (Jackson ImmunoResearch, 115-035-003, 1:2000), Peroxidase-conjugated Affinipure Goat Anti-Rabbit IgG (H+L) (Jackson ImmunoResearch, 111-035-003, 1:2000), and Peroxidase-conjugated Affinipure Goat Anti-Rat IgG (H+L) (Jackson ImmunoResearch, 112-035-003, 1:2000) were used for secondary antibody incubation.

### Microscale thermophoresis (MST) assay

The dissociation constant (Kd) for the interaction between Chi3l1 and glucose was determined using a Microscale Thermophoresis (MST) instrument (NanoTemper Technologies). Mouse Chi3l1 protein was labeled with a His-Tag Labeling Kit RED-tris-NTA 2nd Generation (NanoTemper Technologies, Cat# MO-L018) following the manufacturer’s instructions. Briefly, 90 μL of dye solution (final concentration 100 nM) was mixed with 90 μL of purified Chi3l1 protein and incubated at room temperature for 30 min. The mixture was then centrifuged at 15,000 × *g* for 10 min at 4 °C, and the supernatant was collected for subsequent binding assays. Serial dilutions of glucose were prepared in 1×PBS T buffer (1×PBS containing 0.05% Tween-20, pH 7.4) to generate 16 concentration gradients. Equal volumes of labeled Chi3l1 protein were added to the diluted glucose solutions and incubated at room temperature in the dark for 30 min. The samples were loaded into premium capillaries (NanoTemper Technologies, Cat# MO-K022), and MST measurements were performed at 25 °C using medium MST power and 100% excitation power. Data were collected and analyzed using MO.Affinity Analysis Software (version 2.3) to calculate the dissociation constant (Kd).

### Biotin-glucose pull-down assay

Biotin-glucose chemical synthesis and biotin-glucose pull-down assays were conducted according to the established protocols (Chen et al., 2023). In summary, Magic Dynabeads M-280 Streptavidin (Invitrogen, Cat# 11205D) was pre-incubated with either free biotin or biotin-labeled glucose at 4 °C for 1 hr. Next, the beads were incubated with serum from mice fed HFHC diet for 16 weeks overnight at 4 °C. Afterward, the beads were washed six times with wash buffer and subjected to immunoblotting.

### Preparation of single-cell RNA-seq library and data analysis

After isolating single NPCs following the procedure described above, the cells were captured using the BD Rhapsody system (BD Biosciences, Cat# 633731). Subsequently, the captured single cells underwent whole transcriptome amplification following the BD Rhapsody workflow (BD Biosciences, Cat# 633733). Transcriptome libraries were then prepared from the amplified cDNA using the library preparation protocols provided by BD Rhapsody (BD Biosciences, Cat# 633801). Quality control checks (Revvity) were conducted on the prepared libraries to ensure proper amplification and library construction. Finally, the libraries were sequenced using BD platforms.

The scRNA-seq data were processed and analyzed using R (version 4.0.5) and the R/Seurat package (version 4.2.3). To ensure data quality, low-quality cells, empty droplets, and cells from multiplexed captures were excluded based on the distribution of unique genes detected in each cell. Additionally, genes expressed in fewer than three cells in a sample were excluded. Cells with fewer than 200 genes or more than 4000 genes were excluded, and cells with a mitochondrial gene fraction exceeding 25% were removed. After quality control, datasets from different batches were normalized using SCTransform. For the integrated data, reciprocal PCA-based integration was performed using the FindIntegrationAnchors and IntegrateData functions. Subsequent clustering was conducted using the FindClusters function with resolution values of 0.8 or 1.0, depending on the dataset. Normalization accounted for total unique molecular identifier (UMI) and mitochondrial gene content, and analysis was performed using the 4000 most variable genes. Visualization of the clustered data was achieved using Uniform Manifold Approximation and Projection (UMAP).

To identify cluster biomarkers, we utilized the Find Markers function and Find All Markers function in Seurat, setting a minimum percentage of expression (min.pct) of 0.1 and a log-fold change threshold (logfc.threshold) of 0.25. Cell types were annotated using marker genes with CellMaker 2.0. Subsequently, remaining cells associated with KCs and monocytes were extracted and reclustered. We conducted re-normalization, RPCA-based integration, and cluster stability analysis, determining an optimal resolution of 0.5 or 0.3, depending on the dataset. Cell annotations were based on known cell-lineage-specific marker genes (Li et al., 2023). For differential gene and pathway analysis, differential expression analysis between groups such as NCD and HFHC or NCD, WT HFHC, and *Chi3l1^-/-^* HFHC was performed using the Find Markers function. Pathway analysis utilized the KEGG, while gene ontology (GO) analysis was conducted using the cluster Profiler package to explore functional categories associated with differentially expressed genes. The most highly enriched pathways were identified using the enrich GO and enrich KEGG functions from the cluster Profiler package. Additionally, GSVA was employed to explore associations between cell death and other pathways, utilizing the gsva function from the GSVA R package. Pathways were visualized using the pheatmap R package.

### Extracellular acidification rate (ECAR) measurement

The ECAR measurements were conducted according to the manufacturer’s instructions (Agilent, Cat# 103020–100). Briefly, mature BMDM were cultured in a Seahorse XF24 cell culture plate. After 12 hr of culture, the DMEM culture medium was pre-treated for 24 hr. 1 hr before the analysis, the culture medium was changed to the corresponding XF basal medium (Agilent, Cat#103334–100) supplemented with glutamine (the final concentration was 2 mmol/L, Agilent, Cat#103579–100), and the culture plates were incubated at 37 °C without CO_2_. Compounds were added in the following order: 10 mM glucose, 1.0 mM oligomycin, and 50 mM 2-deoxyglucose. Measurements were conducted using a Seahorse XF24 analyzer (Agilent Technologies). After completing the SeahorseXF glycolytic stress test, basic glycolytic ability and total glycolytic ability were calculated based on the generated report.

### RNA extraction and quantitative real-time PCR

Total RNA was extracted from cultured cells or ground tissues using TRIzol reagent (Invitrogen, Cat# 15596018) according to the manufacturer’s instructions. Briefly, lysates were centrifuged at 12,000 × *g* for 10 min at 4 °C, and the supernatant was transferred to a new tube. Chloroform (200 μL) was added, mixed thoroughly, and incubated at room temperature for 10 min, followed by centrifugation. The aqueous phase was collected, mixed with an equal volume of isopropanol, and incubated for 10 min at 4 °C to precipitate RNA. After centrifugation, the pellet was washed twice with 75% ethanol, air-dried for 10 min at room temperature, and dissolved in RNase-free water. RNA concentration and purity were assessed using a NanoDrop spectrophotometer (Thermo Fisher Scientific). Complementary DNA (cDNA) was synthesized from total RNA using the PrimeScript RT Reagent Kit (Takara, Cat# 6210B) following the manufacturer’s instructions. Quantitative real-time PCR (qPCR) was performed using SYBR Green Master Mix (Thermo Fisher, Cat# A25742) in triplicate on a QuantStudio 1 Real-Time PCR System (Life Technologies, Grand Island, NY, USA). Relative gene expression was calculated using the ΔΔCt method, and primer sequences are listed in Supplementary file 2.

### In vivo glucose uptake assay

In vivo glucose uptake was assessed as previously described (Shi et al., 2025). Mice were fasted for 16 hr and subsequently refed. At 45 min after refeeding, mice were intraperitoneally injected with 2-NBDG (100 μL, 12 mg/kg body weight). Mice were euthanized 10 min post-injection, and liver tissues were collected for analysis. For quantification of total glucose uptake, weighed portions of liver tissue were homogenized in cell lysis buffer (150 mM NaCl, 50 mM HEPES, pH 7.4, 1% Triton X-100). After centrifugation, 2-NBDG fluorescence intensity in the supernatant was measured using a microplate reader (excitation/emission: 465/540 nm). For histological analysis, liver samples were dehydrated overnight in 30% sucrose, embedded in OCT compound (SAKURA, Cat# 4583), and sectioned at 5 μm thickness. Sections were permeabilized with 0.02% Triton X-100 for 10 min at room temperature and blocked with 5% normal goat serum (VivaCell, Cat# C2530-0100). KCs were identified using an anti-TIM4 antibody (BioLegend, Cat# 130008; 1:300, Alexa Fluor 647). Nuclei were counterstained with DAPI, and images were acquired using a confocal laser scanning microscope (ZEISS LSM900).

### Data analysis from Gene Expression Omnibus (GEO) database

The mRNA expression of Chi3l1 was analyzed using liver tissue sequencing data obtained from the NCBI Gene Expression Omnibus database (https://www.ncbi.nlm.nih.gov/geo). The dataset consisted of global RNA sequencing results from 51 patients with MASLD and 47 patients with MASH. The accession number for this dataset was GSE167523. Additionally, RNA sequencing results from the same database were obtained for liver tissues from 5 patients without MASLD, 15 patients with MASLD, and 10 patients with MASH (accession number: GSE207310). Liver tissue sequencing data from 78 patients with MASLD were obtained from a database. This included 20 patients without MASH and 58 patients with MASH (accession number: GSE130970).

### Statistical analysis

Data are presented as mean ± SEM in all graph figures. Statistical analyses were conducted using the SPSS Statistics software (Version 22). To compare the two groups, an unpaired two-tailed Student's t-test was used. One-way analysis of variance (ANOVA) was performed for comparisons involving three or more groups. For patients with MASLD liver, the samples were tested using the Mann-Whitney test. Statistical significance was set at p<0.05 and p value is indicated. All cell culture results represent at least three independent experiments.

## Data Availability

All data generated or analyzed during this study are included in the manuscript and supporting files; source data files have been provided. All reagents developed in this study are available upon reasonable request. The raw single-cell RNA sequencing data have been deposited in the GEO database (accession number: GSE275551). The following dataset was generated: StankeviciusV SuziedelisK 2025Gene expression profile of two colorectal cancer HT29 and DLD-1 cell lines cultivated in 2-dimensional or 3-dimensional cell culture enriched with laminin rich extracellular matrix proteinsNCBI Gene Expression OmnibusGSE75551

## Additional files

Supplementary file 1. PCR primer sequences for genotyping.

Supplementary file 2. qPCR primers.

MDAR checklist

## Appendix 1

**Appendix 1—key resources table:**

Reagent type (species) or resource	Designation	Source or reference	Identifiers	Additional information
Antibody	Mouse monoclonal anti-TIM4 (Alexa Fluor 647) antibody	Biolegend	Cat# 130008; RRID:AB_2271648	IF (1:300)
Antibody	Mouse monoclonal anti-β-actin antibody	Proteintech	Cat# 66009–1-lg; RRID:AB_2687938	WB (1:1000)
Antibody	Cleaved caspase-3 rabbit antibody	Cell Signalling Technology	Cat# 9664 S; RRID:AB_2070042	IF (1:300), WB (1:1000)
Antibody	Caspase-3 rabbit antibody	Cell Signalling Technology	Cat# 9662 S; RRID:AB_331439	WB (1:1000)
Antibody	Rabbit polyclonal anti-YKL-40/CHI3L1 antibody	Abcam	Cat# ab180569; RRID:AB_2891040	IF (1:400)
Antibody	Anti-alpha-smooth muscle actin	Invitrogen	Cat# 50-9760-82; RRID:AB_2574362	WB (1:1000)
Antibody	GAPDH monoclonal antibody	Proteintech	Cat# 60004–1; RRID:AB_2107436	WB (1:1500)
Antibody	Albumin antibody	Cell Signaling	Cat# 4929 s; RRID:AB_2225785	WB (1:1000)
Antibody	Alexa Fluor 594 anti-mouse F4/80	BioLegend	Cat# 123140; RRID:AB_2563241	IF (1:300)
Antibody	Anti-STBD1 rabbit	Proteintech	Cat# 11842–1-AP; RRID:AB_2197523	IF (1:300)
Antibody	Rat monoclonal anti-F4/80 (APC) antibody	Invitrogen	Cat# 17-4801-82; RRID:AB_2784648	Flow cytometry (1:100)
Antibody	Rat monoclonal anti-CD45 (eFluor450) antibody	Invitrogen	Cat# 48-0451-82; RRID:AB_1518806	Flow cytometry (1:100)
Antibody	Rat monoclonal anti-TIM-4 (PE) antibody	Invitrogen	Cat# 12-5866-82; RRID:AB_1257163	Flow cytometry (1:100)
Antibody	Rat monoclonal anti-CD16/CD32	Invitrogen	Cat# 14-0161-86; RRID:AB_467135	Flow cytometry (1:100)
Antibody	Rat monoclonal anti-CD11b (PerCP/Cyanine5.5) antibody	Biolegend	Cat# 101228; RRID:AB_893232	Flow cytometry (1:100)
Antibody	Ly-6G monoclonal antibody (1A8-Ly6g) PE-Cyanine7	Invitrogen	Cat# 25-9668-82; RRID:AB_2811793	Flow cytometry (1:100)
Antibody	Peroxidase-conjugated affinipure goat anti-rabbit IgG (H+L)	Jackson	Cat# 111-035-003; RRID:AB_2313567	WB (1:2000)
Antibody	Peroxidase-conjugated affinipure goat anti-mouse IgG (H+L)	Jackson	Cat# 115-035-003; RRID:AB_10015289	WB (1:2000)
Antibody	Alexa Fluor 488-conjugated affinipure goat anti-mouse IgG +IgM(H+L)	Jackson	Cat# 115-545-044; RRID:AB_2338844	IF (1:1000)
Antibody	Alexa fluor 568-goat anti-rabbit IgG (H+L) cross-adsorbed secondary antibody	Invitrogen	Cat# A11011; RRID:AB_143157	IF (1:1000)
Chemical compound, drug	FBS	VivaCell	Cat# C04001-500	
Chemical compound, drug	PBS	VivaCell	Cat# C3580-0500	
Chemical compound, drug	DMEM (high glucose)	VivaCell	Cat# C3113-0500	
Chemical compound, drug	DMEM (no glucose)	Sigma	Cat# D5030	
Chemical compound, drug	Sodium pyruvate	Sangon Biotech	Cat# A501259-0100	
Chemical compound, drug	Penicillin-streptomycin solution	VivaCell	Cat# C3421-0100	
Chemical compound, drug	Cell dissociation solution	Sartorius	Cat# 03-079-1B	
Chemical compound, drug	β-Mercaptoethanol	Sigma	Cat# M3148	
Chemical compound, drug	Eosin Y (water soluble)	Aladdin	Cat# E141405	
Chemical compound, drug	Hematoxylin	BBI	Cat# A600701-0050	
Chemical compound, drug	Oil Red O	Solarbio	Cat# IO1720	
Chemical compound, drug	Sirius red	Sangon Biotech	Cat# A500684-0500	
Chemical compound, drug	High effect paraffin cere sin	Shanghai Hualing Rehabilitation Equipment Manufacturing Plant	Cat# N/A	
Chemical compound, drug	10% Neutral formalin fix solution	BBI	Cat# E672001-0500	
Chemical compound, drug	Xylene	Tianjin Zhiyuan Chemical Reagents Co., Ltd	Cat# N/A	
Chemical compound, drug	Neutral balsam	Solarbio	Cat# G8590	
Chemical compound, drug	Isopropanol	Sangon Biotech	Cat# A507048-0500	
Chemical compound, drug	Tissue-tek OCT compound	SAKURA	Cat# REF:4583	
Chemical compound, drug	Paraformaldehyde	Sangon Biotech	Cat# A500684-0500	
Chemical compound, drug	Acetone	Chron Chemicals	Cat# N/A	
Chemical compound, drug	Sucrose	Sangon Biotech	Cat# A502792-0005	
Chemical compound, drug	Triton X-100	BBI	Cat# A600198-0500	
Chemical compound, drug	Goat serum	VivaCell	Cat# C2530-0100	
Chemical compound, drug	Tween20	BBI	Cat# A600560-0500	
Chemical compound, drug	DAPI staining solution	Beyotime	Cat# C1006	
Chemical compound, drug	Omni-Easy one-step PAGE gel fast preparation kit	Epizyme	Cat# PG213	
Chemical compound, drug	SDS	BBI	Cat# A600485-0500	
Chemical compound, drug	Glycine	BBI	Cat# A502065-0005	
Chemical compound, drug	Tris	Solarbio	Cat# T8060	
Chemical compound, drug	Methanol	Ghtech	Cat# N/A	
Chemical compound, drug	Non-fat powdered milk	BBI	Cat# NON-Fat Powdered Milk	
Chemical compound, drug	Collagenase, type 1	Diamond	Cat# A004194-0001	
Chemical compound, drug	Cytiva Percoll Centrifugation Media	Cytiva	Cat# 17089101	
Chemical compound, drug	Heparin sodium from porcine intestinal	Sangon Biotech	Cat# A603251-0001	
Chemical compound, drug	1 M HEPES	Solarbio	Cat# H1095	
Chemical compound, drug	Optiprep	Serumwerk Bernburg	Cat# 1893	
Chemical compound, drug	DNaseI, RNase-free	Thermo	Cat# EN0521	
Chemical compound, drug	CaCl2	Ghtech	Cat#10043-52-4	
Chemical compound, drug	MgSO4·7H2O	Sangon Biotech	Cat# A610329-0500	
Chemical compound, drug	Trizol reagent	Invitrogen	Cat# 15596018	
Chemical compound, drug	UltraPure DNase/RNase-free distilled water	Invitrogen	Cat# 10977015	
Chemical compound, drug	Trichloromethane	Chron Chemicals	Cat# N/A	
Chemical compound, drug	PowerUp SYBR Green Master Mix	Applied biosystems	Cat# A25742	
Chemical compound, drug	DEPC水	Biosharp	Cat# 701062	
Chemical compound, drug	MgCl_2_	Ghtech	Cat# N/A	
Chemical compound, drug	KCl	Sangon Biotech	Cat# A501159-0500	
Chemical compound, drug	NaHCO_3_	Sangon Biotech	Cat# A500873-0500	
Chemical compound, drug	NaOH	BBI	Cat# A620617-0500	
Chemical compound, drug	Palmitic acid	Sigma	Cat# P0500	
Chemical compound, drug	DMSO	Sangon Biotech	Cat# A100231-0500	
Chemical compound, drug	Proteinase K solution (20 mg/mL)	BBI	Cat# B600169-0002	
Chemical compound, drug	Glycerol gelatin aqueous slide mounting medium	Solarbio	Cat# S2150	
Chemical compound, drug	XF basal medium	Agilent	Cat#103334–100	
Chemical compound, drug	XF 200 mmol/L glutamine solution	Agilent	Cat#103579–100	
Chemical compound, drug	BD Pharmingen Stain Buer (FBS)	BD Biosciences	Cat# 554656	
Chemical compound, drug	Draq7	BD Biosciences	Cat# 564904	
Chemical compound, drug	High-fat rodent diet with 1.25%cholesterol	Research diet	Cat# d12108c	
Chemical compound, drug	Methionine and choline-deficient diet	Research Diet	Cat# A02082002BR	
Chemical compound, drug	Proteinase K solution (20 mg/mL)	BBI	Cat# B600169-0002	
Chemical compound, drug	Glycerol Gelatin aqueous slide mounting medium	Solarbio	Cat# S2150	
Chemical compound, drug	XF basal medium	Agilent	Cat#103334–100	
Chemical compound, drug	XF 200 mmol/L Glutamine solution	Agilent	Cat#103579–100	
Chemical compound, drug	BD Pharmingen Stain Buer (FBS)	BD Biosciences	Cat# 554656	
Chemical compound, drug	Draq7	BD Biosciences	Cat# 564904	
Chemical compound, drug	Dynabeads M-280 streptavidin	Invitrogen	Cat# 11205D	
Chemical compound, drug	2-NBDG	Invitrogen	Cat# N13195	
Peptide, recombinant protein	Recombinant mouse Chi3l1	SB	Cat# 50929-M08H	
Commercial assay or kit	TMR (red) Tunel cell apoptosis detection kit	Servicebio	Cat# G1502-100T	
Commercial assay or kit	Calcein/PI cell viability /cytotoxicity assay kit	Beyotime	Cat# C2015M	
Commercial assay or kit	Alanine aminotransferase assay kit	Nanjing Jiancheng Bioengineering Institute	Cat# C009-2-1	
Commercial assay or kit	Aspartate aminotransferase assay kit	Nanjing Jiancheng Bioengineering Institute	Cat# C010-2-1	
Commercial assay or kit	Total cholesterol assay kit	Nanjing Jiancheng Bioengineering Institute	Cat# A111-1-1	
Commercial assay or kit	Triglyceride assay kit	Nanjing Jiancheng Bioengineering Institute	Cat# A110-1-1	
Commercial assay or kit	Seahorse XF glycolysis stress test kit	Agilent	Cat# 103020–100	
Commercial assay or kit	PrimeScript II 1st strand cDNA synthesis kit	TaKaRa	Cat# 6210B	
Software, algorithm	GraphPad Prism	GraphPad Software	RRID:SCR_002798	https://www.graphpad.com
Software, algorithm	Flowjo V10	Flowjo Software	RRID:SCR_008520	https://www.flowjo.com/
Software, algorithm	ImageJ	National Institutes of Health	RRID:SCR_003070	https://imagej.nih.gov/ij/
Software, algorithm	SPSS	IBM SPSS software	RRID:SCR_002865	https://www.ibm.com/
Software, algorithm	Seahorse wave	Agilent Technologies	RRID:SCR_024491	https://www.agilent.com/
Software, algorithm	Zen microscope software	ZEISS	RRID:SCR_013672	https://www.zeiss.com.cn/
Cell line (*Mus musculus*, male)	NCTC clone 929 (L-929)	ATCC	CCL-1 RRID:CVCL_0462	L929 was a gift from Dr. Guangxun Meng (Hainan Academy of Medical Sciences)
Strain, strain background (*Mus musculus*, male)	*Chi3l1^-/-^*	GemPharmatech Co., Ltd.	RRID:IMSR_GPT:T014402	Genetic modification: constitutive knockout
Strain, strain background (*Mus musculus*, male)	*Chi3l1^flox/flox^*	GemPharmatech Co., Ltd.	RRID:IMSR_GPT:T013652	Genetic modification: floxed allele (homozygous)
Strain, strain background (*Mus musculus*, male)	*Clec4f cre*	GemPharmatech Co., Ltd.	RRID:IMSR_GPT:T036801	Genetic modification: Cre recombinase transgene under Clec4f promoter
Strain, strain background (*Mus musculus*, male)	*Lyz2 cre*	GemPharmatech Co., Ltd.	RRID:IMSR_GPT:T003822	Genetic modification: Cre recombinase transgene under Lyz2 promoter
Strain, strain background (*Mus musculus*, male)	Rosa26^LSL-tdTomato/+^	Jackson Laboratory	RRID:IMSR_JAX:007909	Genetic modification: Conditional tdTomato reporter
Other	Bone-marrow-derived macrophage (BMDM)	This paper	PMID:42187013	Strain: C57BL/6 J
Other	Kupffer cell (KC)	This paper	PMID:42187013	Strain: C57BL/6 J
