# Supplementary material for: Differential regulation of hepatic macrophage fate by Chi3l1 in metabolic dysfunction-associated steatotic liver disease
Source: eLife. 2026 Jun 26;14:RP107023. doi: 10.7554/eLife.107023 (PMC13309125; doi:10.7554/eLife.107023)
Supplement: Figure 1—source data 2. [file elife-107023-fig1-data2.pdf]

Raw unedited membranes

Figure 1C

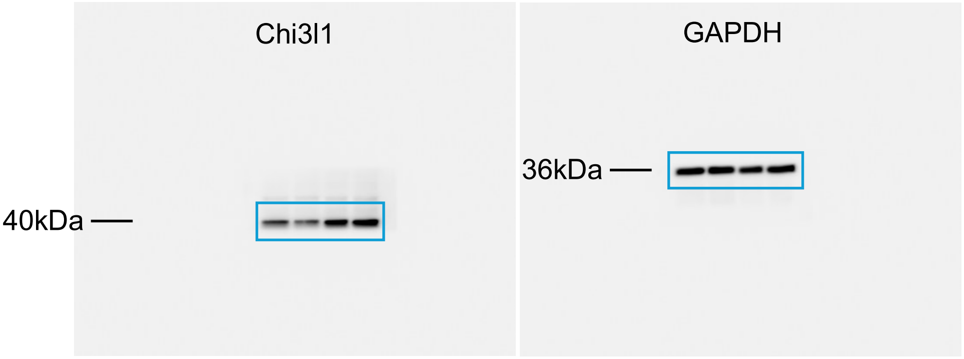

Figure 1D

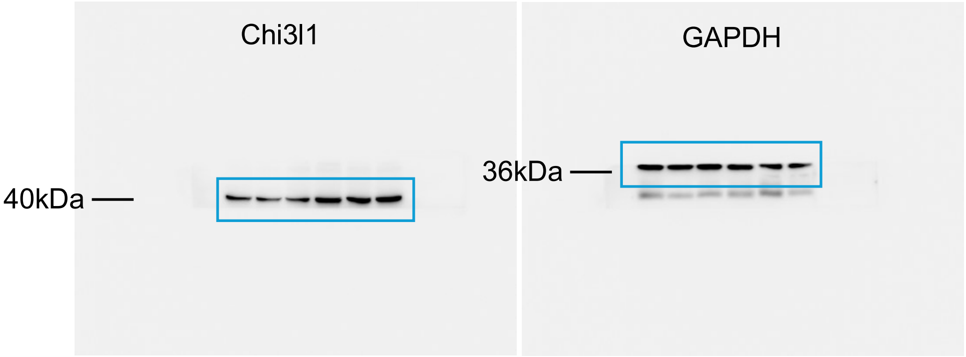

Figure 1-Source Data 2. Original membranes corresponding to Figure 1C and 1D. Chi3l1 in isolated Kupffer cells (KCs, Figure 1C) from mice fed either NCD or HFHC diet; The lower membranes show whole liver tissue (Liver, Figure 1D) from mice fed either NCD (first three lanes) or HFHC (lanes 4, 5, and 6) diet.
