# Supplementary figures and images for: Differential regulation of hepatic macrophage fate by Chi3l1 in metabolic dysfunction-associated steatotic liver disease
Source: eLife. 2026 Jun 26;14:RP107023. doi: 10.7554/eLife.107023 (PMC13309125; doi:10.7554/eLife.107023)

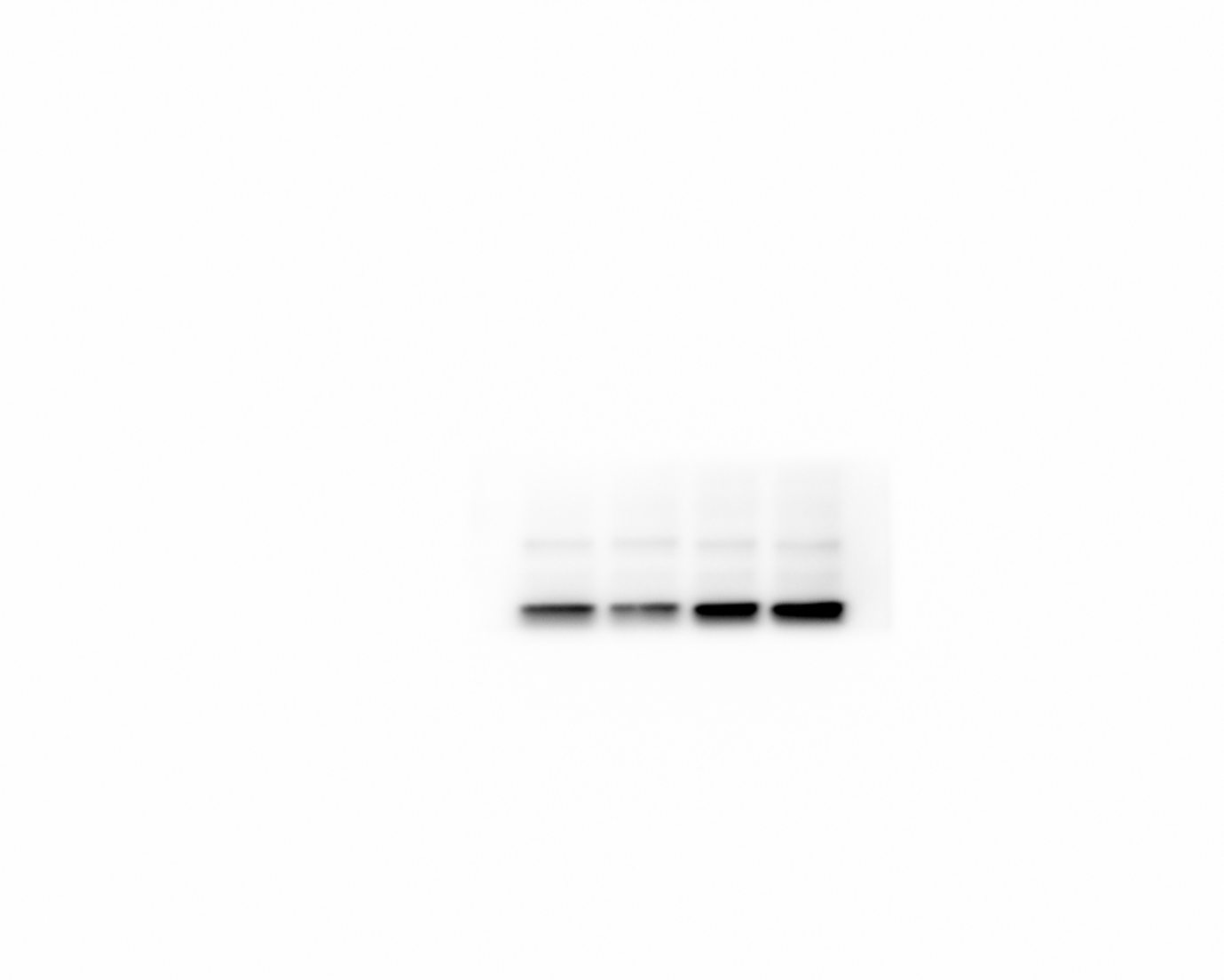

Supplement: Figure 1—source data 3. [file elife-107023-fig1-data3.zip › Figure 1-Source Data 3/Figure 1C-Chi3l1.tif]

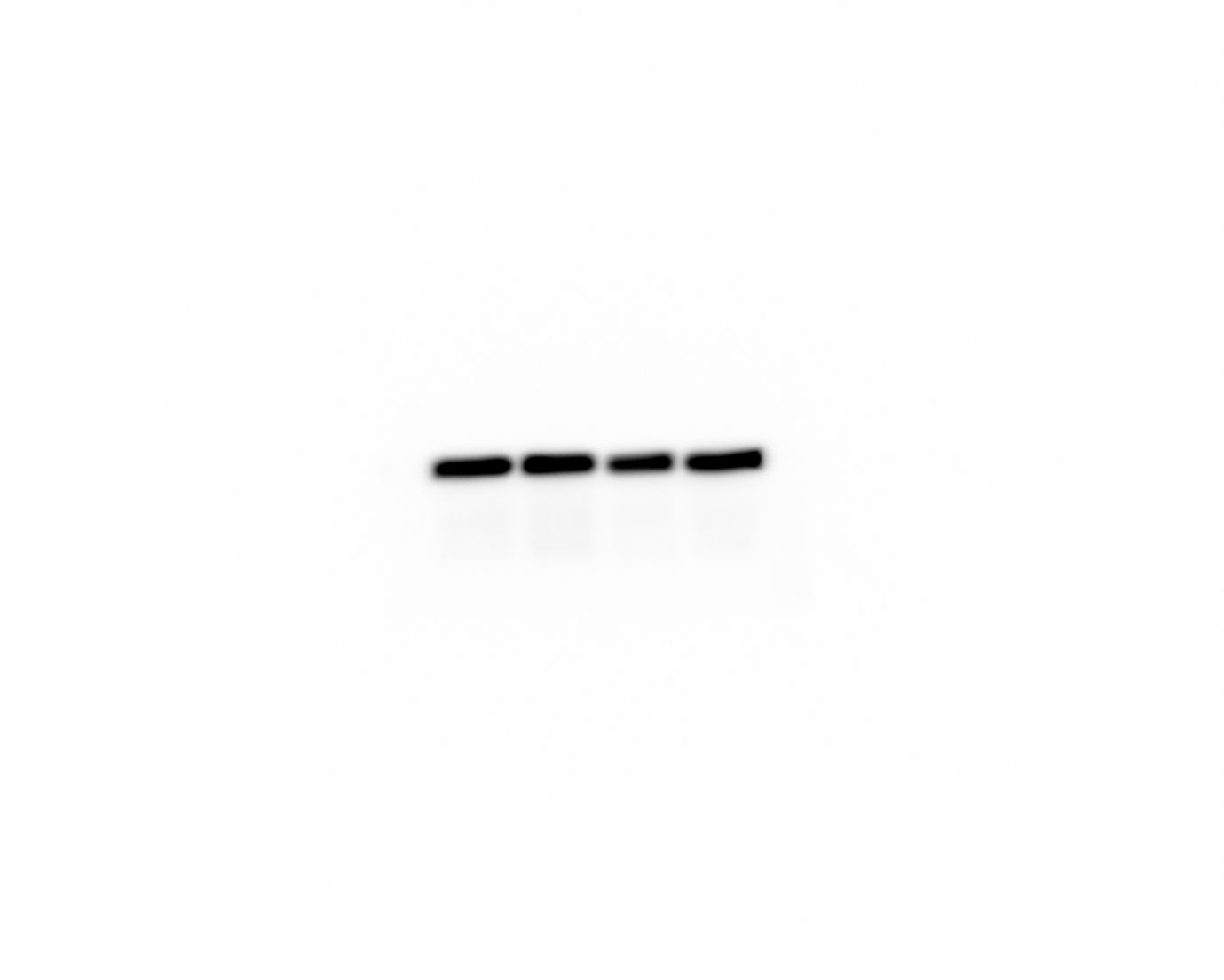

Supplement: Figure 1—source data 3. [file elife-107023-fig1-data3.zip › Figure 1-Source Data 3/Figure 1C-GAPDH.tif]

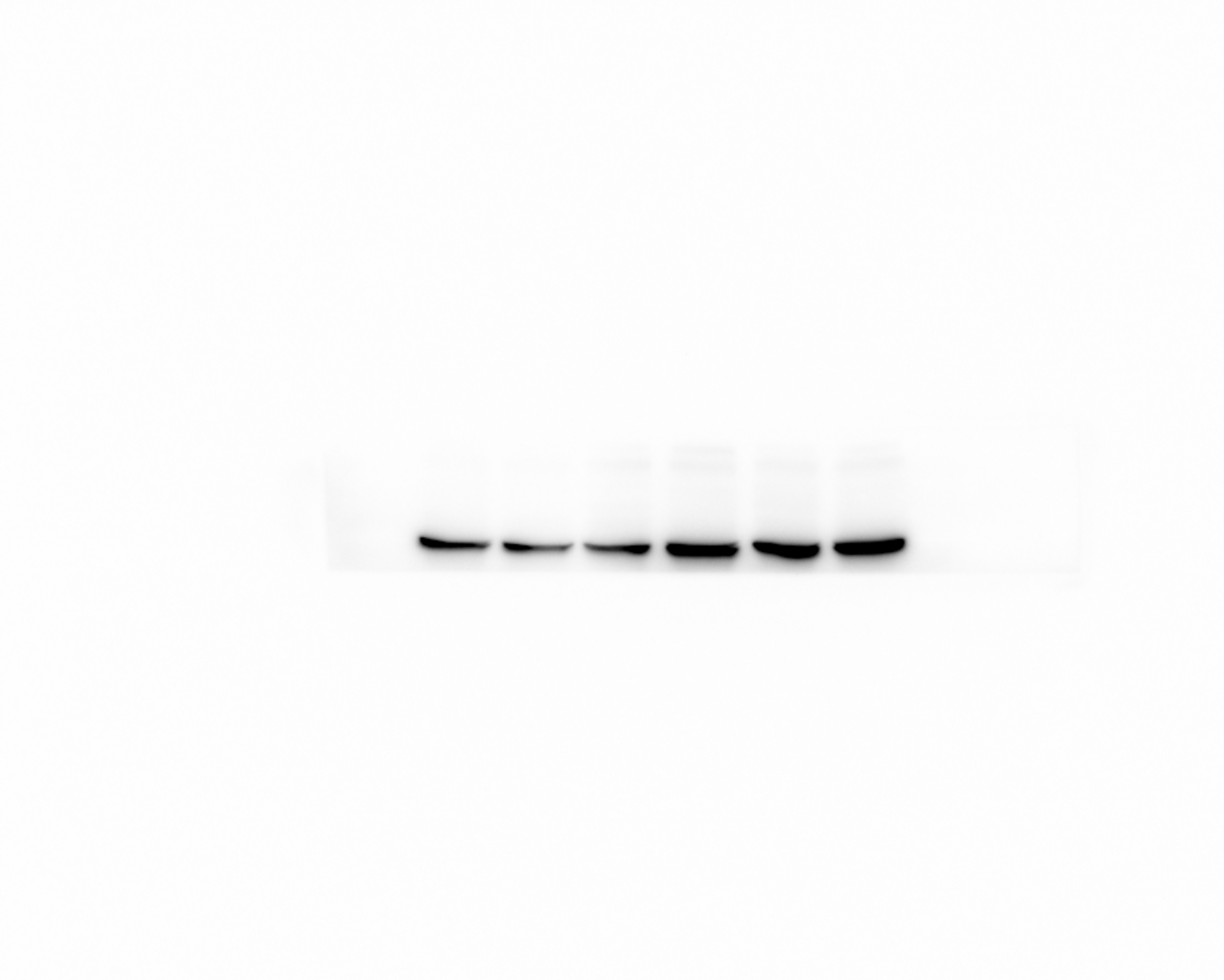

Supplement: Figure 1—source data 3. [file elife-107023-fig1-data3.zip › Figure 1-Source Data 3/Figure 1D-Chi3l1.tif]

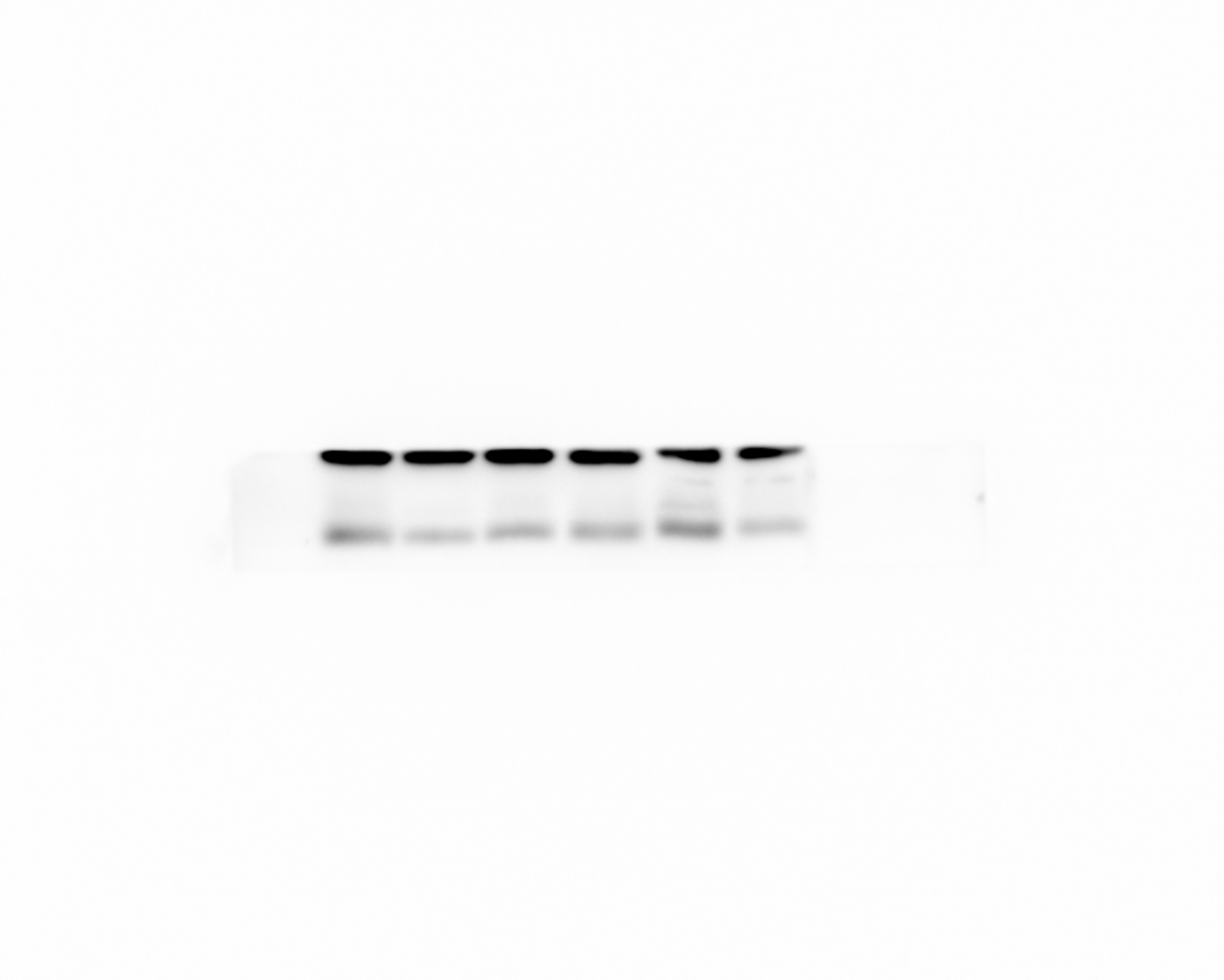

Supplement: Figure 1—source data 3. [file elife-107023-fig1-data3.zip › Figure 1-Source Data 3/Figure 1D-GAPDH.tif]

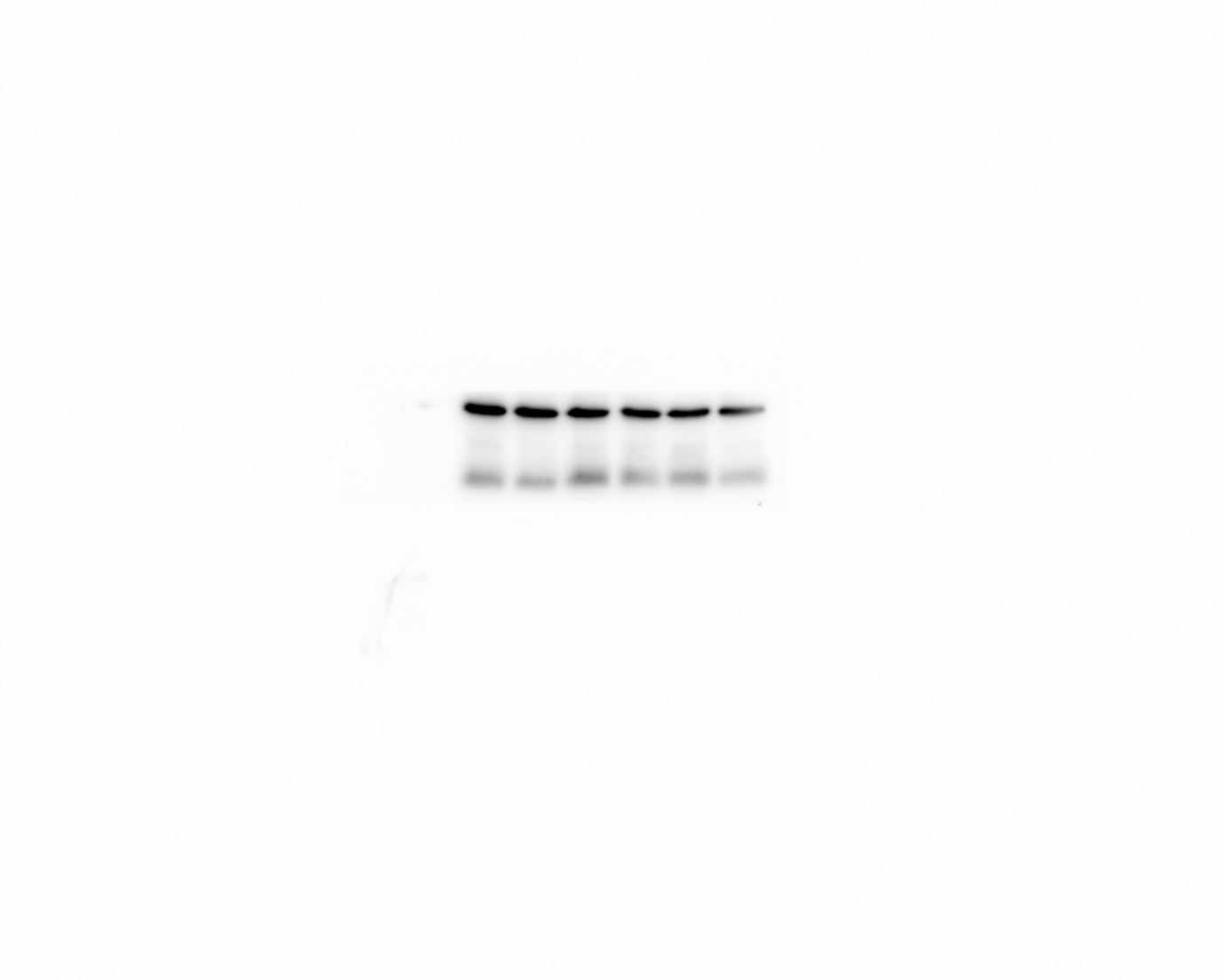

Supplement: Figure 1—figure supplement 1—source data 3. [file elife-107023-fig1-figsupp1-data3.zip › Figure 1-Figure supplement 1-Source Data 6/Figure 1-Figure supplement 1-GAPDH.tif]

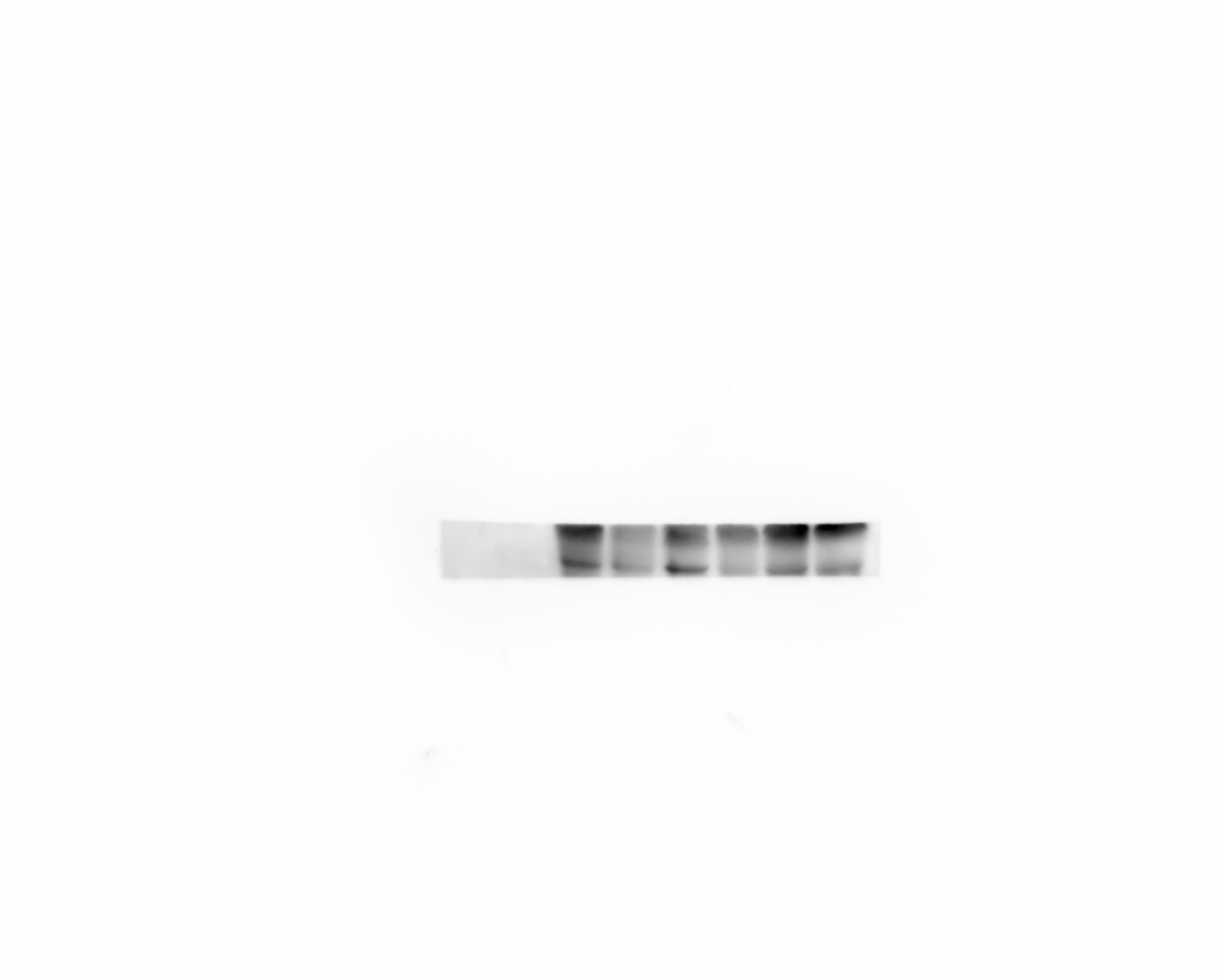

Supplement: Figure 1—figure supplement 1—source data 3. [file elife-107023-fig1-figsupp1-data3.zip › Figure 1-Figure supplement 1-Source Data 6/Figure 1-Figure supplement 1-a┴-SMA.tif]

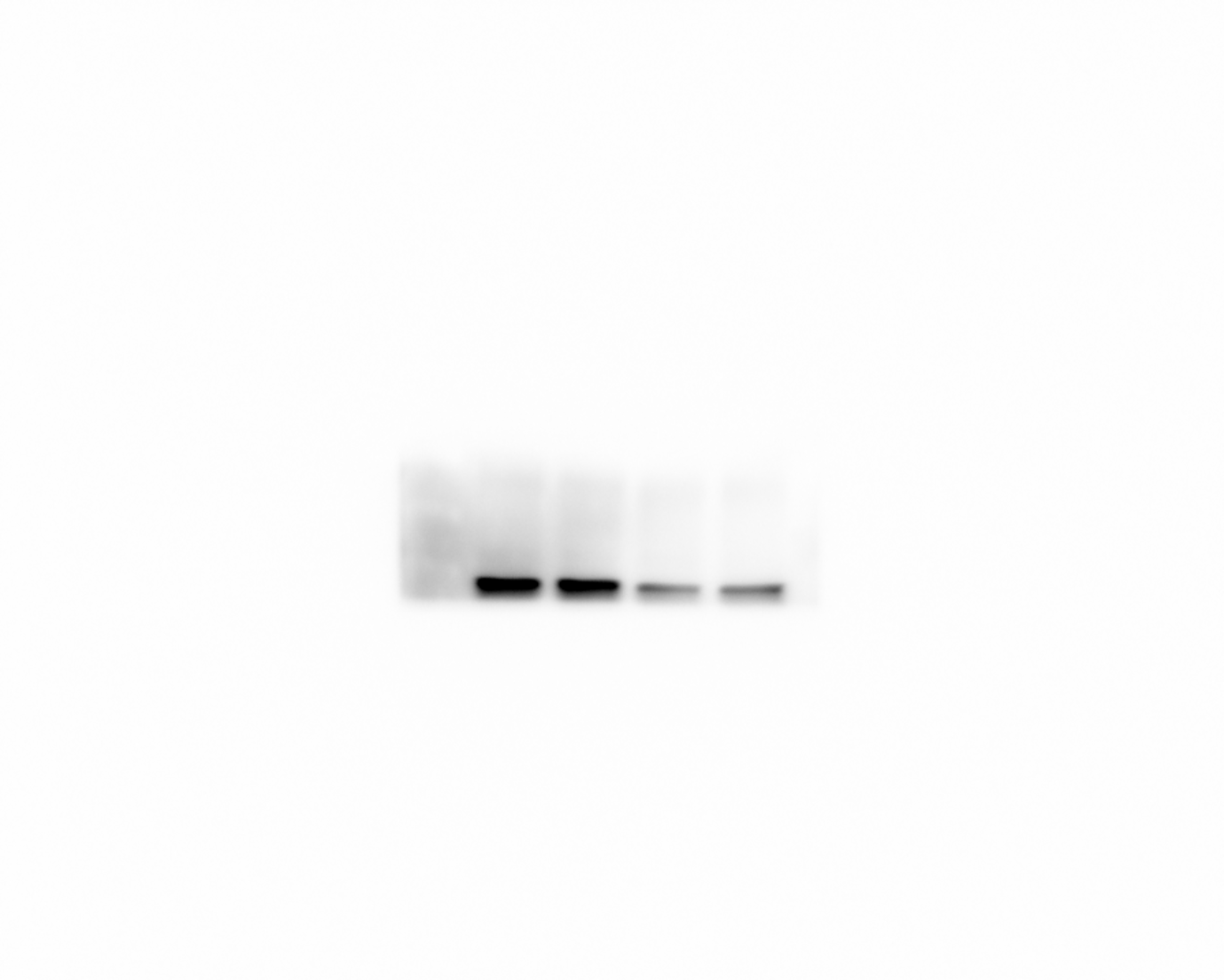

Supplement: Figure 2—figure supplement 1—source data 3. [file elife-107023-fig2-figsupp1-data3.zip › Figure 2-Figure supplement 1-Source Data 4/Figure 2-Figure supplement 1C-Chi3l1.tif]

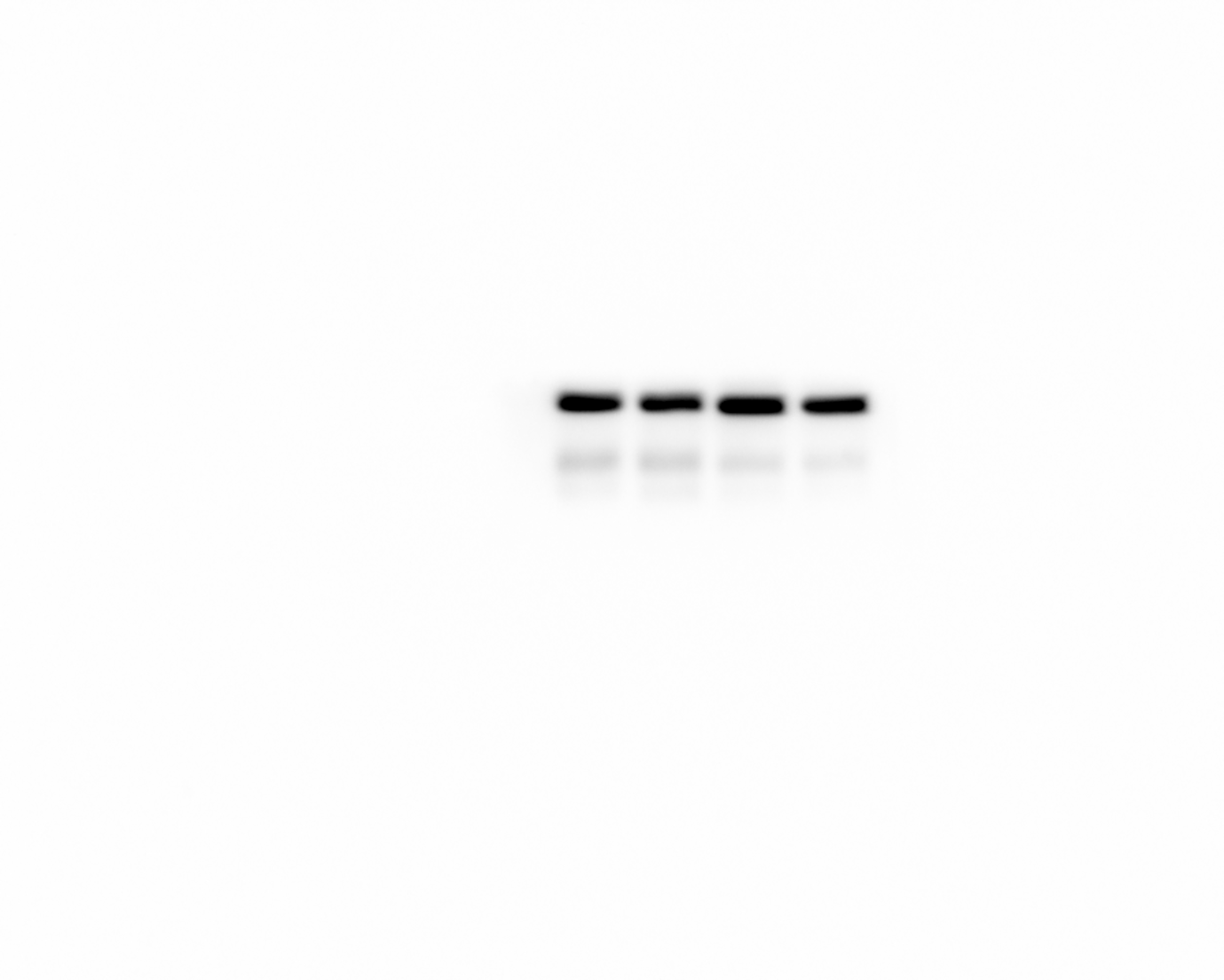

Supplement: Figure 2—figure supplement 1—source data 3. [file elife-107023-fig2-figsupp1-data3.zip › Figure 2-Figure supplement 1-Source Data 4/Figure 2-Figure supplement 1C-GAPDH.tif]

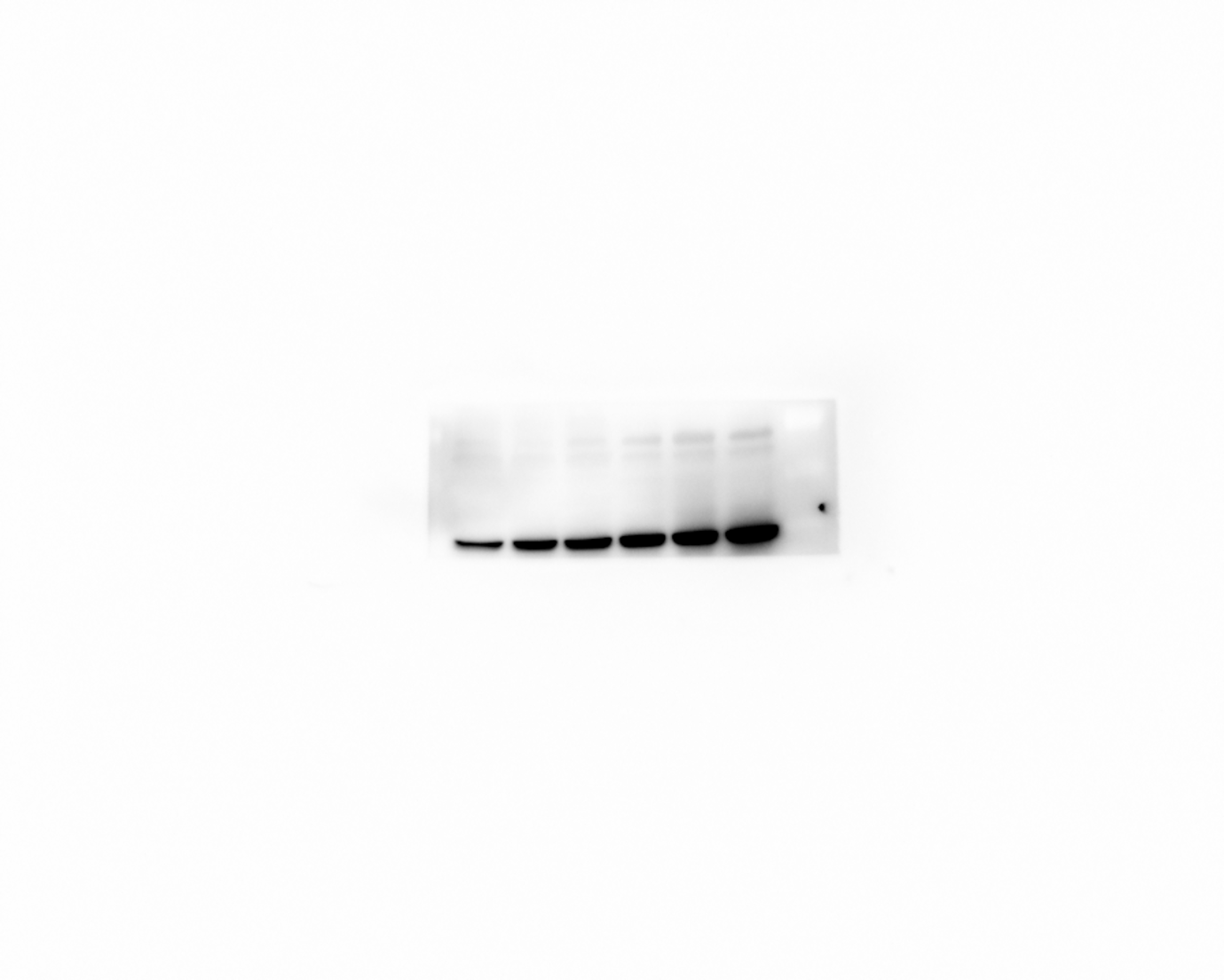

Supplement: Figure 3—source data 3. [file elife-107023-fig3-data3.zip › Figure 3-Source Data 3/Figure 3B-Chi3l1.tif]

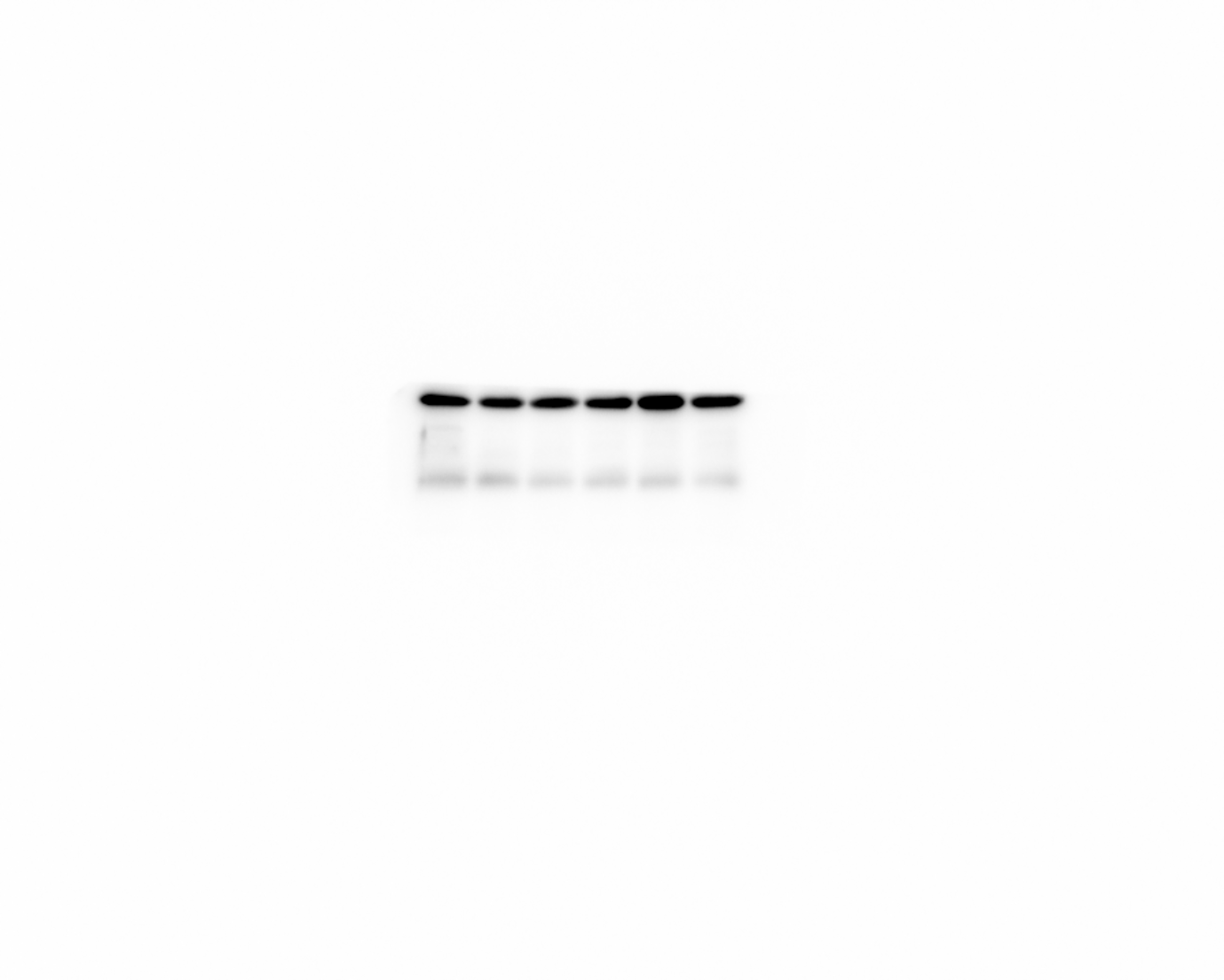

Supplement: Figure 3—source data 3. [file elife-107023-fig3-data3.zip › Figure 3-Source Data 3/Figure 3B-GAPDH.tif]

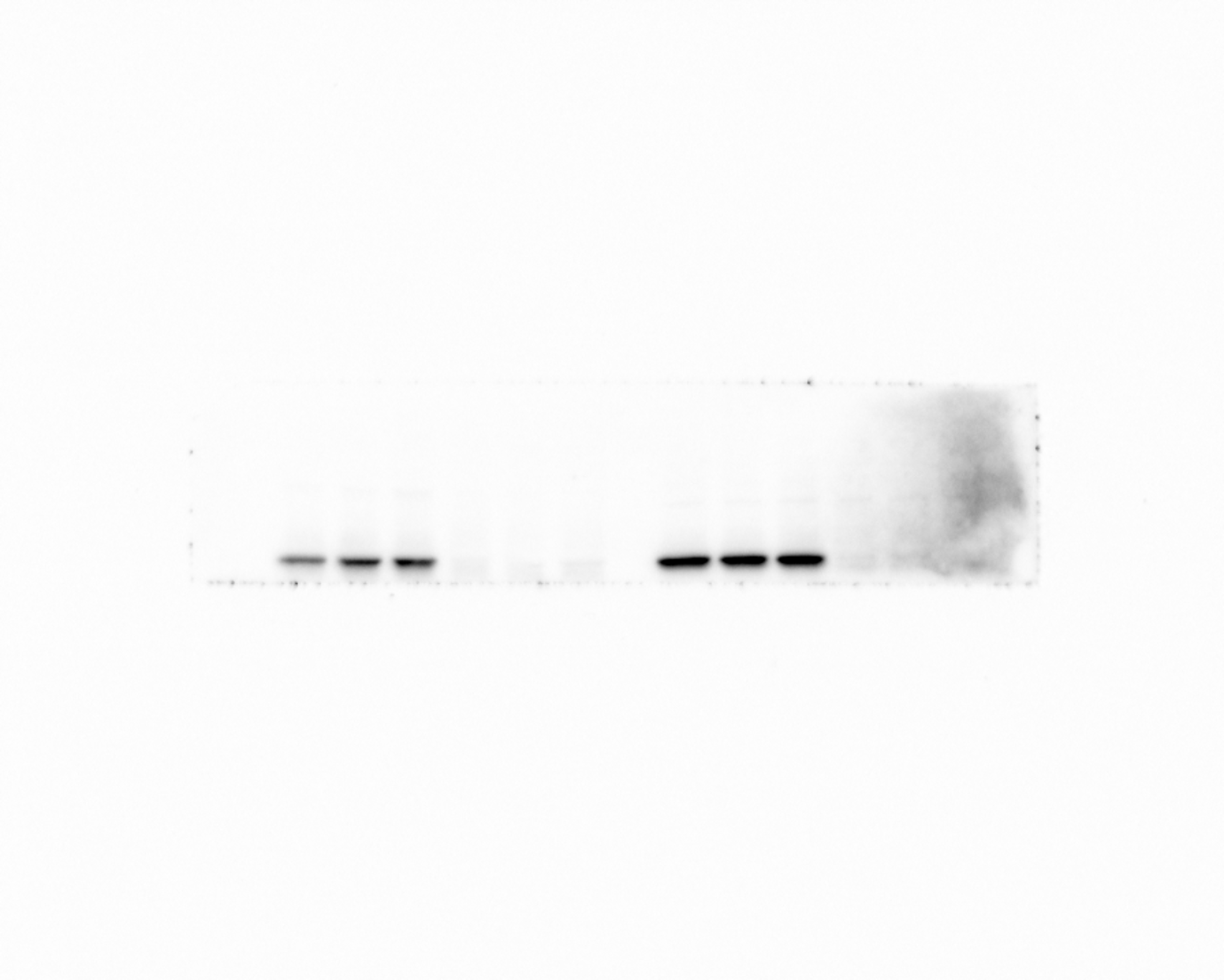

Supplement: Figure 3—figure supplement 1—source data 3. [file elife-107023-fig3-figsupp1-data3.zip › Figure 3-Figure supplement 1-Source Data 6/Figure 3-Figure supplement 1C-BMDM-Chi3l1.tif]

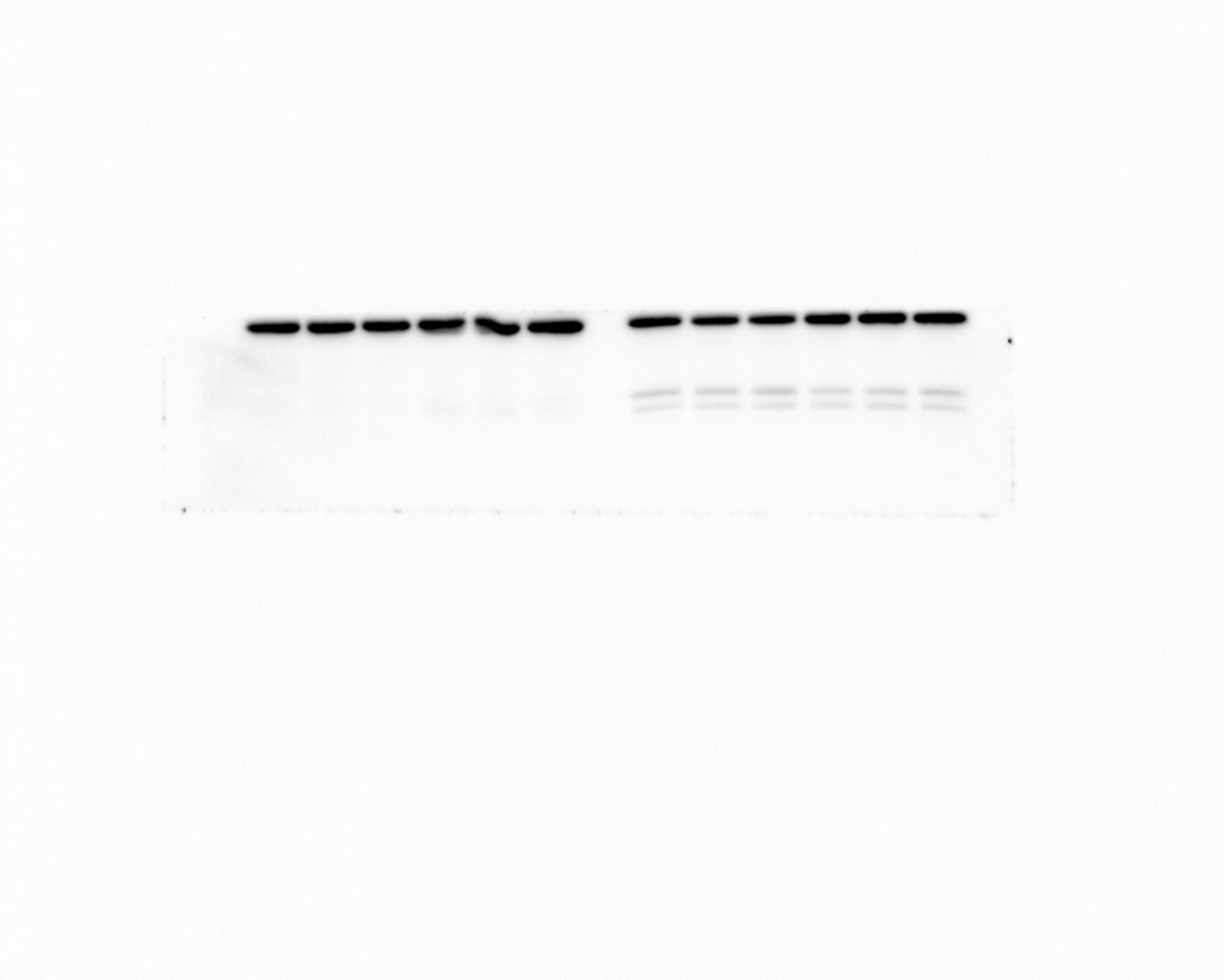

Supplement: Figure 3—figure supplement 1—source data 3. [file elife-107023-fig3-figsupp1-data3.zip › Figure 3-Figure supplement 1-Source Data 6/Figure 3-Figure supplement 1C-BMDM-GAPDH.tif]

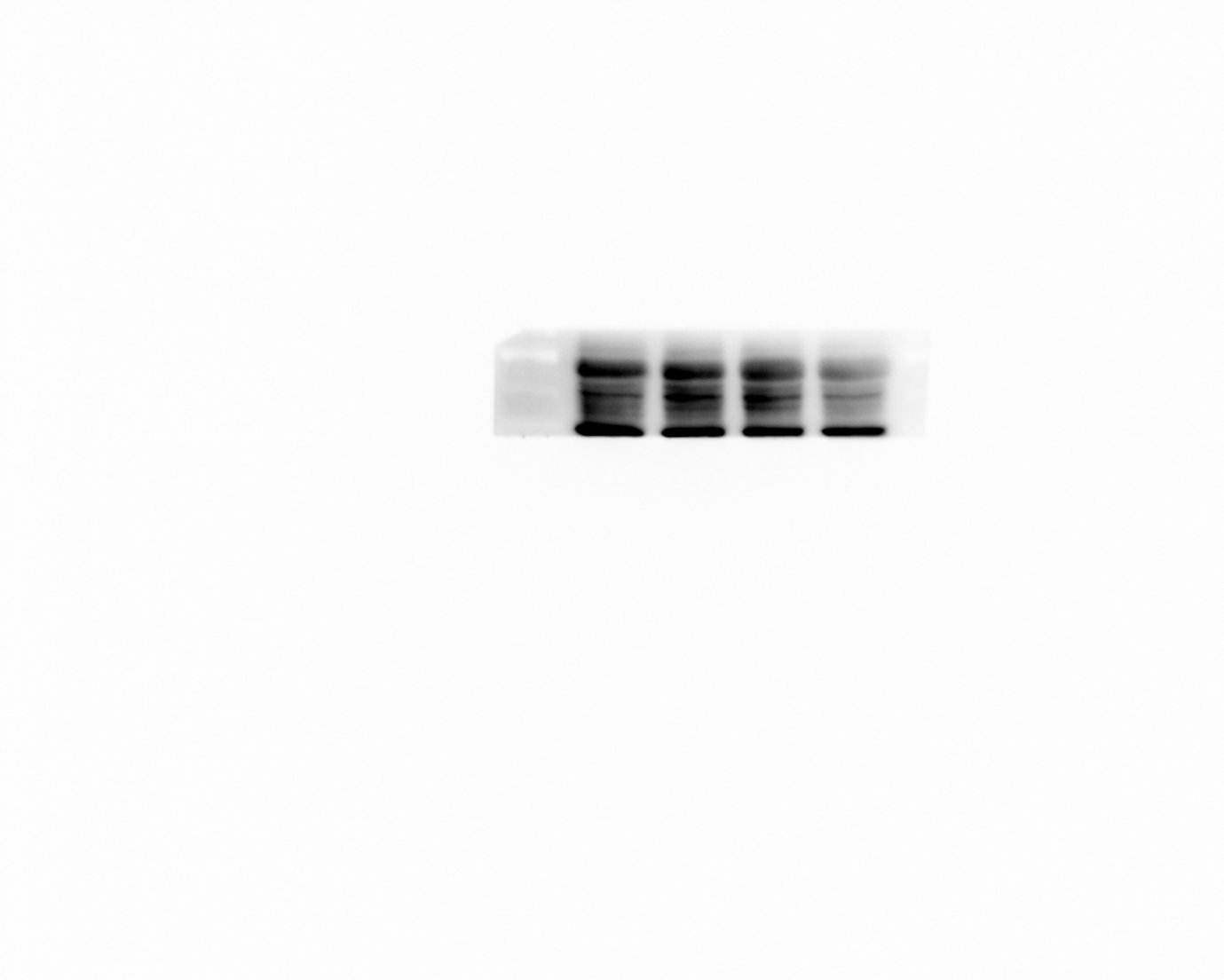

Supplement: Figure 3—figure supplement 1—source data 3. [file elife-107023-fig3-figsupp1-data3.zip › Figure 3-Figure supplement 1-Source Data 6/Figure 3-Figure supplement 1C-KCs-Chi3l1.tif]

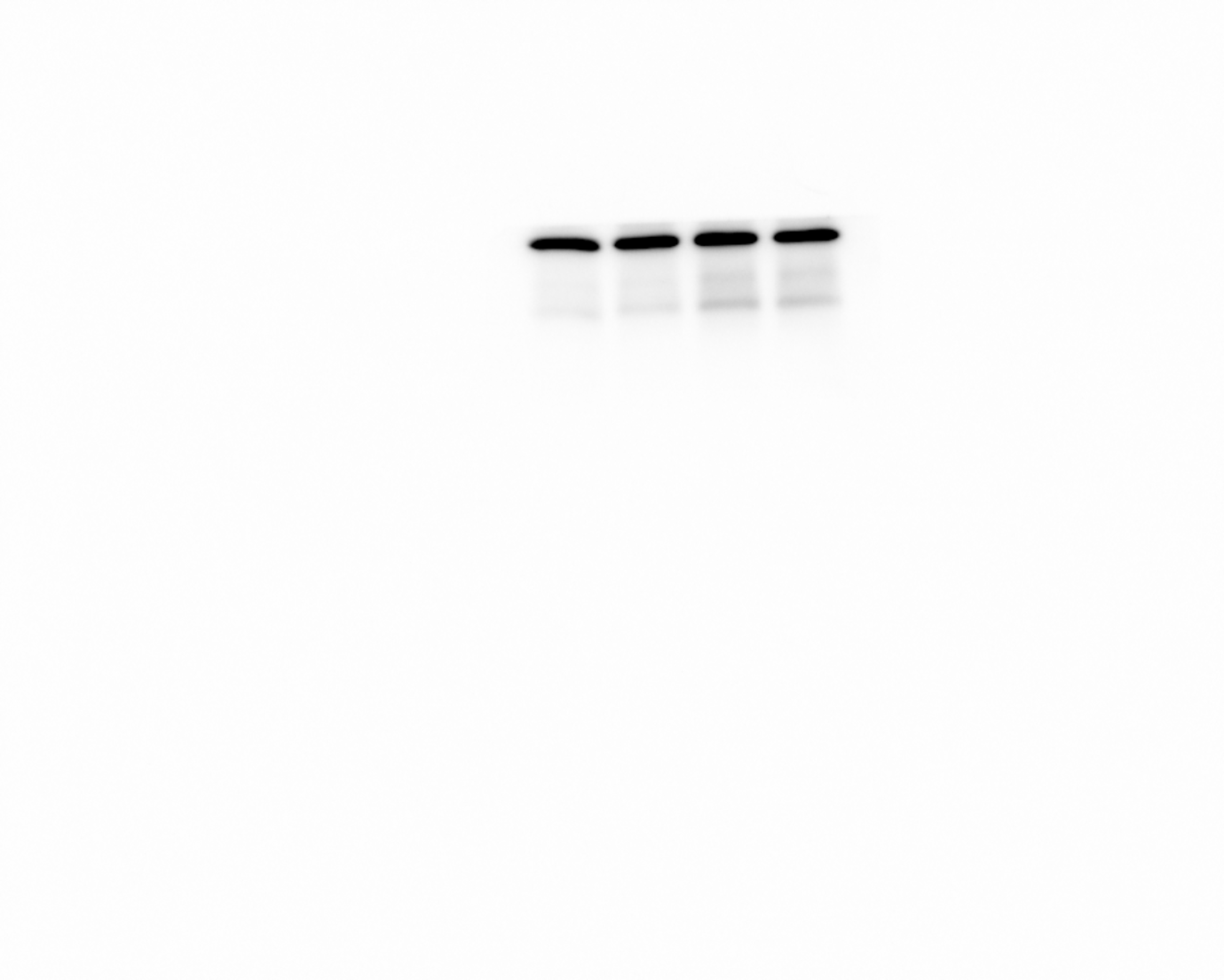

Supplement: Figure 3—figure supplement 1—source data 3. [file elife-107023-fig3-figsupp1-data3.zip › Figure 3-Figure supplement 1-Source Data 6/Figure 3-Figure supplement 1C-KCs-GAPDH.tif]

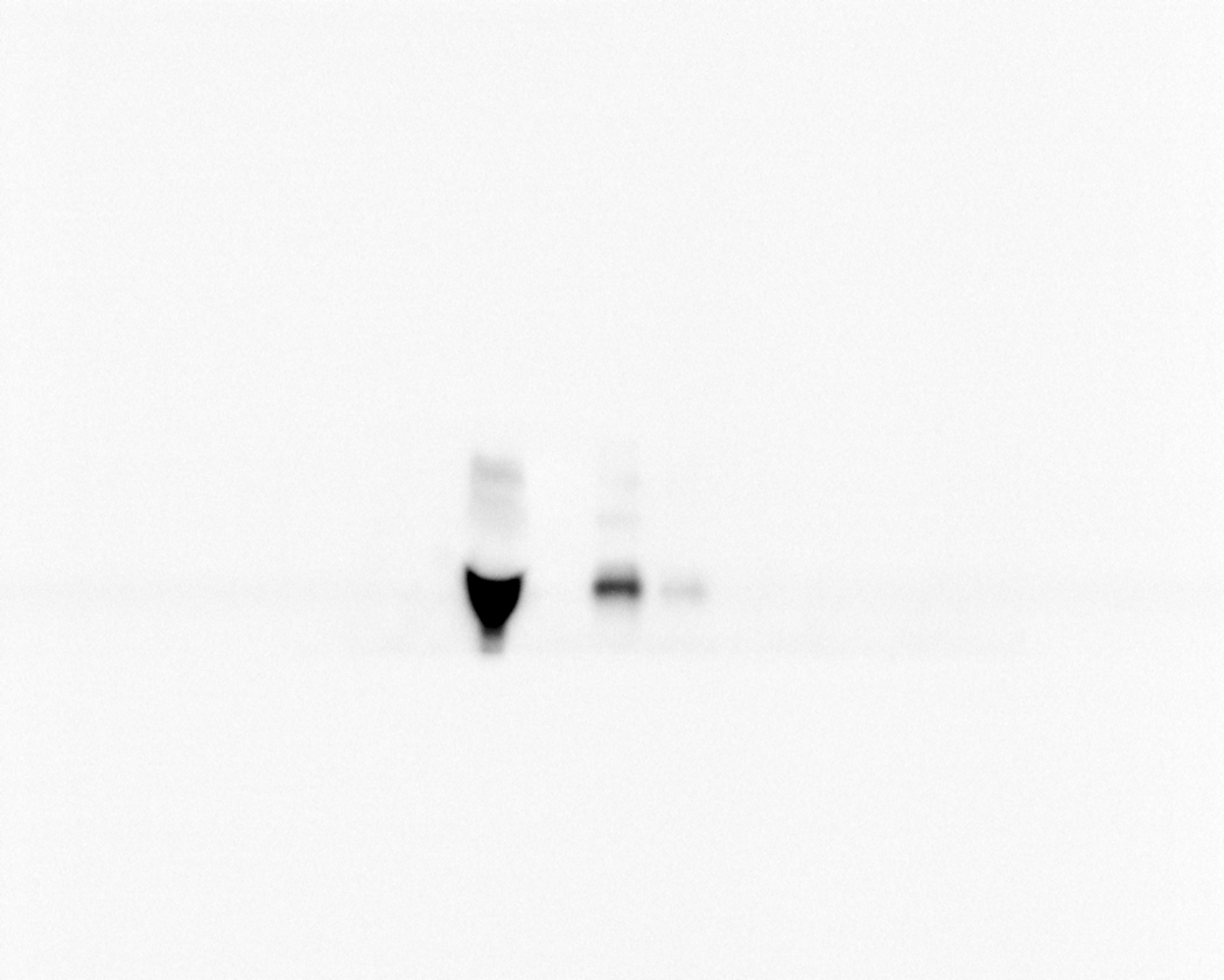

Supplement: Figure 6—source data 3. [file elife-107023-fig6-data3.zip › Figure 6-Source Data 3/Figure 6D-Chi3l1.tif]

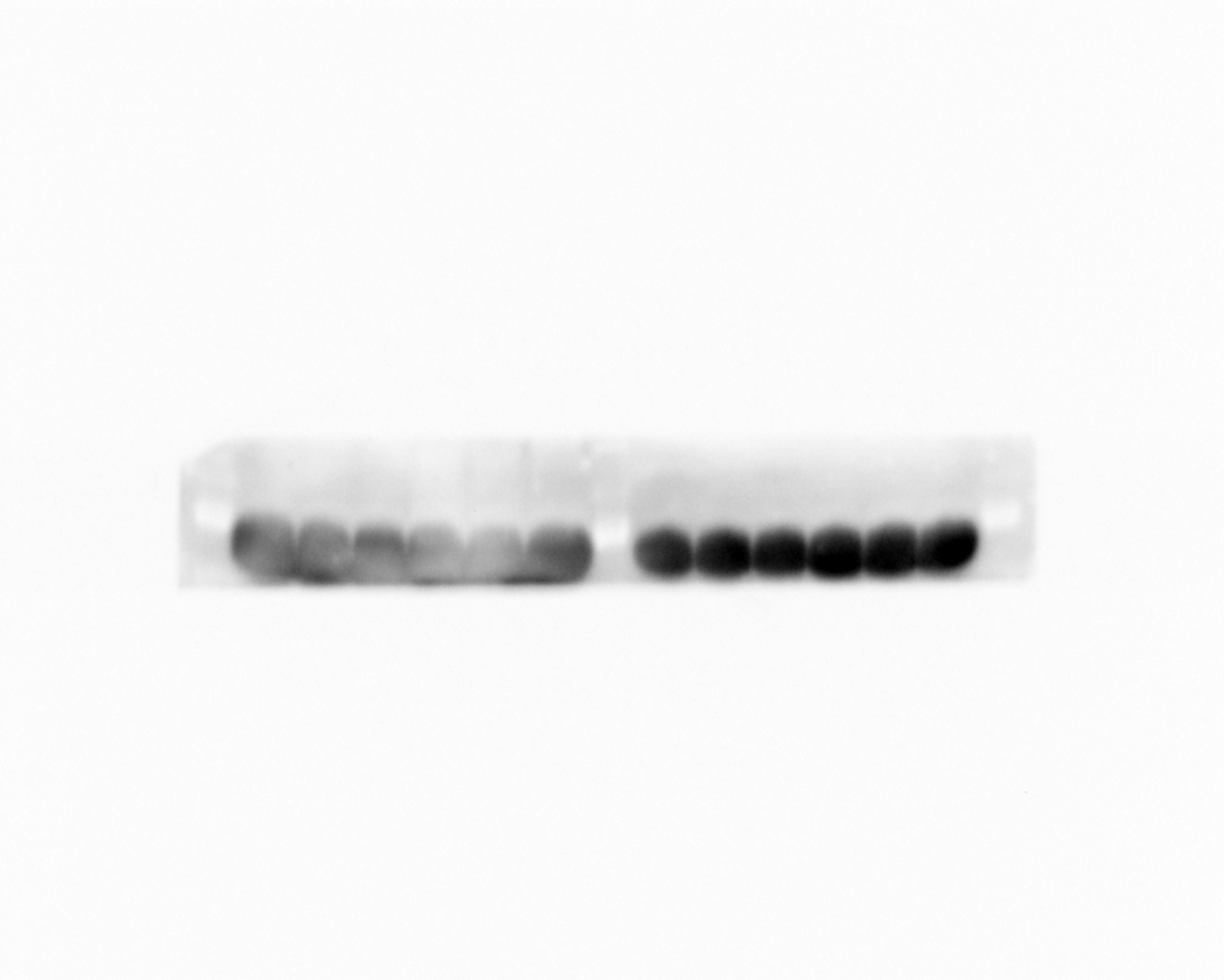

Supplement: Figure 6—source data 3. [file elife-107023-fig6-data3.zip › Figure 6-Source Data 3/Figure 6F-Albumin.tif]

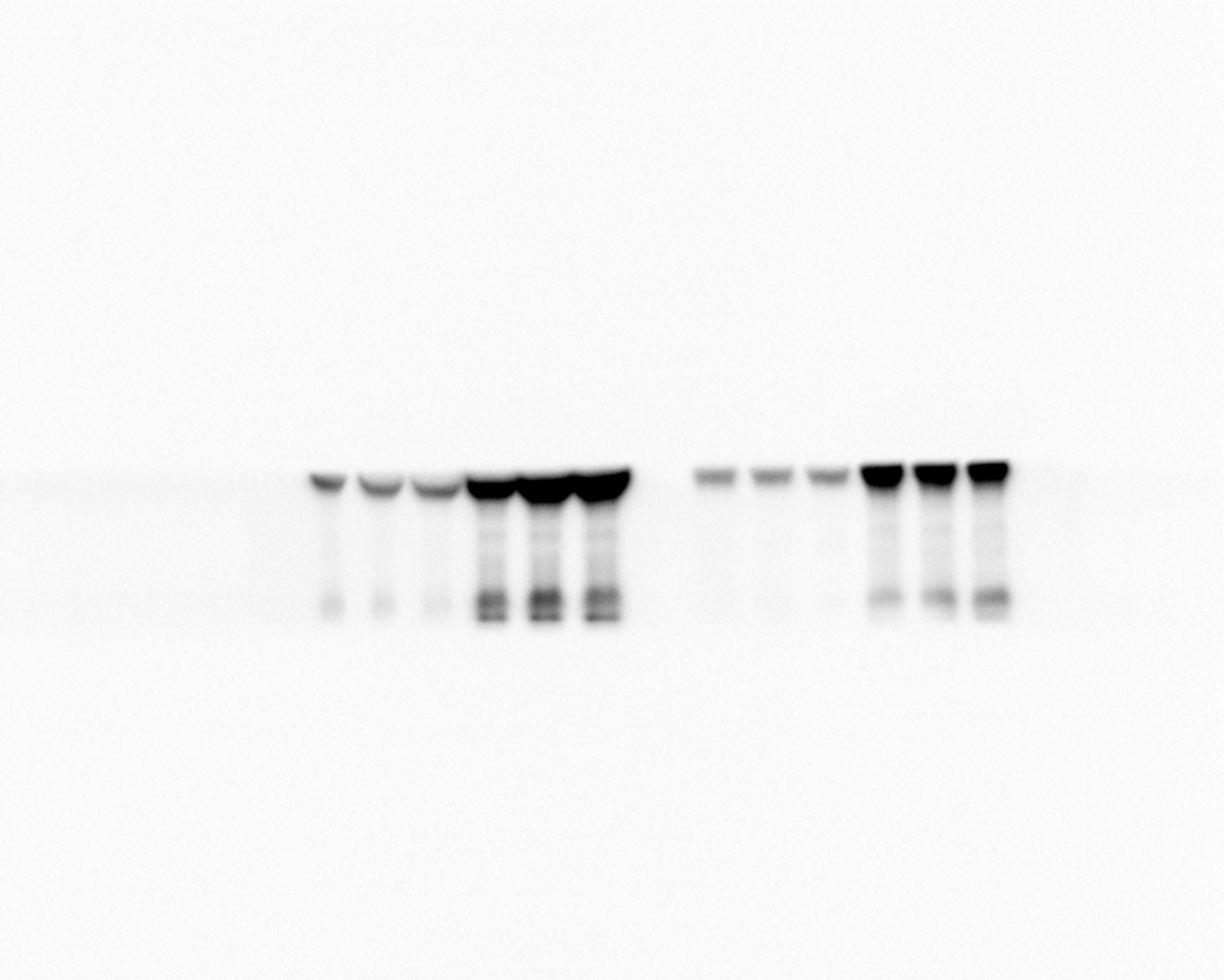

Supplement: Figure 6—source data 3. [file elife-107023-fig6-data3.zip › Figure 6-Source Data 3/Figure 6F-Chi3l1.tif]

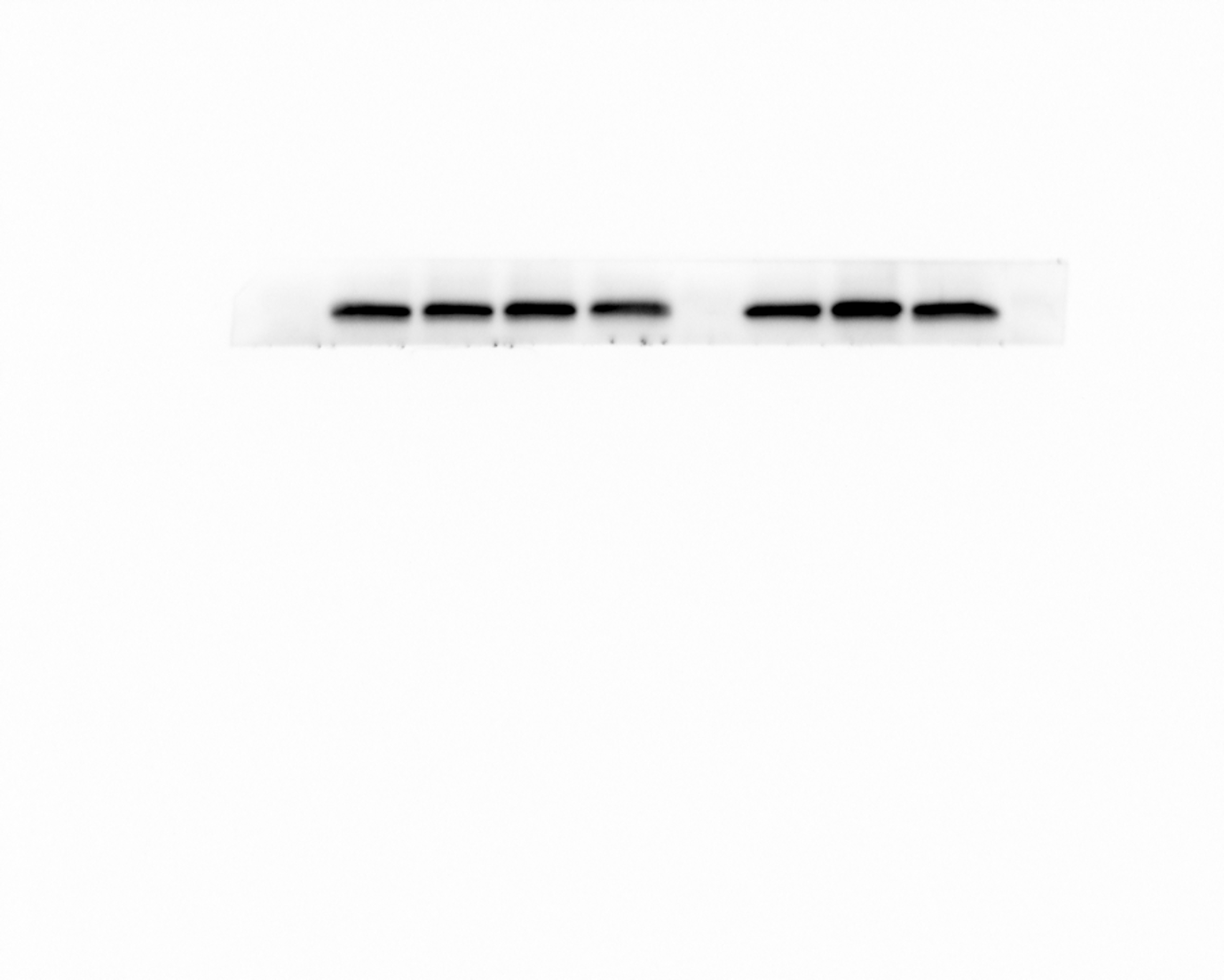

Supplement: Figure 7—source data 3. [file elife-107023-fig7-data3.zip › Figure 7-Source Data 3/Figure 7E-Casp3.tif]

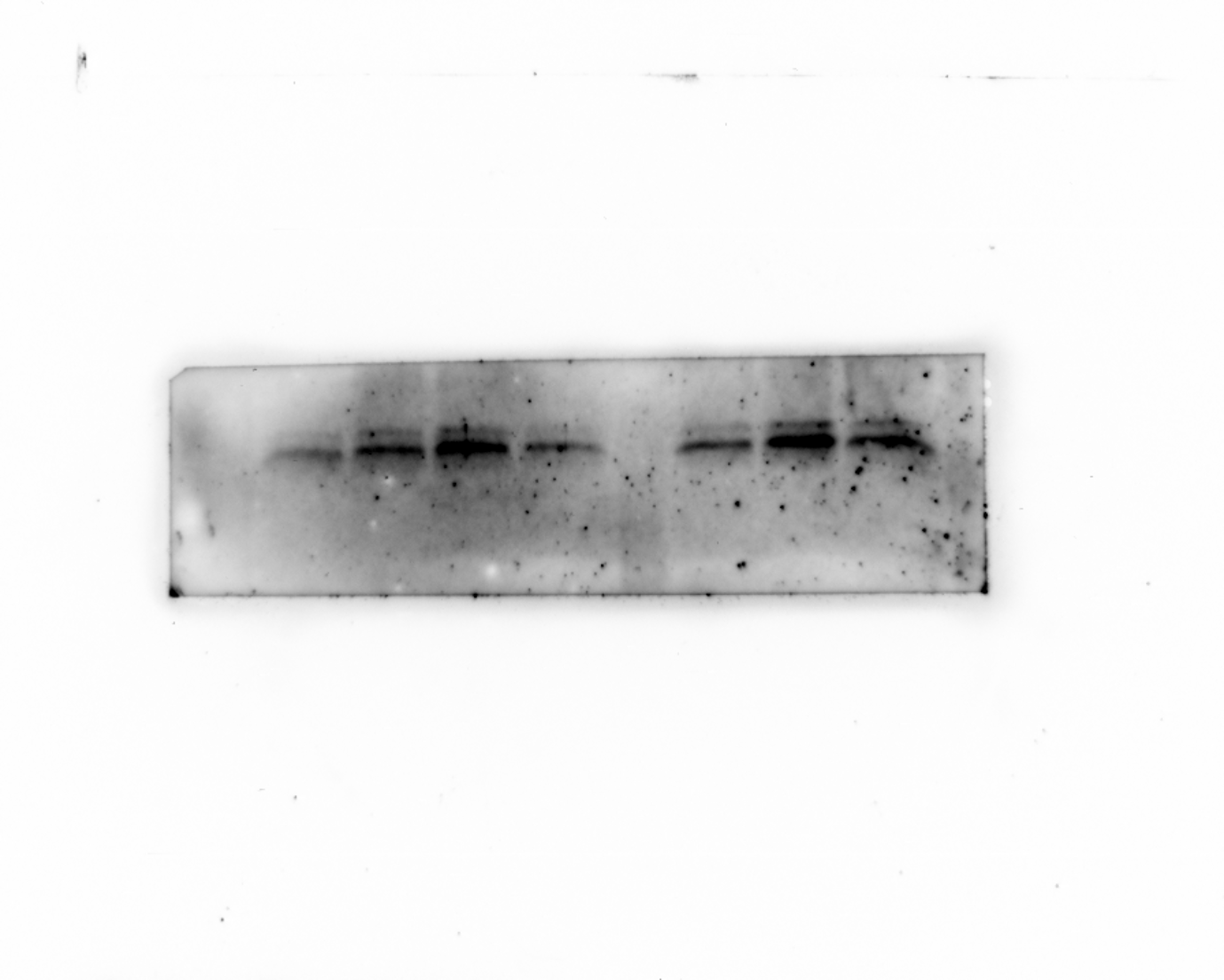

Supplement: Figure 7—source data 3. [file elife-107023-fig7-data3.zip › Figure 7-Source Data 3/Figure 7E-Cl casp3.tif]

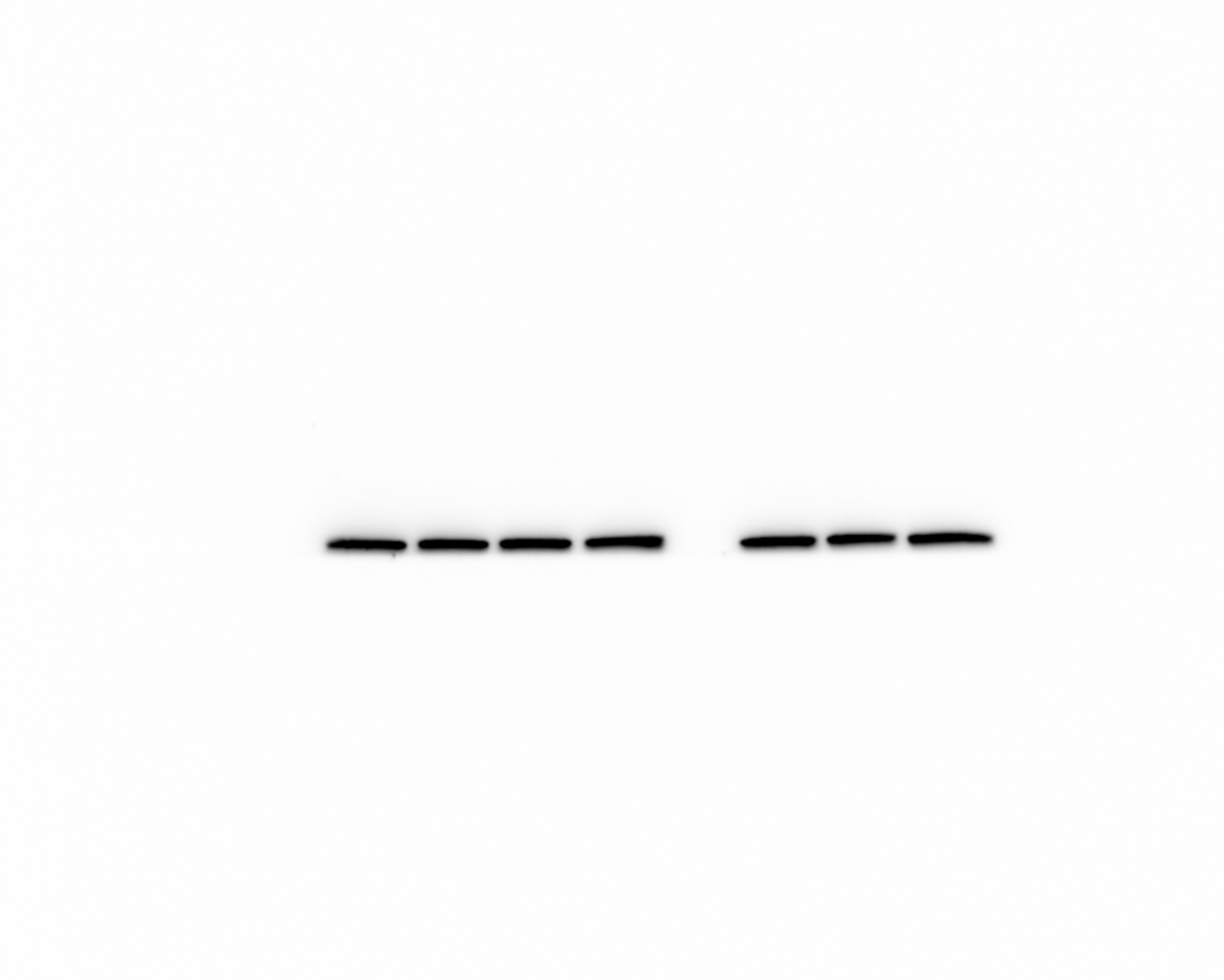

Supplement: Figure 7—source data 3. [file elife-107023-fig7-data3.zip › Figure 7-Source Data 3/Figure 7E-a┬ actin.tif]
