# Supplementary material for: Differential regulation of hepatic macrophage fate by Chi3l1 in metabolic dysfunction-associated steatotic liver disease
Source: eLife. 2026 Jun 26;14:RP107023. doi: 10.7554/eLife.107023 (PMC13309125; doi:10.7554/eLife.107023)
Supplement: Figure 1—figure supplement 1—source data 2. [file elife-107023-fig1-figsupp1-data2.pdf]

Raw unedited membranes

Figure 1-Figure supplement 1B

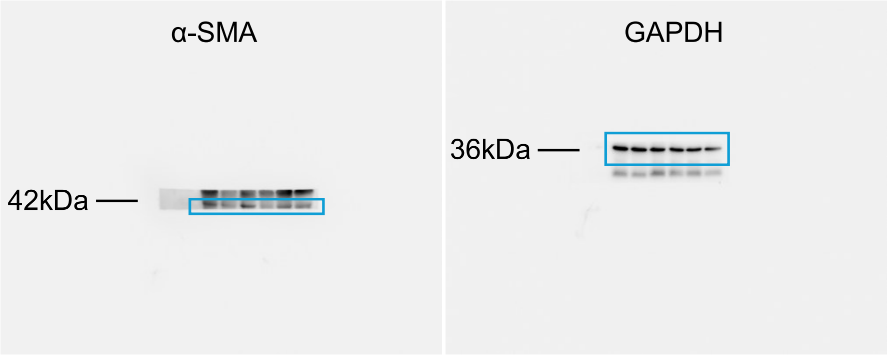

Figure 1-Figure supplement 1-Source Data 5. Original membranes corresponding to Figure 1-Figure supplement 1B. α-SMA expression in whole liver lysates from NCD-and HFHC-fed mice.
