# Supplementary material for: Differential regulation of hepatic macrophage fate by Chi3l1 in metabolic dysfunction-associated steatotic liver disease
Source: eLife. 2026 Jun 26;14:RP107023. doi: 10.7554/eLife.107023 (PMC13309125; doi:10.7554/eLife.107023)
Supplement: Figure 2—figure supplement 1—source data 2. [file elife-107023-fig2-figsupp1-data2.pdf]

Raw unedited membranes

Figure 2-Figure supplement 1C

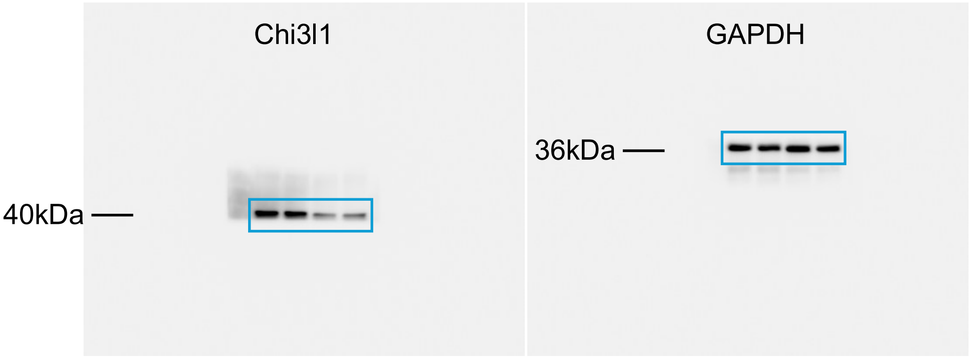

Figure 2-Figure supplement 1-Source Data 3. Original membranes corresponding to Figure 2-Figure supplement 1C. Chi3l1 expression in isolated KCs of Chi3l1$^{fl/fl}$ (first two lanes) and Chi3l1-KpKO (lanes 3 and 4) mice.
