# Supplementary material for: Differential regulation of hepatic macrophage fate by Chi3l1 in metabolic dysfunction-associated steatotic liver disease
Source: eLife. 2026 Jun 26;14:RP107023. doi: 10.7554/eLife.107023 (PMC13309125; doi:10.7554/eLife.107023)
Supplement: Figure 3—source data 2. [file elife-107023-fig3-data2.pdf]

Raw unedited membranes

Figure 3B

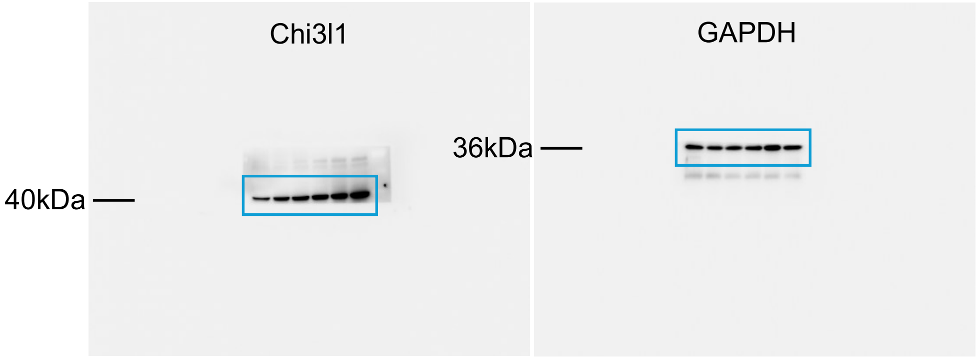

Figure 3-Source Data 2. Original membranes corresponding to Figure 3B. Chi3l1 expression in whole liver tissues under NCD (first three lanes) and MCD (lanes 4, 5, and 6) diets.
