# Supplementary material for: Differential regulation of hepatic macrophage fate by Chi3l1 in metabolic dysfunction-associated steatotic liver disease
Source: eLife. 2026 Jun 26;14:RP107023. doi: 10.7554/eLife.107023 (PMC13309125; doi:10.7554/eLife.107023)
Supplement: Figure 3—figure supplement 1—source data 2. [file elife-107023-fig3-figsupp1-data2.pdf]

Raw unedited membranes

Figure 3-Figure supplement 1C-BMDM

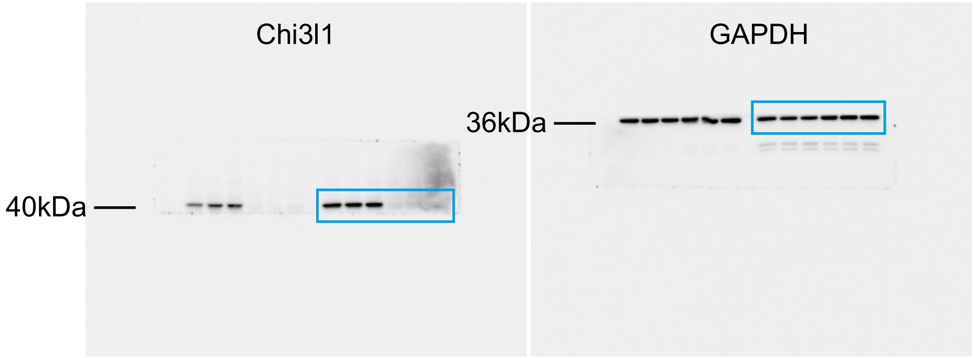

Figure 3-Figure supplement 1C -KCs

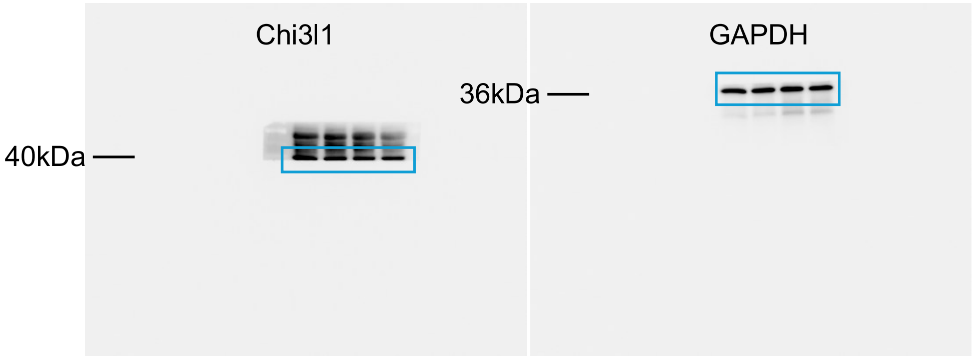

Figure 3-Figure supplement 1-Source Data 5. Original membranes corresponding to Figure 3-Figure supplement 1C. Chi3l1 in BMDM (Figure 1C) from Chi3l1$^{fl/fl}$ and Chi3l1-MKO mice. The lower membranes show primary KCs (Figure 1C) from Chi3l1$^{fl/fl}$ and Chi3l1-MKO mice.
