# Supplementary material for: Differential regulation of hepatic macrophage fate by Chi3l1 in metabolic dysfunction-associated steatotic liver disease
Source: eLife. 2026 Jun 26;14:RP107023. doi: 10.7554/eLife.107023 (PMC13309125; doi:10.7554/eLife.107023)
Supplement: Figure 6—source data 2. [file elife-107023-fig6-data2.pdf]

Raw unedited membranes

Figure 6D

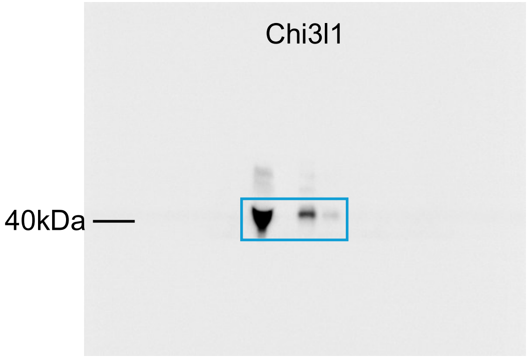

Figure 6F

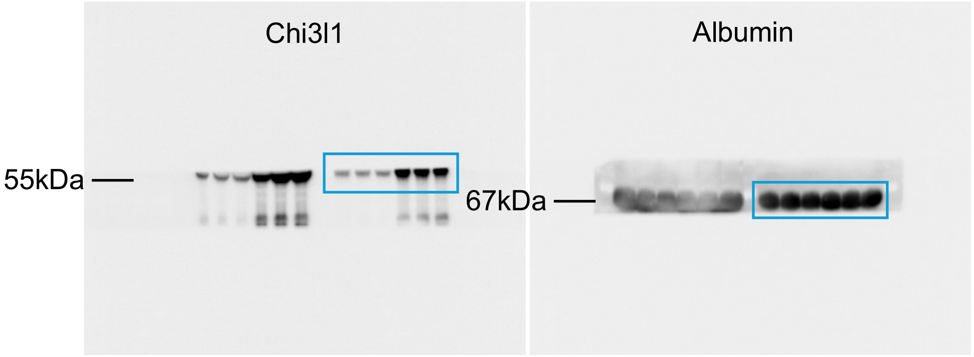

Figure 6-Source Data 2. Original membranes corresponding to Figure 6D and 6F. Western blot was performed to examine Chi3l1 in the precipitate(Figure 6D). Biotin-conjugated glucose was incubated with murine serum from mice fed with HFHC for 16 weeks (lane 3). Biotin or biotin-conjugated glucose plus glucose were used as negative controls (lane 4). Lower membranes show murine serum (Figure 6F) before (first three lanes) and after (lanes 4, 5, and 6) HFHC feeding.
