# Supplementary material for: Differential regulation of hepatic macrophage fate by Chi3l1 in metabolic dysfunction-associated steatotic liver disease
Source: eLife. 2026 Jun 26;14:RP107023. doi: 10.7554/eLife.107023 (PMC13309125; doi:10.7554/eLife.107023)
Supplement: Figure 7—source data 2. [file elife-107023-fig7-data2.pdf]

Raw unedited membranes

Figure 7E

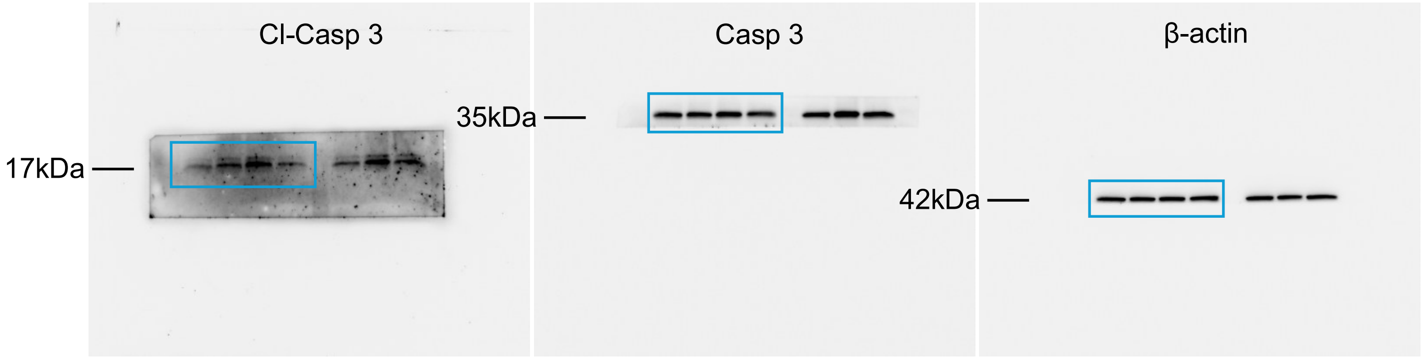

Figure 7-Source Data 2. Original membranes corresponding to Figure 7E. Western blot was performed to detect cleaved caspase 3 (Cl-Casp3) and Casp3. KCs were treated without (blank, lane 1) or with either Isopropyl alcohol (Iso, lane 2) or 800uM palmitic acid (PA, lane 3) or 100ng rChi3l1 with 800 uM PA(lane 4) for 24 h.
