# Supplementary material for: Differential regulation of hepatic macrophage fate by Chi3l1 in metabolic dysfunction-associated steatotic liver disease
Source: eLife. 2026 Jun 26;14:RP107023. doi: 10.7554/eLife.107023 (PMC13309125; doi:10.7554/eLife.107023)
Supplement: Supplementary file 1. [file elife-107023-supp1.docx]

**Supplementary file 1**

**PCR primer sequences for genotyping**

| Aim genes | PCR No. | Primer No. | Sequence | Band Size |
| --- | --- | --- | --- | --- |
| *Chi3l1^flox//flox^* | 1. 5’arm | F1(JS05000-*Chi3l1*-5wt-tF1) | CTTGTTCAGCCAAGGTGATGGGTA | WT:270bp targeted:375bp |
|  |  | R1(JS05000-*Chi3l1*-5wt-tR1) | CTACCTGATTGCTGGGGCTCATTA |  |
|  | 1. 3’arm | F2(JS15000-*Chi3l1*-3wt-F1) | CCAGTATTTAGAGGCAGAGAGATGGTG | WT:283bp targeted:384bp |
|  |  | R2(JS15000-*Chi3l1*-3wt-R1) | CTCGAATTCAGAAATCTGCCTGCCT |  |
| *Chi3l1^-/-^* |  | F1(JS05000-*Chi3l1*-5wt-tF1) | CTTGTTCAGCCAAGGTGATGGGTA | WT: 2960 bp  Targeted: ~256 bp |
|  |  | R1(JS05000-*Chi3l1*-3wt-tR1) | CTCGAATTCAGAAATCTGCCTGCCT |  |
|  |  | F2(JS15000-*Chi3l1*-wt-F1) | CTGTTAGTTGCACCTTGGAGCAGTCA | WT: 309 bp  Targeted:0 bp |
|  |  | R2(JS15000-*Chi3l1*-wt-R1) | CAGATATAGGAGAACATCCAGTCTGGG |  |
| *Clec4f*  *Cre* | ①5'arm | GPS00003712-*Clec4f*-wt-tF1 | CCCATCCTGAGGTCTCTTTATGC | WT:0bp Targeted:258bp |
|  |  | IRES-tR2 | TAGAGTCCAGATCTTCCGGGTAC |  |
|  | ②3'arm | iCre-tF1 | GGCTGGACCAATGTGAACATTG | WT:0bp Targeted:265bp |
|  |  | GPS00003712-*Clec4f*-wt-tR1 | TATTGAGGGCTTATCTGGGCAG |  |
|  | ③WT | GPS00003712-*Clec4f*-wt-tF1A | GGAGAGCGAGAAGACTGTGTTCAC | WT:250bp Targeted:1927bp |
|  |  | GPS00003712-*Clec4f*-wt-tR1A | GACTCCAATGCAGGGCTTGTCT |  |
| *Lyz2*  *Cre* | ①5'arm | F1 | AGTGCTGAAGTCCATAGATCGG | WT:0bp Targeted:258bp |
|  |  | R1 | CTGATTCTCCTCATCACCAGG |  |
|  | ②WT | F2 | AGTGCTGAAGTCCATAGATCGG | WT:0bp Targeted:265bp |
|  |  | R2 | GTCACTCACTGCTCCCCTGT |  |
