# Supplementary material for: Differential regulation of hepatic macrophage fate by Chi3l1 in metabolic dysfunction-associated steatotic liver disease
Source: eLife. 2026 Jun 26;14:RP107023. doi: 10.7554/eLife.107023 (PMC13309125; doi:10.7554/eLife.107023)
Supplement: Supplementary file 2. [file elife-107023-supp2.docx]

**Supplementary File 2**

**qPCR primers**

| Targeted gene | Primer | Sequence |
| --- | --- | --- |
| musChi3l1 | F(5‘-3’) | AGAAACACCAACCTGAAGACC |
|  | R(5‘-3’) | CCCATCAAAGCCATAAGAACG |
