# Supplementary material for: Differential regulation of hepatic macrophage fate by Chi3l1 in metabolic dysfunction-associated steatotic liver disease
Source: eLife. 2026 Jun 26;14:RP107023. doi: 10.7554/eLife.107023 (PMC13309125; doi:10.7554/eLife.107023)
Supplement: Peer review history

# Editor report: eLife Assessment

**DOI:** 10.7554/eLife.107023.4.sa0

**Authors:** Simon Yona

**Affiliations:** The Hebrew University of Jerusalem Israel

**Keywords:** Solid, Useful

This study provides an insight into the role of a Chi3l1 in liver macrophages during metabolic disease. The evidence is **solid** with the authors now addressing most concerns, although one key conclusion is not fully supported by the data presented. Overall, the work offers a **useful** contribution to the field.

# Referee report: Reviewer #2 (Public review)

**DOI:** 10.7554/eLife.107023.4.sa1

In this revised version of the manuscript, the authors have addressed many of my concerns. The representative confocal images now provided, allow for a much better assessment of the claims being made and hence the data to be understood, for example the level of protein expression of Chi3l1 in the macrophages.

There is just 1 concern remaining, which is a main claim of the manuscript, that loss of Chi3l1 drives KC death in MASLD. This claim is made based on gene expression profiles and the presence of Tunel staining in liver sections. However the KC numbers are not altered compared with WT when assessed by flow cytometry. This discrepancy is not really addressed. If the cells are not actually dying this would explain the lack of moKCs (a concern raised by reviewer 1) and would indeed suggest that the loss of these cells is, as suggested by that reviewer, trivial in this timeframe. The authors propose in their rebuttal that the KCs are in a prolonged state of stress, explaining the Tunel staining, but to make the claim that they die, the authors need to show their eventual loss from the liver. Otherwise the claims of death should be revised.

# Referee report: Reviewer #3 (Public review)

**DOI:** 10.7554/eLife.107023.4.sa2

This paper investigates the role of Chi3l1 in regulating the fate of liver macrophages in the context of metabolic dysfunction leading to the development of MASLD.

Comments on revisions:

My comments have been addressed.

# Author comment: Author response

**DOI:** 10.7554/eLife.107023.4.sa3

**Authors:** Jia He, Bo Chen, Weiju Lu, Xiong Wang, Ruoxue Yang, Chengxiang Deng, Xiane Zhu, Keqin Wang, Lang Wang, Cheng Xie, Rui Li, Xiaokang Lu, Ruizhi Yang, Cheng Peng, Canpeng Li, Zhao Shan

**Affiliations:** Yunnan University Kunming China; Yunnan University Kunming China; Yunnan University Kunming China; Yunnan University kunming China; Yunnan University Kunming China; Yunnan University kunming China; Yunnan University kunming China; Yunnan University Kunming China; Yunnan University kunming China; Yunnan University Kunming China; Yunnan University Kunming China; Yunnan University kunming China; Yunnan University Kunming China; Yunnan University kunming China; Yunnan University kunming China; Yunnan University Kunming China

The following is the authors’ response to the previous reviews

**Public Reviews:**

**Reviewer #1 (Public review):**

The manuscript by Shan et al seeks to define the role of the CHI3L1 protein in macrophages during the progression of MASH. The authors argue that the Chil1 gene is expressed highly in hepatic macrophages. Subsequently, they use Chil1 flx mice crossed to Clec4F-Cre or LysM-Cre to assess the role of this factor in the progression of MASH using a high fat high, fructose diet (HFFC). They found that loss of Chil1 in KCs (Clec4F Cre) leads to enhanced KC death and worsened hepatic steatosis. Using scRNA seq they also provide evidence that loss of this factor promotes gene programs related to cell death. From a mechanistic perspective they provide evidence that CHI3L serves as a glucose sink and thus loss of this molecule enhances macrophage glucose uptake and susceptibility to cell death. Using a bone marrow macrophage system and KCs they demonstrate that cell death induced by palmitic acid is attenuated by the addition of rCHI3L1. While the article is well written and potentially highlights a new mechanism of macrophage dysfunction in MASH and the authors have addressed some of my concerns there are some concerns about the current data that continue to limit my enthusiasm for the study. Please see my specific comments below.

Major:

(1) The authors' interpretation of the results from the KC (Clec4F) and MdM KO (LysMCre) experiments is flawed. The authors have added new data that suggests LyM-Cre only leads to a 40% reduction of Chil1 in KCs and that this explains the difference in the phenotype compared to the Clec4F-Cre. However, this claim would be made stronger using flow sorted TIM4hi KCs as the plating method can lead to heterogenous populations and thus an underestimation of knockdown by qPCR. Moreover, in the supplemental data the authors show that Clec4f-Cre x Chil1flx leads to a significant knockdown of this gene in BMDMs. As BMDMs do not express Clec4f this data calls into question the rigor of the data. I am still concerned that the phenotype differences between Clec4f-cre and LyxM-cre is not related to the degree of knockdown in KCs but rather some other aspect of the model (microbiota etc). It woudl be more convincing if the authors could show the CHI3L reduction via IF in the tissue of these mice.

We thank the reviewer for these constructive comments. We have performed FACSsorting of KCs (CD45^+^ F4/80^hi^ CD11b^low^ TIM4^hi^) or MoMFs (CD45^+^ F4/80^low^ CD11b^hi^ Ly6G^-^ TIM4^-^) from Chil1^fl/fl^ and *Lyz2^∆Chil1^* or *Clec4f^∆Chil1^*mice, respectively. Compared with *Chil1^fl/fl^* mice, mRNA levels of *Chil1* was reduced more than 90% in KCs from *Clec4f^∆Chil1^* mice while not different in MoMFs (Revised Figure S3B). Besides, compared with *Chil1^fl/fl^* mice, mRNA levels of Chil1 was reduced more than 90% in MoMFs from *Lyz2^∆Chil1^* mice while roughly 40% in KCs (Revised Figure S5B). This revised data support the phenotypic difference between Lyz2-CKO and Clec4f-CKO mice.

We agree with the reviewer that the significant knockdown of *Chil1* in BMDM from *Clec4f^∆Chil1^*mice is confusing. To keep the rigor of our data, we remove this part from our manuscript.

Additionally, we performed immunofluorescence staining to detect Chi3l1 expression in liver tissues of these mice. The results show a reduction of Chi3l1 expression in KCs (TIM4+F4/80+ cells) of both *Lyz2^∆Chil1^*and *Clec4f^∆Chil1^*mice, with a more pronounced decrease in *Clec4f^∆Chil1^*mice (Author response image 1).

**Author response image 1.** The expression of Chi3l1 in liver tissues of *Chil1^fl/fl^*, *Lyz2^∆Chil1^*and *Clec4f^∆Chil1^*mice. Immunofluorescent staining to detect Chi3l1 (green) expression in liver sections of *Chil1^fl/fl^*, *Lyz2^∆Chil1^*and *Clec4f^∆Chil1^*mice under normal chow diet. TIM4 (KCs marker, white), F4/80 (macrophage marker, red), nuclei were counterstained with DAPI, Scale bar=20 µm and 10 µm (inset).

(2) Figure 4 suggests that KC death is increased with KO of Chil1. The authors have added new data with TIM4 tht better characterizes this phenotype. The lack of TIM4 low, F4/80 hi cells further supports that their diet model is not producing any signs of the inflammatory changes that occur with MASLD and MASH. This is also supported by no meaningful changes in the CD11b hi, F4/80 int cells that are predominantly monocytes and early Mdms. It is also concerning that loss of KCs does not lead to an increase in Mo-KCs as has been demonstrated in several studies (PMID37639126, PMID:33997821). This would suggest that the degree of resident KC loss is trivial.

We appreciate the reviewer’s insightful comment. We agree that our data show no substantial generation of monocyte-derived Kupffer cells (MoKCs) within the 16-week HFHC model. However, we do not believe the degree of resident KC loss is trivial, since 60% of KCs die at 16 weeks compared with 0 week (Revised Figure 5D). Instead, our observations align with a phased replacement model: recruited monocytes first differentiate into monocyte-derived macrophages (MoMFs), which we see accumulate (Revised Figure 5D), and only later adopt a KC phenotype. Consistent with this, our 16-week model shows significant EmKC loss and MoMFs expansion, but not yet the emergence of TIM4-MoKCs. This timing is supported by prior studies, where TIM4KCs were observed at 24 weeks, but not at 16 weeks, on similar diets (PMID: 33440159; PMID: 32888418). Therefore, we interpret our findings as capturing an earlier phase of MASLD progression, characterized by EmKC death and MoMF accumulation, prior to their full differentiation into MoKCs.

(3) The authors demonstrated that Clec4f-Cre itself was not responsible for the observed phenotype, which mitigates my concerns about this influencing their model.

We thank the reviewer for this comment and are pleased they agree that our control experiment using Clec4f-Cre alone confirms that the phenotype is specific to our genetic manipulation and not an artifact of the Cre driver.

(4) I remain somewhat concerned about the conclusion that Chil1 is highly expressed in liver macrophages. The author agrees that mRNA levels of this gene are hard to see in the datasets; however, they argue that IF demonstrates clear evidence of the protein, CHI3L. The IF in the paper only shows a high power view of one KC. I would like to see what percentage of KCs express CHI3L and how this changes with HFHC diet. In addition, showing the knockout IF would further validate the IF staining patterns.

We thank the reviewer for their thoughtful and constructive feedback. We agree that our initial conclusion regarding Chil1 expression in liver macrophages relied heavily on prior observations and was not sufficiently supported by the data presented. In response, we have revised our conclusion to state: "Hepatic macrophages express Chi3l1 and upregulate its expression following HFHC feeding." (Revised manuscript, page 4, line 136-137)

To strengthen this finding, we have replaced the original high-power image of a single Kupffer cell with a representative low-power view showing multiple F4/80+ macrophages (Revised Figure 1A). Furthermore, we performed quantitative colocalization analysis, which revealed that under normal chow diet (NCD), approximately 8% of F4/80+ macrophages are Chi3l1-positive. This proportion significantly increases to 15% upon HFHC feeding (Revised Figure 1A).

Additionally, to validate the specificity of the Chi3l1 immunofluorescence signal, we have included staining of liver sections from Chil1 knockout mice. In contrast to wildtype mice, Chi3l1 signal was completely absent within F4/80+ macrophages in *Chil1^-/-^* mice, confirming the specificity of the staining (Revised Figure 1B, Revised manuscript, page 4, line 152-157).

Minor:

(1) The authors have answered my question about liver fibrosis. In line with their macrophage data their diet model does not appear to induce even mild MASH.

We thank the reviewer for this observation. We agree that under our HFHC dietary conditions, the mice do not develop MASH pathology. However, we believe this earlystage model is a strength of our study, as it allows us to dissect the initial role of the Chi3l1-glucose interaction in regulating Kupffer cell fate during early MASLD, prior to the onset of significant fibrosis. This approach enables us to capture early macrophage adaptations (such as Chi3l1 upregulation) that might otherwise be masked or become secondary to the overt inflammation and scarring characteristic of late-stage MASH models.

**Reviewer #2 (Public review):**

In the revised version of the manuscript, the authors have attempted to address my questions, however, a number of my original concerns still remain.

Firstly, I had asked for a validation of the different CRE lines used - Lysm and Clec4f. The authors have now looked at BMDMs and KCs (steady state) from these animals. They conclude LysM only targets BMDMs not KCs, while CLEC4F targets both KCs and BMDMs. This I do not understand, BMDMs do not express CLEC4F so why are they targeted with this CRE? Additionally, BMDMs are not the correct control here, rather the authors should look at the incoming moMFs in the livers of these mice in the MASLD setting. Similarly, the KO in the MASLD KCs should be verified.

We thank the reviewer for these constructive comments. We have performed FACSsorting of KCs (CD45^+^ F4/80^hi^ CD11b^low^ TIM4^hi^) or MoMFs (CD45^+^ F4/80^low^ CD11b^hi^ Ly6G^-^ TIM4^-^) from *Chil1^fl/fl^* and *Lyz2^∆Chil1^* or *Clec4f^∆Chil1^*mice fed NCD or HFHC for 4 weeks, respectively. Compared with *Chil1^fl/fl^* mice, mRNA levels of Chil1 was reduced more than 90% in KCs from *Clec4f^∆Chil1^* mice while not different in MoMFs at both 0 and 4 weeks (Revised Figure S3B). Besides, compared with *Chil1^fl/fI</sup^* mice, mRNA levels of *Chil1^fl/fI</sup^* was reduced more than 90% in MoMFs from *Lyz2^∆Chil1^* mice while roughly 40% in KCs at both 0 and 4 weeks (Revised Figure S5B). This revised data support the phenotypic difference between Lyz2-CKO and Clec4f-CKO mice.

Then I had asked for validation of macrophage expression of Chil1 in other MASLD human and mouse datasets. The authors have looked into this, but the data provided do not suggest it is highly expressed by these cells either in the other mouse models or in the human. Nevertheless, they include a statement suggesting a similar expression pattern (although also being expressed by other cells). This is not an accurate discussion of the data and hence must be revised. This also prompted me to take another look at their data and this has left me querying the data in Figure 1D. Is the percent expressed 1%? In Figure 1C the scale goes from 0-100 but here 0-1. If we are talking about expression in 1% of cells which would fit with the additional public mouse data now analysed then how relevant are any of these claims? How sure are the authors that the effects seen are through KCs/moMFs? In figure 1D all cells profiled by scRNA-seq should be shown not just MFs to get a better sense of this data. What is macrophage expression of Chil1 compared with all other liver cells?

We thank the reviewer for the thoughtful feedback. We agree that the expression pattern of Chil1 should be described more accurately. To address this point, we examined four additional publicly available scRNA-seq datasets, including two mouse MASLD models and two human MASLD datasets (Author response image 2). Across these studies, the cell type with the highest Chil1 expression varied, whereas Chil1 transcripts were detected at relatively low frequency in macrophages (~1% of cells; Author response image 2C, E, K). To better present these data, we regenerated the UMAP plots to include all captured liver non-parenchymal cells, defined using the top two lineage specific markers (Author response image 3A–B). Consistent with Figure 2A–C, violin plots show that Chil1 is highly expressed in neutrophils, with only modest expression detected in macrophages (Author response image 3C). Further analysis of monocyte/macrophage subsets indicates that approximately ~1% of MoMFs or KCs express Chil1 (Author response image 3D–F). As the reviewer noted, the y-axis in Author response image 3F ranges from 0–1%, reflecting the low transcriptional detection frequency of Chil1 in macrophages, which is consistent with the additional public datasets analyzed.

We also recognize that mRNA detection by scRNA-seq does not necessarily reflect protein abundance. Therefore, we assessed Chi3l1 protein expression in hepatic macrophages using immunofluorescence staining for F4/80, TIM4, and Chi3l1 in liver sections from mice fed either normal chow diet (NCD) or HFHC diet. These analyses show that Chi3l1 protein is detectable in both KCs (TIM4^+^F4/80^+^) and MoMFs (TIM4^-^F4/80^+^) (Revised Figure 1A). Quantitative colocalization analysis revealed that under NCD conditions, approximately 8% of F4/80^+^ macrophages are Chi3l1-positive, which increases to ~15% following HFHC feeding (Revised Figure 1A). To confirm antibody specificity, we additionally performed staining in Chil1 knockout mice. In contrast to wild-type mice, Chi3l1 signal was completely absent in F4/80^+^ macrophages from Chil1^-/-^ mice, validating the specificity of the staining (Revised Figure 1B). Together, these results suggest that low-abundance Chil1 transcripts may be under-detected by scRNA-seq, whereas immunofluorescence captures accumulated protein. Importantly, our functional experiments using Clec4f-Cre– mediated deletion directly support that the observed phenotypes are mediated through Kupffer cells, regardless of expression levels in other liver cell types.

In response to the reviewer’s comments, we have made the following revisions:

(1) Softened our conclusion to: “Hepatic macrophages express CHI3L1 and upregulate its expression following HFHC feeding” (Revised manuscript, page 4, lines 136–137).

(2) Included representative low-magnification images showing multiple F4/80^+^ macrophages along with quantitative analysis (Revised Figure 1A).

(3) Added immunofluorescence staining of Chil1^-/-^ liver sections demonstrating complete absence of Chi3l1 signal in F4/80^+^ macrophages, validating antibody specificity (Revised Figure 1B).

(4) Regenerated UMAP plots to display all liver non-parenchymal cells and clearly indicate the low detection frequency of Chil1 transcripts in macrophages (Author response image 3).

(5) Revised the relevant text to more accurately describe Chil1 expression patterns in hepatic macrophages (Revised manuscript, page 4, lines 136–157).

**Author response image 2.** Analysis of Chil1 expression in additional single-cell RNA sequencing datasets. (A-C) Chil1 expression in a mouse model of NASH. (A) t-SNE projection of cell clusters from scRNA-seq data (GSE1283338) of livers from C57BL/6J mice fed a control or NASH diet for 30 weeks. (B) Dot plot showing scaled Chil1 expression across all identified cell clusters. (C) Dot plot of scaled Chil1 expression after excluding the neutrophil cluster, highlighting expression in macrophage populations. Analyzed cell clusters and cell numbers: KC_H (healthy, 1178); KC3_Control (1142); KC_N (NASH, 1045); KN_RM (recruited macrophage in KC niche, 950); Proliferating_KC (364); PDC_Control (356); Ly6CHi_RM (320); LSEC (299); NK_NKT (393); B_cell (244); DC_1 (107); DC_2 (118); Ly6CLo_RM (127); Hepatocyte (57); PDC_NASH (46); Neutrophil (21). (D-E) Chil1 expression during NAFLD progression in a mouse Western diet model. (D) t-SNE projection of cell clusters from scRNA-seq data (GSE156059) of livers from C57BL/6J mice fed a Western diet with fructose/sucrose for 12, 24, and 36 weeks. (E) Dot plot showing scaled Chil1 expression across all identified cell clusters. Analyzed cell clusters and cell numbers: capsule macs (250), LAMs (1419), Ly6chi monocytes (6912), mac1 (638), moKCs (767), Patrolling monocytes (690), Prolif.macs (521), Resident KCs (3629), Transitioning monocytes (3615). (F-H) Chil1 expression in human cirrhotic liver biopsies. (F) t-SNE projection of cell clusters from scRNA-seq data (GSE136103) of healthy and cirrhotic human liver samples. (G) Dot plot showing scaled Chil1 expression across major cell lineages. (H) Dot plot of scaled Chil1 expression specifically within the mononuclear phagocyte (MP) population. Analyzed cell clusters and cell numbers: B cell (1951); cycling (967); Epithelia (3751); ILC (10091); mast cell (2511); Mesenchyme (2382); MP (10874); pDC (317); Plasma cell (877); T cell (19076). (I-K) Chil1 expression in a human NAFLD explant. (I) t-SNE projection of cell clusters from scRNA-seq data (GSE190487) of a human NAFLD liver explant. (J) Dot plot showing scaled Chil1 expression across all identified cell clusters. (K) Dot plot of scaled Chil1 expression within the MP subpopulations. Analyzed cell clusters and cell numbers: B cell (1278); Cycling (152); MP (2897); pDC (391); Plasma cell (85); T cell (1551); KC (403); SAMac (scar-associated macrophages, 723); TM (tissue monocytes, 1265).

**Author response image 3.** Hepatic macrophages express Chi3l1. (A-D) Wildtype C57BL/6J mice were fed either a normal chow diet (NCD) or HFHC for 16 weeks. NPCs were isolated and subjected to BD Rhapsody scRNA sequencing. (A) Uniform manifold approximation and projection (UMAP) plots illustrate the clustering of NPCs from the livers of mice fed NCD and HFHC. Major cell types are colored. (B) Heatmap showing the mean expression of top2 markers of each cell type. (C) Violin plots show the RNA expression of Chil1 between NCD and HFHC livers in each cell cluster. (D) UMAP plots depict the clustering of Monocytes/Macrophages in the livers of mice fed NCD and HFHC. Cell clusters are color-coded. (E) Dot plot displays the scaled gene expression levels of lineage-specific marker genes in different cell clusters. (F) Dot plot shows the scaled gene expression levels of Chil1 in the indicated cell clusters.

The cell death had also previously concerned me that 40-60% of KCs were tunel +ve. I do not understand how 60% are +ve at 8 weeks but then they have more or less same number of TIM4+ cells at 16 weeks? How can this be? why do the tunel +ve cells not die? This concern remains as I don't understand how they reached these numbers given the images. Additional, larger images were also not provided to be sure that they are representative images in the figure. Now in the images provided, there are clearly cells which are TIM4+ where the tunel does not overlap, likely it is in a LSEC or other neighbouring cell. Indeed also taking Fig S11b as an example there are ˜7KCs and at best 1 expresses tunel so how do they get to 60%?

We thank the reviewer for these constructive feedback. We agree that the sustained TUNEL positivity without corresponding KC depletion presents an apparent paradox. Based on our data, we propose that TUNEL-positive KCs represent cells in a prolonged stressed or pre-apoptotic state rather than undergoing immediate clearance. This interpretation is supported by the relatively stable TIM4+ cell numbers between 8 and 16 weeks, which would be inconsistent with rapid cell death and removal. Previous studies (PMID: 33440159; PMID: 32888418) have similarly documented gradual KC loss during MASLD progression, supporting our view that KC death occurs over an extended timeframe rather than acutely.

Regarding quantification concerns, we acknowledge that the representative images in the original figure may have been misleading. To address this, we have now quantified KC apoptosis using low-magnification fields across multiple liver sections to ensure statistical rigor. Figure S11B (now Revised Figure S9B) presents these data, showing that under NCD conditions, KC apoptosis rates are minimal in both genotypes. Following HFHC feeding, apoptosis rates are comparable between Chil1^fl/fl^ and Lyz2^Δ Chil1^ mice. Importantly, we have replaced all TIM4/TUNEL co-staining images with lowmagnification representative images in the revised figures (Revised Figure 1A, 1B, 5E, S9A, S9B). These images better reflect the quantitative data and confirm that the originally highlighted high-magnification fields were not representative of global apoptosis rates.

**Reviewer #3 (Public review):**

This paper investigates the role of Chi3l1 in regulating the fate of liver macrophages in the context of metabolic dysfunction leading to the development of MASLD. I do see value in this work, but some issues exist that should be addressed as well as possible.

Here are my comments:

(1) Chi3l1 has been linked to macrophage functions in MASLD/MASH, acute liver injury, and fibrosis models before (e.g., PMID: 37166517), which limits the novelty of the current work. It has even been linked to macrophage cell death/survival (PMID:31250532) in the context of fibrosis, which is a main observation from the current study.

We thank the reviewer for raising this important point and acknowledge previous studies linking Chi3l1 to macrophage function in liver disease. However, several aspects of our work extend beyond these prior reports. First, although global Chi3l1 deficiency has been shown to promote macrophage apoptosis in toxin-induced fibrosis models (PMID: 31250532), our study demonstrates that Chi3l1 differentially regulates the fate of distinct hepatic macrophage subsets embryo-derived Kupffer cells (KCs) and monocyte-derived macrophages (MoMFs)—in MASLD. To our knowledge, this subset-specific regulation of hepatic macrophages has not been previously described. Second, we identify a previously unrecognized metabolic mechanism by which Chi3l1 regulates macrophage survival. Specifically, we find that Chi3l1 binds glucose and promotes glucose uptake, thereby protecting the highly glucose-dependent KCs from metabolic stress–induced death, while exerting minimal effects on MoMFs. This mechanism is distinct from the previously reported Fas/Akt-mediated pathway (PMID: 31250532) and highlights a metabolic checkpoint controlling macrophage subset– specific vulnerability. Third, our findings reveal context- and cell type-dependent roles of Chi3l1. While myeloid-specific deletion of Chi3l1 has been reported to ameliorate steatohepatitis and fibrosis (PMID: 37166517), our KC-specific deletion model shows that loss of Chi3l1 in KCs exacerbates disease, indicating a previously unrecognized protective role of Chi3l1 in KCs during early MASLD. Together, these findings provide new insights into macrophage subset-specific regulation, identify a novel glucose related metabolic mechanism, and reveal context-dependent functions of Chi3l1 in MASLD pathogenesis.

(2) The LysCre-experiments differ from experiments conducted by Ariel Feldstein's team (PMID: 37166517). What is the explanation for this difference? - The LysCre system is neither specific to macrophages (it also depletes in neutrophils, etc), nor is this system necessarily efficient in all myeloid cells (e.g., Kupffer cells vs other macrophages). The authors need to show the efficacy and specificity of the conditional KO regarding Chi3l1 in the different myeloid populations in the liver and the circulation.

We thank the reviewer for raising this important point regarding the specificity of the genetic models and the apparent discrepancy with the study by Feldstein and colleagues (PMID: 37166517). To address these concerns, we performed additional experiments to directly assess the efficiency and cell-type specificity of Chi3l1 deletion in our models.

(1) Efficiency and specificity of LysM-Cre and Clec4f-Cre models

We isolated KCs (CD45^+^ F4/80^hi^ CD11b^low^ TIM4^hi^) or MoMFs (CD45^+^ F4/80^low^ CD11b^hi^ Ly6G^-^ TIM4^-^) by FACS from *Chil1^fl/fl^*, *Lyz2^∆Chil1^* and *Clec4f^∆Chil1^*mice fed either NCD or HFHC diet. Consistent with the known specificity of these Cre lines, Clec4f-Cre resulted in >90% reduction of *Chil1* mRNA in KCs with no significant change in MoMFs (Revised Figure S3B), confirming efficient KC-specific deletion. In contrast, LysM-Cre reduced Chil1 expression by >90% in MoMFs but only ~40% in KCs (Revised Figure S5B). These data support the reviewer’s concern that LysM-Cre mediates incomplete recombination in KCs, whereas the Clec4f-Cre model provides KC-specific deletion, explaining why the phenotype observed in *Lyz2^∆Chil1^* mice is relatively modest.

(2) Relationship to the study by Feldstein et al.

We agree that our LysM-Cre results appear different from those reported by Feldstein and colleagues. However, considering the new recombination data and differences in disease models, we believe the findings are complementary rather than contradictory. First, the disease models differ substantially. Feldstein et al. used a CDAA-HFAT diet for 10 weeks, which rapidly induces severe inflammation and fibrosis, whereas our study employed a long-term HFHC diet, modeling the more gradual metabolic progression of MASLD. These distinct disease contexts may engage different CHI3L1dependent pathways. Second, the mechanistic focus differs. Feldstein et al. reported that myeloid Chi3l1 promotes steatohepatitis and fibrosis through inflammatory macrophage recruitment and IL13Rα2-mediated stellate cell activation. In contrast, our study identifies a metabolic mechanism in which CHI3L1 binds glucose and promotes glucose uptake, protecting metabolically vulnerable KCs from stress-induced death. Finally, and importantly, KC-specific deletion using Clec4f-Cre recapitulates the key phenotypes observed in our study, including effects on KC survival and metabolic regulation. This confirms that the observed effects are KC-autonomous and not due to broader Cre activity in other myeloid populations.

Together, these additional experiments clarify the recombination efficiency of our models and demonstrate that our conclusions are supported by KC-specific genetic evidence.

(3) The conclusions are exclusively based on one MASLD model. I recommend confirming the key findings in a second, ideally a more fibrotic, MASH model.

We thank the reviewer for this valuable suggestion. To address this point, we tested our key findings in an additional MASH model using a methionine–choline-deficient (MCD) diet. First, we examined Chi3l1 expression in this model. Wild-type mice fed an MCD diet for 6 weeks showed significantly increased Chi3l1 mRNA and protein levels in liver tissues compared with NCD controls, confirming diet-induced upregulation (Revised Figure 3A–B). To determine the functional contribution of Kupffer cell–derived Chi3l1, we subjected *Clec4f^ΔChil1^* mice and *Chil1^fl/fl^* controls to MCD feeding for 6 weeks. Body weight was comparable between genotypes throughout the feeding period (Revised Figure 3C). However, KC-specific deletion of Chi3l1 significantly exacerbated MCD diet–induced liver pathology, including increased steatosis, inflammation, and fibrosis, as indicated by higher MASLD activity scores, enhanced Oil Red O staining, increased Sirius Red deposition, and elevated α-SMA expression (Revised Figure 3D). Consistent with these histological findings, *Clec4f^ΔChil1^* mice exhibited an increased liver index, whereas serum ALT levels remained comparable between groups, suggesting increased hepatic lipid accumulation rather than aggravated hepatocellular injury (Revised Figure 3E). In addition, serum and hepatic triglyceride levels and serum cholesterol were significantly elevated, while hepatic cholesterol levels were not significantly different from controls (Revised Figure 3E). Together, these results validate our findings in an independent MASH model and further support a protective role for Kupffer cell–derived Chi3l1 in limiting steatosis and disease progression (Revised manuscript, page 5, line 188-205).

(4) Very few human data are being provided (e.g., no work with own human liver samples, work with primary human cells). Thus, the translational relevance of the observations remains unclear.

We thank the reviewer for raising this important point. We agree that additional human validation would further strengthen the translational relevance of our findings. We initially attempted to examine macrophage cell death in human liver samples by performing TUNEL and F4/80 co-staining on human liver cancer tissues. However, we did not detect clear colocalization in these samples. We speculate that this may reflect differences in disease context and stage, as the available samples represent endstage liver disease, whereas our study focuses on early MASLD progression. Despite this limitation, we provide several lines of evidence supporting the human relevance of our findings. First, analysis of multiple public human MASLD scRNA-seq datasets demonstrates Chi3l1 expression in hepatic macrophages (Figure 2F–K). Second, analysis of public bulk RNA-seq datasets shows that Chi3l1 expression positively correlates with MASLD disease activity and progression (Revised Figure 1EF). Third, our observations are consistent with previous clinical studies reporting elevated CHI3L1 levels in patients with MASLD/MASH and advanced liver disease. We acknowledge that functional validation in primary human macrophages or human liver tissues would further strengthen the translational significance of this work. This limitation and future direction have now been added to the Discussion (Revised manuscript, page 10, lines 409–411).

Comments on revisions:

The authors have done a thorough job addressing my comments. However, I am not convinced about the MCD diet model, which is somewhat hidden in the Supplementary Files. Neither seems MASH different nor are any fibrosis data shown to support the conclusions. I am not satisfied with this part of the revised manuscript, and I do not agree that the second MASH model would support the conclusions.

We thank the reviewer for their continued careful evaluation and for highlighting the need for clearer presentation of the MCD model data. To address this concern, we have substantially revised this section of the manuscript. First, the MCD model results have now been moved from the Supplementary Figure to a new main figure (Revised Figure 3) to improve visibility and clarity. Second, we have added additional fibrosis analyses, including Sirius Red staining and α-SMA immunostaining, to directly assess fibrotic changes. These analyses show that MCD feeding induces significant collagen deposition in control mice and that fibrosis is further increased in *Clec4f^ΔChil1^* mice (Revised Figure 3D). Importantly, the MCD model recapitulates the key phenotypes observed in the HFHC model, with KC-specific Chi3l1 deletion leading to increased MASLD progression. These findings support the conclusion that the protective role of Kupffer cell–derived Chi3l1 is not restricted to a single dietary model, but is observed across distinct models of steatohepatitis. We hope that these revisions clarify the results and strengthen the evidence supporting our conclusions.

**Recommendations for the authors:**

**Reviewer #2 (Recommendations for the authors):**

Minor:

Line 73 - should be moMfs not moKCs

We thank the reviewer for this helpful comment. The term moKCs was used intentionally in line 73 to refer to monocyte-derived Kupffer cells, rather than MoMFs (monocyte-derived macrophages). To avoid potential confusion, we have clarified the terminology in the revised manuscript.

Methods: diet is mentioned for 6 weeks but for HFHC should be 16.

The correction has been made in the Methods section (page 3，line115).

Liver/body weight ratios are >3 then I think it is body/liver weight ratio?

We thank the reviewer for this query. The reported values represent liver-to-body weight ratios, calculated as (liver weight ÷ body weight) × 100%. A value of ~3% is consistent with the expected range for mice with MASLD-associated hepatomegaly.

This clarification has been added to the revised figure legend.

Figure 5F - what happens in Clec4f-CRE mice fed HFHC?

We thank the reviewer for this question. Western blot analysis showed that the HFHC diet upregulated Chi3l1 protein in the livers of Clec4f-Cre mice post HFHC diet (Author response image 4.), similar to the increase observed in wild-type mice.

**Author response image 4.** The expression of Chi3l1 in serum of Clec4f cre mice. (A) Western blot to detect Chi3l1 expression in murine serum of Clec4f cre mice before and after HFHC feeding. n=3 mice/group.
